# Hepatic urea, creatinine and uric acid metabolism in dairy cows with divergent milk urea concentrations

**DOI:** 10.1038/s41598-022-22536-y

**Published:** 2022-10-20

**Authors:** Marie C. Prahl, Carolin B. M. Müller, Dirk Albrecht, Franziska Koch, Klaus Wimmers, Björn Kuhla

**Affiliations:** 1 Research Institute for Farm Animal Biology (FBN), Institute of Nutritional Physiology ‘Oskar Kellner’, Wilhelm-Stahl-Allee 2, 18196 Dummerstorf, Germany; 2 Institute of Microbiology, Ernst-Moritz-Arndt University Greifswald, Felix-Hausdorff-Strasse 8, 17489 Greifswald, Germany; 3 Research Institute for Farm Animal Biology (FBN), Institute of Genome Biology, Wilhelm-Stahl-Allee 2, 18196 Dummerstorf, Germany

**Keywords:** Biochemistry, Molecular biology, Physiology

## Abstract

Milk urea concentration is an indicator for dietary nitrogen (N)-supply and urinary N-excretion. Dairy cows with high (HMU) compared to low milk urea (LMU) concentration have greater plasma urea, creatinine and uric acid concentrations, but if the liver metabolism accounts for these differences is unknown. Eighteen HMU and 18 LMU cows were fed a diet with a low (LP) or normal (NP) crude protein concentration. A N balance study was performed and a ^13^C-urea bolus was administered to measure urea pool size. Liver samples were analyzed by 2D-gel-based proteomics and RT-qPCR. Although HMU cows had a greater urea pool, plasma urea, uric acid, and hippuric acid concentrations, these differences were not associated with altered expressions of genes related to urea cycling or N-metabolism. Instead, HMU cows had higher oxidative stress levels. Conclusively, other factors than hepatic urea metabolism account for milk urea concentrations. Despite higher plasma urea concentrations and argininosuccinate synthase 1 protein expression on the LP diet, urea cycle mRNA expressions were not affected, indicating that its activity is not controlled at transcriptional level. Feeding the LP diet resulted in increased expressions of enzymes catabolizing fatty acids, but the reason remains to be investigated in future studies.

## Introduction

The urea concentration in milk of dairy cows is used as an indicator for monitoring optimal dietary crude protein supply and for assessing urinary nitrogen (**N**) excretions^1,2^. As a general role, the higher the crude protein or N intake, the higher the milk urea concentrations (**MUC**) and the higher the urinary N output, at least on a herd level^3^. Nitrogen emissions from livestock can have harmful effects on biodiversity, soil, water and air quality, as they account for 23% of global nitrous oxide and 60% of global ammonia (NH_3_) emissions^4^. Besides, an increase in MUC from 150 to 300 mg/L reduces fertility, and levels above 300 mg/L are associated with infertility of cows^5,6^. Therefore, minimizing MUC without compromising the nutritional requirements of the animals may reduce environmental burden and increase fertility.

It has been shown that MUC is a moderately heritable trait^7,8^ and proposed that selection of cows for lower MUC concentration would reduce urinary urea and N emissions and thus the environmental footprint^1^. However, we have recently demonstrated that cows with high versus low MUC do not differ in urinary urea and total N excretion^9^. Rather, the level of N intake is the primary factor determining urinary N excretion for all cows^9^. The molecular mechanisms accounting for inter-individual differences in MUC despite feeding the same diet are, however, far from clear. It has been shown that cows with high MUC have higher plasma urea and creatine concentrations and reduced renal clearance rates for urea, creatine, creatinine and uric acid, suggesting that the reabsorption capacity of the kidney plays a key role in regulating the concentration of N-metabolites in plasma and finally in milk^9^. However, it is further conceivable that the metabolic rate of the liver synthesizing urea and creatine accounts for their plasma and thus milk concentrations.

Ruminants degrade dietary crude protein in the rumen resulting, among others, in the formation of NH_3_, which in turn is absorbed and transported to the liver via the portal vein. Besides, endogenous N sources enter the liver primarily via amino acids, which are initially transaminated to glutamate before further catabolism. Glutamate is degraded by glutamate dehydrogenase (GLDH) to NH_3_, which, alike as ruminally-derived NH_3_, is converted via carbamoyl-phosphate synthetase 1 (CPS1) forming carbamoylphosphate. The latter reacts with ornithine to form citrulline; thereby the N-containing carbamoyl-group enters the urea cycle. Within the urea cycle, citrulline is converted by argininosuccinate synthase (ASS) and argininosuccinate lyase (ASL) yielding arginine, which in turn is cleaved by arginase 1 (ARG1) to form urea and regenerate ornithine. Arginine may also be converted by nitric oxide synthase (NOS) yielding citrulline^10^. Besides, arginine is also needed in the rate-limiting step of creatine biosynthesis^11^ as it reacts with glycine as catalyzed by arginine:glycine aminotransferase (GATM) to form guanidinoacetate, which is methylated in the liver via guanidinoacetate N-methyltransferase (GAMT) yielding creatine.

Apart from urea and creatine, further N metabolites excreted in urine and contributing to urinary N emissions are hippuric acid as well as end products of purine catabolism, namely uric acid and allantoin. While hippuric acid is formed in the liver to detoxify benzoic acid in the reaction with glycine and can be seen as an indicator for plant digestibility^12,13^, allantoin is formed from uric acid in the reaction catalyzed by urate oxidase (UOX) and is a good indicator of the efficiency of microbial protein synthesis^14^. Uric acid, in turn, is produced by the oxidation of xanthine via xanthine dehydrogenase (XDH) in the intestinal mucosa, liver and blood^15^. We hypothesize that dairy cows with intrinsically high MUC have an increased hepatic urea cycle activity and activation of pathways producing non-urea N-metabolites, and that this increase is independent of dietary crude protein level. Recently, we have shown that a proteomics based 2D-gel electrophoresis (2D-GE) approach allows for the expression analysis of quite a number of hepatic enzymes related to amino acid catabolism and urea cycle^16^. Therefore, the aim of this study was to investigate the molecular mechanisms related to the synthesis of urea and non-urea N-metabolites in the liver of dairy cows with high and low MUC fed two dietary crude protein levels. For this, a combination of 2D-GE-based proteomics, targeted mRNA expression, N-metabolite and ^13^C urea tracer analyses were applied.

## Materials and methods

### Animals, adaptations and grouping

The experimental procedure was evaluated by an ethical committee and approved by the State Office for Agriculture, Food Safety and Fisheries Mecklenburg-Vorpommern, Germany (LALLF 7221.3-1-052/17). All experiments were performed in accordance with relevant regulations and the reporting in the manuscript followed the ARRIVE guidelines. Non-pregnant, late-lactating Holstein–Friesian cows from two commercial, loose-housing system farms were screened for milk yield and MUC, considering the last five monthly milk testing. Animals (*N* = 36) in second to fourth lactation with comparable milk yield (HMU: 32.9 ± 1.0 kg/d; *n* = 18 and LMU: 31.5 ± 1.4 kg/d; *n* = 18) were bought in pairs of two per farm with high (HMU: 276 ± 4 mg/L; *n* = 18) and low (LMU: 186 ± 4 mg/L; *n* = 18) MUC. Each cow pair was selected from a list of six to fourteen animals provided by the farmer. On farms, each cow pair was fed twice daily the same diet for ad libitum intake. Cow pairs were clinically examined and healthy and transported to the institutional barn at FBN in 18 blocks. Each block entered the experiment at different times between September 2018 and February 2020. Farmers were informed about and agreed to the use of cows in the present study. Cow pairs were assigned alternating to an isocaloric ration (10.1 ± 0.1 MJ ME/kg DM) with normal (NP: 15.9 ± 0.1%) or low (LP: 13.8 ± 0.2%) crude protein content (Table 1), resulting in 4 groups (HMU-NP, HMU-LP, LMU-NP, and LMU-LP) of *n* = 9 cows in each group. Cows were adapted to the ration and the loose housing system for 2 weeks. They were fed at 0500 h and 1700 h for ad libitum intake and milked at 0430 h and 1630 h. The body condition score (BCS) and dorsal back fat thickness were determined once a week as previously described^17^. At the end of the second week, animals were 329 ± 13 days in milk, had a BCS of 3.5 ± 0.5 on a five-point scale, and a milk yield of 23.4 ± 0.8 kg/d.

**Table 1:** Feed constitutes, nutrient composition and energy concentration of the normal protein (NP) and low protein (LP) diets (means ± SEM).

	NP	LP
**Ingredients g/kg of DM**		
Grass silage	275 ± 14	227 ± 13
Corn silage	311 ± 3.95	369 ± 11
Triticale silage	22.2 ± 15.2	–
Forage rye silage	14.18 ± 9.69	82.5 ± 25.4
Hay	17.5 ± 7.69	–
Barley straw	3.97 ± 2.71	4.33 ± 2.97
Corn meal	52.9 ± 5.55	70.8 ± 10.5
Wheat kernels	116 ± 12.2	131 ± 6
Rapeseed extraction meal	143 ± 10.5	100 ± 7
Soybean extraction meal	2.10 ± 1.43	–
MF 2000^1^	28.5 ± 19.4	–
Mineral feed^2^	9.05 ± 0.31	10.3 ± 0.3
Limestone^3^	3.83 ± 0.08	3.72 ± 0.18
Feed salt^4^	1.46 ± 0.14	1.56 ± 0.02
**Nutrients, g/kg of DM**		
Crude ash^5^	73.8 ± 3.80	69.1 ± 0.6
Crude fat	29 ± 0.61	27.2 ± 0.7
Crude protein	159 ± 1.28	139 ± 2.1
Crude fiber	171 ± 4.52	163 ± 2.8
ADF	197 ± 3.58	189 ± 3.1
NDF	377 ± 7.12	353 ± 4.6
Starch	210 ± 6.38	248 ± 5.2
Sugar	23 ± 1.40	14.4 ± 1.6
Utilizable crude protein^6^	152 ± 1.06	146 ± 0.96
N, g/kg DM^7^	29.5 ± 0.60	25.1 ± 0.3
Ruminal N balance^8^	2.01 ± 0.30	0.76 ± 0.20
ME, MJ/kg DM	10.2 ± 0.14	10.1 ± 0.17
NEL, MJ/kg DM	6.18 ± 0.09	6.16 ± 0.12

^1^MF2000 pell. (Ceravis Produktion und Transport GmbH, Malchin, Germany): 24% crude protein, 2.6% crude fat, 5.10% crude fiber, 8% crude ash, 0.73% calcium, 0.5% phosphorus, 0.65% sodium, 7.1 MJ NEL/kg; Additives: 10,000 I.E. vitamin A, 1125 I.E. vitamin D3, 40 mg vitamin E, 0.6 mg I, 0.4 mg Co, 50 mg Mn, 75 mg Zn, 0.4 mg Se.

^2^Panto Mineral R 8609 (HL Hamburger Leistungsfutter GmbH, Hamburg, Germany): composition: 20% calcium, 6% phosphorous, 8% sodium, 6% magnesium, 0.03% inorganic nitrogen, 13.74% phosphorous pentoxide. Additives per kg original substance: 900,000 IU vitamin A, 200,000 IU vitamin D3, 4.5 g vitamin E, 1.5 g Cu, 8 g Zn, 5 g Mn, 60 mg I, 21 mg Co, 50 mg Se.

^3^Bergophor CaCO3 V001 (Hohburg Mineralfutter GmbH, Lossatal, Germany): 37% calcium.

^4^Animal feed salt (ESCO—European Salt Company GmbH & Co.KG, Hanover, Germany): 38% sodium, 0.3% calcium, 0.01% magnesium.

^5^Measured quantity elements g/kg in LP: calcium 7.0 ± 0.2, phosphorous 4.1 ± 0.1, sodium 2.3 ± 0.2, magnesium 2.3 ± 0.1, potassium 10.3 ± 0.6; NP: calcium 7.5 ± 0.4, phosphorous 4.4 ± 0.1, sodium 2.4 ± 0.2, magnesium 2.6 ± 0.1, potassium 10.5 ± 0.6.

^6^Utilizable crude protein (g/kg DM) = [11.93 – (6.82 × UDP) (g/kg DM) / crude protein (g/kg DM)] × ME (MJ/kg DM) + 1.03 × UDP (g/kg DM), with UDP = undegradable protein (GfE, 2001)^41^.

^7^ N measured in fresh feed including volatile nitrogen compounds and normalized to dry matter content.

^8^Ruminal N balance (g/kg DM) = [crude protein (g/kg DM) − utilizable crude protein (g/kg DM)]/6.25(GfE, 2001)^41^.

### Adaptation, nitrogen balance study, ^13^C urea tracer administration, and tissue sampling

The N balance study was conducted according to the animal trait of ontology ATOL_0005338 and the respectively described method^18^. Animals were transferred to a climate-controlled room at 15 °C and adapted to a tie-stall on a 2.43 × 1.56 m stanchion for one week. Each cow pair was continuously fed ad libitum from one batch, which was aliquoted in vacuumed 40 kg-plastic bags, and stored at + 4 °C. A feed sample was taken and frozen at − 20 °C. On the last day of the adaptation period, cows were prepared for a 4-d N balance study by fitting the cows with a urinal consisting of a customized synthetic leather model which had a cutout around the vulva area (Supplemental Figure 1). The urinal was glued to the cow below the anus around the vulva and the cutout was covered with a sewn-on piece of synthetic leather.

On the first to fourth day of the balance trial, animals were continuously fed from the same batch but only 97% of the individual daily ad libitum intake to ensure homogenous intake. Between 0600 and 2000 h, feed was provided in 7 equally-sized portions in 2-h intervals amounting to 58% of the daily ad libitum intake. The remaining 39% were given at 2000 h in one portion. On the first day of the balance study, a flexible plastic tube (4.5 cm, inner diameter) was connected to the urinal and a 30 L-container, and urine was collected for 24 h to measure the weight and volume and from these to calculate the density of the urine. Samples of urine (two 15-mL aliquots) were taken and stored at − 20 °C until analysis. On the second day of the balance study and onwards, the container was prefilled with 400 mL (564 g) of 50% sulfuric acid and placed on a shaker or magnetic stirrer to minimize volatile N losses. Feces were quantitatively collected several times a day, combined and stored in a container at 4 °C. The 24-h collections were weighed and homogenized by stirring, and subsamples of feces (about 300 g) were taken and stored at − 20 °C. Animals were milked twice daily (0630 h and 1830 h), milk yield was recorded, and two 15-mL aliquots from each milking were pooled on a daily basis and stored at − 20 °C until analysis.

On the first day of the N balance study, cows were equipped with a jugular vein catheter, which was flushed with 20 mL 0.9% saline and 10 mL 3.5% sodium citrate. On the third day, a ^13^C urea-tracer (^13^C urea, ≥ 99 atom%; Sigma-Aldrich, St. Louis, USA) was injected as a bolus (2 g per 650 kg body weight (BW), dissolved in saline), and the catheter was flushed with 20 mL saline. Blood samples were collected at 5, 10, 20, 30, 60, 120, 180, 240, 360, 480, 600 and 1320 min after bolus injection in 9-mL EDTA-containing tubes, immediately placed on ice, centrifuged (1345×*g*, 20 min, 4 °C), and stored at − 20 °C as described previously^9^.

The day after the balance study, animals were transferred to the institute's slaughterhouse. Animals were anesthetized by a captive bolt stunning and exsanguinated. A blood sample was collected in an EDTA-containing tube and stored on ice. The blood was centrifuged and the obtained plasma was frozen at − 80 °C. The liver was weighed and a tissue sample was taken from the middle of the left lobe. The tissue sample was immediately chilled on ice, cut into smaller pieces, shock-frozen in liquid N_2_ and stored at − 80 °C until further analysis.

### Feed, milk, urine, feces, and plasma analyses

For the determination of feed dry matter, samples were air dried at 60 °C for 24 h, ground and dried again at 105 °C for 4 h. For N measurement, frozen fresh feed samples were ground with the addition of dry ice. The drinking water was analyzed for total N concentration (0.58 mg/L) by the method of the German Institute for Standardization. According to the 24-h excretion volumes, proportional pool samples of feces, milk, and acidified urine were prepared and sent to the accredited laboratory Landwirtschaftliche Untersuchungs- und Forschungsanstalt der LMS Agrarberatung GmbH (LUFA, Rostock, Germany) for analysis of total N using the Kjeldahl method for feces and feed, and a "vario MAX" element analyzer (Elementar; Langenselbold, Germany) according to Dumas for milk and acidified urine.

Frozen milk samples of the 4-day balance study were thawed and centrifuged for 10 min at 4 °C and 50,000×*g* to remove the milk fat. The resulting skim milk was subjected to urea concentration analysis using ABX Pentra C400 analyzer (HORIBA Europe GmbH, Oberursel, Germany) and the kit: LT-UR0010 (Labor + Technik Eberhard Lehmann GmbH, Germany). Urea concentration measured in skim milk was recalculated for whole-milk and a mean value of 4 days was calculated. The plasma sample obtained 10 min before tracer administration was subjected to the analysis of aspartate amino transferase (AST), GLDH, and gamma-glutamyltransferase (γ-GT) using the following kits: AST: A11A01629 (HORIBA Europe GmbH), GLDH: LT-GD 0010 (Labor + Technik Eberhard Lehmann GmbH), and γ-GT: AX0N00016 (Axon Lab AG, Germany). The analysis of plasma free amino acids was performed as described previously^19^ using a HPLC with fluorescence detection (HPLC 1200/1260 infinity II series; Agilent Technologies) with the following modifications. Amino acids were separated on a Gemini 250 × 4.6 mm 5 µm ODS (C18) 110 Å column using a phosphate buffer (pH 7.45): ACN/methanol/water (45:45:10) gradient from 7 to 100%. Urea concentrations in plasma samples obtained from slaughter were measured using again the LT-UR0010 kit, and urea concentrations from 50-fold diluted acidified urine samples were analysed by HPLC (1200/1260 infinity II Series; Agilent) with a 300 × 7.8 mm Rezex RCM-Monosaccharide column (Phenomenex Inc.) as described previously^9^. Non-urea N-metabolite concentrations in plasma obtained during slaughter and in non-esterified urine samples were analyzed by HPLC on a 250 × 4.6 mm Synergi 4 µm Hydro-RP 80 Å column protected by a corresponding 4 × 3 mm pre-column (both Phenomenex Inc., Aschaffenburg, Germany) as described before^9^.

### ^13^C urea analysis

The plasma ^13^C urea enrichment analysis was performed as previously described^9^. Briefly, the urea was derivatized and the t-butyldimethylsilyl derivate was analyzed on a gas chromatograph-mass spectrometer (GC–MS, QP 2010, coupled with GC 2010, AOC-20i; Shimadzu, Duisburg, Germany). Detection occurred at m/z 231 and 232. A two-exponential curve fitting to ^13^C urea enrichment was performed and mole % excess (MPE) was as: MPE (t) = a × e^(−b × t)^ + c × e^(−d × t)^ . The variables a, b, c and d were used to calculate the area under the curve (AUC) AUC = a/b + c/d and the mean residence time MRT (h) = (a/b^2^ + c/d^2^)/(a/b + c/d). Based on the tracer dosage (D) in mg, the urea pool size (Q) was calculated according to^20^: Q (mg/kg BW) = (D × MRT)/(AUC × BW).

### Proteome analysis

Frozen liver tissue was ground to fine powder using a mortar and pestle under liquid N_2_. For protein extraction, 50 mg tissue powder and 200 µL lysis buffer consisting of Triton X-100 (1% v/v), sodium deoxycholate (DOC; 0.5% v/v), sodium dodecyl sulfate (SDS; 0.1% w/v), 8 mM NaCl, 2.7 mM KCl, 6.9 mM Na_2_HPO_4_, 1.5 mM KH_2_PO_4_, 100 µL PhosSTOP (Roche) and 10 µL Protease Inhibitor Cocktail (AppliChem) were placed in the shaker for 45 s. Samples were centrifuged for 20 min at 4 °C and 13,000×*g*. The protein concentration was measured using the bicinchonin acid method and bovine serum albumin as standard. For each animal, two 2D-gels were prepared resulting in a total of 72 gels (pages 8 to 80 of the Supplementary file). For each gel, 300 µg protein extract was mixed with rehydration buffer (8 M urea, 2% CHAPS, 0.5% IPG strip buffer, 15 mM dithiothreitol (DTT) and 0.002% bromophenol blue to a final volume of 450 µL, and applied to 24 cm-IPG BlueStrip (Serva Electrophoresis) with a pH gradient of 3–10. The strips were covered with 1 mL of mineral oil and placed in the Ettan IPGphor3 (GE Health-care, Munich, Germany) for isoelectric focusing (IEF). Active rehydration and IEF was performed at 50 µA/strip at 50 V for 8 h, 500 V for 1 h, 1000 V for 2 h, and 8000 V for 8 h 40 min. The IPGs were first equilibrated in a buffer of 75 mM Tris–HCl (pH 8.8), 29.3% glycerol, 2% SDS, 6 M urea, 0.002% bromophenol blue, and 1% DTT for 15 min and then agitated again for 15 min with the same buffer, but instead of DTT with 2.5% iodoacetamide (IAA). The IPG strips were transferred to 12.5% polyacrylamide gels (255 × 200 × 0.65 mm; 2D HPE Large Gel NF, Serva Electrophoresis) and the 2D-GE was performed on HPE BlueHorizon tower (Serva Electrophoresis) at 1 W for 30 min, 3 W for 30 min, 5 W for 10 min, 30 W for 3 h 50 min and 40 W for 50 min. The SDS-PAGE gels were stained overnight in colloidal coomassie and then de-stained in 25% methanol. The gels were scanned and the images saved as tiff format. Individual gels were warped to one master gel image to allow for image analysis using the software DELTA2D (version 4.6, DECODON, Greifswald, Germany; http://www.decodon.com). Spot volumes significantly different between groups or diets were stamped out of the gel with a spot cutter (Ø 2 mm) and transferred to a 96-well micro titer plate. Protein identification was performed as previously described^19^. Briefly, proteins were digested using trypsin and the molecular masses of the tryptic digest were measured using a 5800 MALDI TOF/TOF Analyzer (Applied Biosystems, Forster City, CA, USA). The spectra were registered in a mass range of 900 to 3700 Da with a focus on 2000 Da. An automatic internal calibration was performed as a two-point calibration when the peptides with the monoisotopic (M + H)^+^ m/z at 1045,556 and 2211,104 reached a signal-to-noise (S/N) ratio of at least 20. Peak lists were generated using the "peak-to-maskot" script of the 4000 Series Explorer™ software (V3.5). Peak lists were generated for a S/N ratio of 10, a peak density of 15 peaks per 200 Da, a minimum peak area of 100 and a maximum of 60 peaks per spot. The results of the MALDI-TOF-MS analysis were verified with TOF-TOF measurements and performed for the three highest peaks of a TOF spectrum. Internal calibration was performed as a one-point calibration using either the mono-isotope arginine (M + H)^+^ m/z at 175.119 or lysine (M + H)^+^ m/z at 147.107, if a S/N ratio of at least 5 was obtained. Peak lists were generated according to the above script with the following settings: Mass range 60 to precursor − 20 Da, peak density of 15 peaks per 200 Da, minimum area of 100 and maximum 65 peaks per precursor. The peak list was created for a S/N ratio of 7. For the identification of the proteins, data base search with peptide mass fingerprint (PMF) was performed against the NCBInr (National Center for Biontechnology Information, http://www.ncbi.nlm.nih.gov/) database using the Mascot search engine version 2.4.1 (Matrix Science, London, UK). Search parameters were: taxonomy: “all entries”; variable modifications: “carbamidomethyl (C)” and “oxidation (M)”; precursor tolerance ± 50 ppm; peptide charge “1+”; MS/MS fragment tolerance “0.5 Da”; “monoisotopic”. Mass spectrometry data were deposited to the ProteomeXchange Consortium via the PRIDE^21^. The gene names of the identified proteins with a protein score greater than 64 (*P* < 0.05) were imported into ClueGO software^22^ for analysis of activated pathways. The results were visualized using Cytoscape software version 3.8.0^23^.

### mRNA analysis

The extraction of RNA from 20 mg liver tissue powder was performed using the innuPREP RNA Mini Kit 2.0 and residual DNA was removed using the innuPREP Dnase I Digest Kit (both Analytik Jena AG, Jena, Germany). The RNA concentrations were determined on a NanoPhotometer (Implen GmbH, Munich, Germany). The RNA quality was evaluated by analyzing the RNA integrity number on a 2100 Bioanalyzer (Agilent), which resulted in values between 7.0 and 7.5. The cDNA synthesis (1000 ng total RNA) was performed using SensiFAST cDNA Synthesis Kit (Bioline, London, UK) and the obtained cDNA frozen at − 80 °C until use. Real-time qPCR was performed in duplicates using 2 µL diluted cDNA (10 ng/µL), 3 µL nuclease-free water, 0.5 µL of each primer (4 pmol/µL) listed in Supplemental Table 1 and 6 µL 2 × buffer SensiFAST SYBR No-ROX mix (Bioline) for one PCR reaction. The protocol was as follows: 60 s at 95 °C followed by 40 cycles of 5 s at 95 °C, 10 s at 60 °C and 5 s at 72 °C using a LightCycler 2.0 (Roche). The efficiency of amplification was calculated using LinRegPCR software, version 2014.4 (Academic Medical Centre, Amsterdam, Netherlands). Amplicons were sequenced on an ABI 3130 Genetic Analyzer (Life Technologies GmbH, Darmstadt, Germany) to confirm sequence identity.

### Statistical analysis

The experimental design bases on a 2-factoral variance analysis including MUC and CP content as factors. Based on this model, the minimum number of animals (n) in each group was calculated choosing type-I error (α = 0.05), type-II error (β = 0.2), and residual variance = 1. If aimed for a residual standard deviation equal to 1, n = 9 animals per group were required. Due to a technical problem with the climate control resulting in too low feed intake in one block, data from two animals fed NP diet were excluded from the statistical analysis. Thus, 8 HMU-NP, 9 HMU-LP, 8 LMU-NP, and 9 LMU-LP cows were considered for statistical analysis using SAS software (version 9.4, SAS Institute Inc., Cary, NC, USA). For the balance trial, means of the 4 daily collections were calculated. Data were analyzed with measurement analyses of variance using the MIXED procedure. The ANOVA model included the fixed factors MUC (levels: HMU/LMU), diet (levels: NP/LP), and the interaction of MUC × diet. Least-square means (LSM) and their standard error (SE) were computed for each fixed effect. The slice statement of the MIXED procedure was used to perform a partitioned analysis of the LSM for the interaction of group of milk urea concentration × diet, and pairwise differences were tested by using the Tukey–Kramer procedure. Results with a *P* value < 0.05 were considered significant and 0.05 < *P* < 0.1 as trend.

## Results

### Animal characteristics and nitrogen balance

Cows with divergent MUC were comparable in DIM, parity, BW, BCS, back fat thickness, dry matter, energy and water intake, milk yield, and ECM, irrespective of the diet (Table 2). Milk fat content was significantly higher in HMU than LMU cows (*P* = 0.020), and the fat to protein ratio tended to be higher in the HMU than LMU group (*P* = 0.076). However, milk protein and lactose contents were not different between groups or diets. Nitrogen intake was comparable between groups, but cows tended to ingest 27 to 38 g N per day more on the NP than LP diet (*P* = 0.094; Table 2). However, the daily N output in milk and feces was not affected by CP content. In contrast, daily urinary N excretion was higher on the NP than LP diet (*P* = 0.002), but did not differ between cow groups. According to the experimental design, HMU cows had higher MUC (*P* = 0.004), and tended to secrete also more urea in milk per day (*P* = 0.095). Daily urinary urea excretion was on average 37% higher on the NP relative to LP diet (*P* < 0.001), but was not different between HMU and LMU cows. Furthermore, N balance and daily urinary creatine, creatinine, hippuric acid and uric acid excretions were not affected by CP content or cow group.

**Table 2:** Animal characteristics, nitrogen intake and excretion of dairy cows with high (HMU) and low milk urea concentration (LMU) fed a normal (NP) and a low dietary crude protein level (LP) under conditions of interval feeding (97% of ad libitum intake).

Parameter	NP				LP				*P* value^1^		
	HMU	LMU	SE	*P* value^2^	HMU	LMU	SE	*P* value^2^	Diet	Group	Diet × group
DIM	347	313	28	0.399	328	330	27	0.958	0.977	0.562	0.515
Parity	2.5	3	0.28	0.209	3	2.6	0.26	0.236	0.918	0.918	0.088
BW, kg	653	693	25	0.137	702	716	23	0.892	0.588	0.425	0.555
BCS	3.5	3.8	0.2	0.376	3.6	3.5	0.2	0.791	0.813	0.642	0.409
Backfat, mm	14	17	2	0.340	16	13	2	0.311	0.683	0.999	0.168
DMI, kg/d	16.4	15.8	0.7	0.545	17.0	17.1	0.7	0.924	0.189	0.707	0.613
MEI, MJ/d	167	161	9	0.623	172	174	9	0.904	0.304	0.783	0.660
Water intake, L/d	62	60	4	0.724	58	64	4	0.260	0.958	0.601	0.303
Milk yield, L/d	22.2	20.8	1.7	0.564	22.8	23.4	1.6	0.773	0.329	0.824	0.537
ECM, kg/d	26.4	22.4	1.8	0.130	25.3	25.3	1.7	0.993	0.621	0.268	0.263
Milk fat, %	5.0^a^	4.2^b^	0.2	0.016	4.6^ab^	4.3^ab^	0.2	0.390	0.440	0.020	0.214
Milk protein, %	4.0	3.7	0.1	0.196	3.6	3.7	0.1	0.932	0.201	0.374	0.316
Milk lactose, %	4.7	4.6	0.1	0.485	4.7	4.8	0.1	0.657	0.237	0.838	0.418
FPR	1.3	1.1	0.1	0.144	1.3	1.2	0.1	0.284	0.746	0.076	0.735
**Total N measures**											
N-water intake, mg/d	35.9	34.7	2.5	0.724	33.5	37.4	2.4	0.260	0.958	0.601	0.303
N-feed and water intake, g/d	486	467	23	0.555	437	440	21	0.933	0.094	0.709	0.626
N-milk, g/d	142	123	10	0.191	142	143	9	0.916	0.288	0.374	0.304
N-feces, g/d	159	150	10	0.512	151	150	9	0.954	0.698	0.605	0.661
N-urine, g/d	146^ac^	148^ab^	9	0.861	115^bd^	120^cd^	8	0.712	0.002	0.704	0.900
N-balance, g/d	39.9	45.9	20	0.831	29.1	26.6	19	0.926	0.440	0.927	0.826
**Milk N metabolites**											
Urea, g/d	8.44	6.37	0.79	0.076	6.41	5.81	0.75	0.577	0.105	0.095	0.350
Urea, g/L	0.37^a^	0.31^ab^	0.03	0.074	0.28^b^	0.25^ab^	0.02	0.291	0.004	0.046	0.547
**Urine N metabolites**											
Urea, g/d	244^ac^	247^ab^	18	0.903	182^bd^	176^cd^	17	0.791	< 0.001	0.926	0.787
Hippuric acid, g/d	113	93.2	12	0.245	109	119	11	0.544	0.351	0.647	0.207
Creatinine, g/d	13.2	13.5	0.8	0.771	12.7	13.0	0.8	0.807	0.565	0.705	0.965
Creatine, g/d	9.9	10.7	1	0.582	9.8	11.6	1	0.192	0.724	0.197	0.614
Uric acid, g/d	5.2	6.3	0.8	0.323	6.4	7.1	0.7	0.477	0.227	0.230	0.815

Data are given as least square means and standard error (SE).

^a-d^Different superscript letters within one row indicate *P* < 0.05 (Tukey-test).

DIM: days in milk; DMI: dry matter intake; MEI: metabolizable energy intake; ECM: energy corrected milk; FPR: milk fat% -to-milk protein%-ratio.

^1^*P* value from ANOVA analysis.

^2^*P* value from Tukey slice test.

### Plasma metabolites and urea pool

Among plasma amino acids, citrulline concentrations were 19% higher in HMU than in LMU animals (*P* = 0.019) on either diet (Table 3). Plasma glycine and serine concentrations were (*P* < 0.05), and asparagine, tyrosine and alanine concentrations tended (*P* < 0.1) to be 11 to 23% greater in HMU than LMU cows on the NP but not LP diet. Furthermore, plasma glutamine and arginine concentrations increased in HMU animals with increasing CP content (*P* < 0.05), but this increase was not observed in LMU animals. In addition, the plasma concentrations of ornithine were not different between groups or diets. The plasma AST activity was 24% higher on the LP than NP diet (*P* = 0.029), while GLDH and γ-GT did not differ between diets or groups. Plasma creatine and creatinine, concentrations were not different between groups and diets, but LMU cows had significantly higher creatine concentrations on the LP than NP diet (*P* = 0.045; group × diet interaction: *P* = 0.032). Plasma uric acid and hippuric acid concentrations were in average 8 to 9% and 18 to 28%, respectively, higher in HMU compared to LMU animals (*P* = 0.020), but not affected by diet (Table 3). Plasma urea concentrations were 27% and the urea pool 19% higher on the NP than LP diet (*P* < 0.01), and both variables were on average 16% higher in HMU than LMU cows (*P* < 0.05).

**Table 3:** Plasma N-metabolite concentrations and urea pool size of dairy cows with low (LMU) and high milk urea concentration (HMU) fed normal (NP) and low (LP) dietary crude protein level under conditions 97% of ad libitum feeding.

Parameter	NP			LP			*P* value^2^		
	HMU	LMU	*P* value^3^	HMU	LMU	*P* value^3^	Diet	Group	Diet × Group
Citrulline, μmol/L	85.5 ± 5.7^ab^	73.6 ± 6.0^ab^	0.162	84.2 ± 5.3^a^	68.1 ± 5.3^b^	0.042	0.546	0.019	0.714
Glycine, μmol/L	332.3 ± 17.4^a^	273.0 ± 18.6^b^	0.027	284.2 ± 16.4^ab^	322.6 ± 16.4^ab^	0.109	0.964	0.547	< 0.01
Serine, μmol/L	85.2 ± 5.1^a^	67.4 ± 5.5^b^	0.024	72.9 ± 4.8^ab^	79.6 ± 4.8^ab^	0.331	0.999	0.282	0.022
Asparagine, μmol/L	48.9 ± 4.0	37.1 ± 4.3	0.055	38.6 ± 3.8	43.0 ± 3.8	0.418	0.583	0.363	0.051
Glutamine, μmol/L	335 ± 18.7^a^	290.4 ± 20.0^ab^	0.114	280.6 ± 17.7^b^	286.1 ± 17.7^ab^	0.827	0.125	0.300	0.187
Arginine, μmol/L	93.6 ± 6.3^a^	79.7 ± 6.8^ab^	0.146	75.1 ± 6.0^b^	78.6 ± 6.0^ab^	0.678	0.129	0.417	0.176
Tyrosine, μmol/L	44.3 ± 3.9	33.9 ± 4.2	0.054	39.5 ± 3.7	38.5 ± 3.7	0.843	0.920	0.116	0.187
Aspartate, μmol/L	5.48 ± 0.45^a^	4.39 ± 0.48^ab^	0.113	4.21 ± 0.43^b^	4.51 ± 0.43^ab^	0.620	0.211	0.391	0.133
Alanine, μmol/L	271 ± 15.7	227.8 ± 16.8	0.070	264.4 ± 14.8	262.5 ± 14.8	0.926	0.375	0.158	0.196
Ornithine, μmol/L	40.2 ± 4.2	42.5 ± 4.5	0.717	35.5 ± 4.0	34.3 ± 4.0	0.831	0.137	0.901	0.680
AST^1^, U/L	72.1 ± 7	63.2 ± 7	0.398	84.8 ± 7	83.0 ± 7	0.856	0.029	0.459	0.622
Allantoine, μmol/L	570.5 ± 30.7	523.4 ± 32.8	0.303	539.9 ± 29	514.5 ± 29	0.540	0.521	0.243	0.723
Creatine, μmol/L	330 ± 14.3^ab^	286 ± 15.3^b^	0.045	315.3 ± 13.5^ab^	335.3 ± 13.5^a^	0.305	0.233	0.403	0.032
Creatinine, μmol/L	24.8 ± 2.5	22.5 ± 2.7	0.539	26.8 ± 2.4	23.3 ± 2.4	0.315	0.581	0.260	0.821
Uric acid, μmol/L	76.4 ± 3.1	70.4 ± 3.4	0.204	82.1 ± 3.0	73.6 ± 3.0	0.053	0.165	0.028	0.693
Hippuric acid, μmol/L	78.2 ± 6.3	61.0 ± 6.7	0.072	86.0 ± 5.9	72.4 ± 5.9	0.117	0.132	0.020	0.774
Urea, μmol/L	5831 ± 344^ab^	5100 ± 368^ac^	0.028	4647 ± 324^ cd^	3942 ± 324^bd^	0.365	< 0.01	0.044	0.969
Urea pool, mg/kg BW	156.6 ± 9.3^a^	129.8 ± 9.3^ab^	0.052	123.8 ± 8.8^b^	107.0 ± 9.3^ab^	0.199	< 0.01	0.025	0.593

Data are given as least square means and standard error (SE).

^a–c^Different superscript letters within one row indicate *P* < 0.05 (Tukey test).

^1^AST, aspartat-aminotransferase.

^2^*P* value from ANOVA analysis.

^3^*P* value from Tukey slice test.

### Liver proteome

The 2D-image analysis revealed a total of 808 spots commonly occurring on each gel. The statistical analysis yielded 199 spots, which differed between groups, diets, or the group × diet interaction by *P* < 0.1. The protein identification of these spots revealed 56 spots differentially expressed between HMU and LMU cows (34 spots at *P* < 0.05; 22 spots at 0.05 < *P* < 0.1), and 72 spots differentially expressed between LP and NP diets (37 spots at *P* < 0.05; 35 spots at 0.05 < *P* < 0.1) (Supplemental Figure 2a, b; Supplemental Table 2).

**Figure 1:**
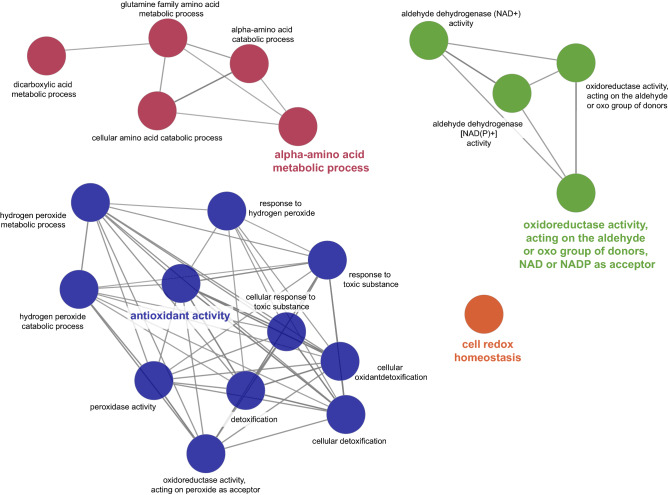
Functional classification of differentially (*P* < 0.05) expressed proteins in the liver of HMU and LMU cows based on Gene ontology biological processes using ClueGO software (version 3.8.0; Cytoscape Software; https://cytoscape.org).

The ClueGO analysis considered 34 spots including 28 different proteins (19 proteins at *P* < 0.05; 9 proteins at 0.05 < *P* < 0.1) for cow group comparison, and 38 spots including 29 different proteins (18 proteins at *P* < 0.05; 11 proteins at 0.05 < *P* < 0.1) for ration comparison. For the MUC comparison, ClueGO analysis yielded 4 biological processes: (1) alpha-amino acid metabolic processes, (2) oxidoreductase activity, (3) antioxidant activity; and (4) cell redox homeostasis (Fig. 1). In detail, alpha-amino acid metabolic processes were characterized by a lower expression of dimethylarginine dimethylaminohydrolase 1 (DDAH1), malate dehydrogenase 2 (MDH2), nitrilase family member 2 (NIT2) and 10-formyltetrahydrofolate dehydrogenase (ALDH1L1), and a higher expression of GLDH, glycine N-acyltransferase (GLYAT) and peroxiredoxin 3 (PRDX3) in HMU cows (Table 4). In addition, methylmalonate semialdehyde dehydrogenase (ALDH6A1) and thiosulfate sulfurtransferase (TST) occurred each in 2 spots, each one down- and one upregulated in HMU cows. Among the proteins with oxidoreductase activity, the 4-trimethylaminobutyraldehyde dehydrogenase (ALDH9A1), involved in carnitine biosynthesis, and glyceraldehyde-3-phosphate dehydrogenase (GAPDH) were higher expressed, whereas retinal dehydrogenase 1 (ALD1A1) was lower expressed in HMU cows. Catalase (CAT) and peroxiredoxin 6 (PRDX6), both involved in oxidative stress defence, were downregulated but protein disulfide isomerase family A member 3 (PDIA3), superoxide dismutase 1 (SOD1) and the fatty acid binding protein 1 (FABP1) upregulated in HMU cows.

**Table 4:** ClueGO analysis of the liver proteome of dairy cows with high (HMU) and low milk urea concentration (LMU) fed a normal (NP) and a low dietary crude protein level (LP).

Gel spot No	Gene name	Description	Mean rel. abundance (Vol %)	Mean rel. abundance (Vol %)	Fold change	*P* value
MUC			LMU	HMU		Tukey
683	PRDX6	Peroxiredoxin 6	0.025	0.020	1.27	0.049
203	MDH2	Malate dehydrogenase 2	0.071	0.058	1.22	0.035
515	NIT2	Nitrilase Family Member 2	0.032	0.027	1.21	0.006
216	TST	Thiosulfate sulfurtransferase	0.066	0.055	1.20	0.044
621	DDAH1	Dimethylarginine dimethylaminohydrolase 1	0.176	0.150	1.17	0.023
644	CAT	Catalase	0.138	0.118	1.17	0.042
206	ALDH1L1	10-formytetrahydrofolate dehydrogenase	0.049	0.044	1.11	0.014
655	ALDH6A1	Methylmalonate semialdehyde dehydrogenase	0.304	0.283	1.07	0.028
582	ALD1A1	Retinaldehyde dehydrogenase 1	0.075	0.072	1.04	0.087
509	GLDH	Glutamate dehydrogenase (NAD(P)( +))	0.375	0.419	− 1.12	0.027
225	GLYAT	Glycine N-acyltransferase	0.228	0.236	− 1.04	0.019
188	GAPDH	Glyceraldehyde-3-Phosphate dehydrogenase	0.190	0.200	− 1.05	0.090
580	PDIA3	Protein disulfide isomerase A3	0.030	0.032	− 1.07	0.090
771	FABP1	Fatty acid binding protein 1	1.049	1.129	− 1.08	0.015
507	ALDH6A1	Methylmalonate semialdehyde dehydrogenase	0.802	0.877	− 1.09	0.012
676	PRDX3	Peroxiredoxin 3	0.128	0.140	− 1.10	0.031
328	SOD1	Superoxide dismutase 1	0.209	0.245	− 1.17	0.018
713	TST	Thiosulfate sulfurtransferase	0.044	0.055	− 1.27	0.011
377	ALDH9A1	4-trimethylaminobutyraldehyde dehydrogenase	0.022	0.030	− 1.36	0.009
Ration			LP	NP		
697	ALDH6A1	Methylmalonate semialdehyde dehydrogenase	0.012	0.009	1.25	0.097
144	ACAA2	Acetyl-CoA acyltransferase 2	0.254	0.213	1.19	0.012
442	GLYAT	Glycine N-acyltransferase	0.263	0.228	1.15	0.072
639	ACO2	Aconitate hydratase	0.072	0.063	1.15	0.072
499	ACADM	Acyl-CoA dehydrogenase medium chain	0.299	0.263	1.14	0.010
112	SHMT1	Serine hydroxymethyltransferase 1	0.072	0.063	1.14	0.042
448	SORD	Sorbitol dehydrogenase	0.417	0.374	1.11	0.023
538	ECHS1	Enoyl-CoA hydratase short chain 1	0.279	0.252	1.11	0.056
232	ALDH1L1	10-formyltetrahydrofolate dehydrogenase	0.196	0.178	1.10	0.023
137	ASS1	Argininosuccinate synthase 1	0.558	0.512	1.09	0.069
200	GPD1	Glycerol-3-Phosphate dehydrogenase 1	0.115	0.106	1.09	0.026
234	ECHS1	Enoyl-CoA hydratase short chain 1	0.225	0.207	1.09	0.082
655	ALDH6A1	Methylmalonate semialdehyde dehydrogenase	0.300	0.286	1.05	0.045
270	HOGA1	4-hydroxy-2-oxoglutarate aldolase	0.065	0.068	− 1.04	0.081
183	GAPDH	Glyceraldehyde-3-Phosphate dehydrogenase	0.089	0.094	− 1.05	0.091
321	NME2	Nucleoside diphosphate kinase 2	0.057	0.064	− 1.12	0.022
627	P4HB	Protein disulfide-isomerase	0.131	0.148	− 1.13	0.099
127	P4HB	Protein disulfide-isomerase	0.061	0.070	− 1.14	0.083
206	ALDH1L1	10-formyltetrahydrofolate dehydrogenase	0.042	0.051	− 1.23	0.002
719	ALDH6A1	Methylmalonate semialdehyde dehydrogenase	0.025	0.032	− 1.26	0.059
692	ECHS1	Enoyl-CoA hydratase short chain 1	0.031	0.040	− 1.28	0.059

**Figure 2:**
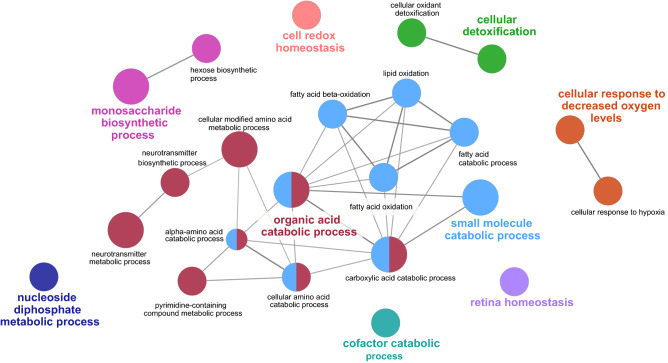
Functional classification of differentially (*P* < 0.05) expressed proteins in the liver after feeding a NP or LP ration based on Gene ontology biological processes using ClueGO software (version 3.8.0; Cytoscape Software; https://cytoscape.org).

Comparing the effect of diet, ClueGO analysis identified 8 groups of biological processes, the major related to organic acid and small molecule catabolic process, monosaccharide biosynthetic processes, cellular responses to decreased oxygen levels, and cellular detoxification (Fig. 2). Among organic acid and small molecule catabolic processes, ASS1, involved in arginine biosynthesis, serine hydroxymethyltransferase 1 (SHMT1), involved in serine and glycine biosynthesis, medium chain specific acyl-CoA dehydrogenase (ACADM), which is involved in identical protein binding and fatty acid beta-oxidation, 3-ketoacyl-CoA thiolase (ACAA2), which catalyzes the last step of the mitochondrial β-oxidation, and aconitate hydratase (ACO2), which catalyzes the interconversion of citrate to isocitrate in the TCA cycle were all upregulated when feeding the LP ration (Table 4). In contrast, 4-hydroxy-2-oxoglutarate aldolase (HOGA1), involved in the degradation of 4-hydroxyproline, nucleoside diphosphate kinase 2 (NME2), involved in pyrimidine de novo biosynthesis, were downregulated with LP feeding. Sorbitol dehydrogenase (SORD), and glycerol-3-phosphate dehydrogenase 1 (GPD1), all of which are related to monosaccharide biosynthesis, were higher abundant with LP feeding. Furthermore, multiple spots for enoyl-CoA hydratase short chain 1 (ECHS1), protein disulphide-isomerase (P4HB), ALDH6A1, ALDH9A1, ALDH1L1 were found up and downregulated with LP feeding. Interestingly, despite ASS1 we found no further spots related to the urea cycle, creatine synthesis or purine degradation that were differentially expressed between groups or diets.

### mRNA expression

The mRNA expression analysis of key enzymes related to urea cycle, creatine and purine catabolism revealed that xanthine dehydrogenase (*XDH*) is 22% lower expressed in HMU than LMU cows when fed the NP diet (*P* = 0.043, Table 5). Furthermore, HMU cows tended to have 41% lower *GATM* and 22% lower *GAMT* mRNA expression compared to LMU cows on the NP diet (*P* < 0.1). In LMU cows, *ARG1* mRNA expression tended to be 28% lower on the LP compared to the NP diet (*P* < 0.1); however, this difference was not observed for HMU cows. Furthermore, *NOS2* and *UOX* mRNA levels were comparable between groups and diets.

**Table 5:** Hepatic mRNA expressions of key enzymes involved in urea cycling, creatine and purine catabolism. Data are given as least square means and standard error (SE).

Parameter	NP				LP				*P* value^1^		
	HMU	LMU	SE	*P* value^2^	HMU	LMU	SE	*P* value^2^	Diet	Group	Diet × Group
XDH	0.896^b^	1.205^a^	0.097	0.043	0.964^ab^	1.073^ab^	0.103	0.436	0.756	0.046	0.328
GAMT	0.909	1.114	0.075	0.063	1.049	0.954	0.071	0.347	0.892	0.461	0.048
GATM	0.866	1.221	0.128	0.075	1.055	1.040	0.136	0.934	0.977	0.209	0.172
ARG1	1.004	1.145*	0.100	0.328	1.078	0.895*	0.094	0.180	0.373	0.829	0.107
NOS2	1.060	0.931	0.136	0.510	1.112	1.116	0.128	0.983	0.380	0.642	0.621
UOX	1.091	1.119	0.139	0.889	1.044	1.030	0.139	0.939	0.617	0.961	0.877

^a,b^Different superscript letters within one row indicate *P* < 0.05 (Tukey-test).

*Same symbol within one row indicate *P* < 0.1 (Tukey-test).

XDH, xanthine dehydrogenase; GAMT, guanidinoacetate N-methyltransferase; GATM, glycine amidinotransferase; ARG1, arginase 1; NOS2, nitric oxide synthase; UOX, urate oxidase.

^1^*P* value from ANOVA analysis.

^2^*P *value from Tukey slice test.

## Discussion

### MUC comparisons

Ammonia produced in the rumen is transported to the liver where it integrates to carbamoylphosphate, which in turn reacts with ornithine forming citrulline. It has been shown earlier that HMU cows tended to have higher rumen NH_3_ concentrations^9^. A greater absorption of ruminal NH_3_/NH_4_^+^ would result in a higher production of citrulline as it is reflected by the higher plasma citrulline concentrations of HMU cows. Moreover, increased hepatic NH_4_^+^ concentrations may also be a result of a higher GLDH activity, an enzyme involved in the transamination of extrahepatic amino acids. The higher GLDH protein abundance supports the assumption of a higher NH_3_/NH_4_^+^ load in the liver of HMU cows. However, we cannot entirely exclude that increased plasma citrulline concentrations originate from the metabolism of other organs such as the glutamine metabolism in the intestine^10^.

In the urea cycle, citrulline reacts with aspartate forming argininosuccinate, which in turn is rapidly converted to arginine. Despite the higher plasma citrulline concentrations, plasma aspartate and arginine concentrations did not differ between groups. This result can be explained by the fact that arginine is not only converted to ornithine by ARG1 but also to citrulline by NOS. An increased NOS activity would thus increase citrulline concentrations. However, we found no differences in *NOS2* at least not at the mRNA expression level between groups. Nevertheless, the regulation of the enzyme activity could also be on the post-transcriptional or substrate level. Because of these speculations, future studies are required to determine the hepatic NOS activity in cows with divergent MUC.

Although plasma arginine and ornithine concentrations are comparable between groups, HMU cows had higher plasma urea concentrations and a greater urea pool size. These results correspond to earlier findings^9^. However, we found no differences between phenotypes regarding the hepatic *ARG1* mRNA expression or the protein expression of any enzyme of the urea cycle. The latter is not due to failure in the detection of these proteins, because all hepatic urea cycle enzymes are present in the 2D-map as reported in our previous studies^16,24^. Therefore, we conclude that the differences in plasma urea concentrations between groups are not due to an altered hepatic urea synthesis rate. Yet, it should be noted that we did not analyze the activity of the enzymes, which could influence plasma urea concentrations independent of their expression. For example, urea cycle activity is, among others, influenced by the concentration of arginine^25,26^. Hence, comparable plasma arginine concentrations further support our conclusion about a comparable urea cycle activity between HMU and LMU cows.

The question then arises what would be the reason for the higher plasma urea concentration of HMU cows. One contributing factor is the lower abundance of various ureolytic bacteria in the rumen such as *Succinivibrionaceae* and *Ruminococcaceae*^27^. Another reason is that HMU cows have a worse renal urea clearance rate than LMU cows, which results in higher plasma urea concentrations and a greater urea pool size^9^.

Although the amount of hippuric acid excreted with urine did not differ between phenotypes, plasma hippuric acid concentration was greater in HMU than LMU cows. In the liver, hippuric acid is formed by the conjugation of glycine and benzoic acid, catalyzed by hippurate hydrolase^13^. We obtained no data about this enzyme from our proteome or mRNA studies and thus cannot exclude a role for hippurate hydrolase to account for the higher plasma hippuric acid concentration of HMU cows. Besides, increased plasma hippuric acid concentrations may be due to increased production of benzoic acids, which is formed during microbial fermentation of plant material containing 3-phenolypropionic acid and other lignin-forming phenolic compounds^28,29^. On the other hand, increased lignin degradation is directly related to higher urinary hippuric acid excretion^13^, which we, however, did not observe. Rather, the above mentioned worse renal performance of HMU cows seem to account for the accumulation of hippuric acid in plasma.

A further portion of urinary N excretion originates from microbial nucleic acid degradation in the intestine. After absorption, the purine derivatives xanthine and hypoxanthine are degraded by XDH in the liver but also in plasma, mucosal epithelium, and the mammary gland, resulting in the formation of uric acid^15,30^. We found no differences in urinary uric acid excretion, however, HMU cows had higher plasma uric acid concentration than LMU animals regardless of the ration. Marshall et al. (2021)^31^ also found no alterations in the urinary excretion of purine derivatives of cows with divergent MUC breeding value, however, they did not analyse purine derivatives in plasma. Interestingly, we found lower hepatic *XDH* mRNA expression, which do not correspond with higher plasma uric acid concentrations of HMU cows. One might speculate if XDH is more active in extrahepatic tissues of HMU cows, but this hypothesis requires further analyses. However, *UOX* mRNA expression was similar between groups, suggesting a comparable degradation rate of uric acid, which is also reflected by comparable plasma allantoin concentrations. Yet, HMU cows have a lower renal clearance rate for uric acid^9^, suggesting again that the higher plasma uric acid concentrations are predominantly due to worse renal performance of these animals.

In the rate-limiting step of creatine synthesis, GATM catalyzes the conversion of glycine and arginine to guanidinoacetate and ornithine^11^. The hepatic *GATM* mRNA expression tended to be higher in LMU than in HMU cows on the NP ration. Consistent with this, plasma concentrations of glycine and its precursor serine were lower in LMU than in HMU animals on the NP ration. Further downstream, guanidinoacetate is methylated by GAMT yielding creatine, which is transported via the blood to the skeletal muscle, where it is phosphorylated and stored as creatine phosphate. The *GAMT* mRNA expression also tended to be lower in HMU than LMU cows. Furthermore, plasma creatine concentrations were higher in HMU than LMU animals on LP diet. Interestingly, plasma creatine exerts a negative feedback on GATM^32^, which likely accounts for the observed lower *GATM* mRNA expression in HMU cows. Creatine and creatine phosphate are both spontaneously converted to creatinine. Creatinine is excreted with urine at a relatively constant rate, although differences between animals have been observed^15^. Müller et al. (2021)^9^ also observed higher plasma creatine concentrations, but they also reported a greater urinary creatine excretion and clearance rate in HMU cows, which we did not observe. Besides, there are indications that a folic acid deficiency leads to a reduced creatine biosynthesis and thus plasma concentration of creatine will decrease^32^. Consistent with this, LMU cows had a higher abundance of ALDH1L1, which is related to the enzyme methylene tetrahydrofolate reductase and plays a central role in the degradation of folic acid to tetrahydrofolate. Therefore, future studies should investigate folate metabolism of cows with divergent MUC.

HMU cows showed higher abundance of proteins involved in the mitochondrial oxidative stress defense system, namely, SOD1, PRDX3 and TST. The SOD1 reduces superoxide radicals to hydrogen peroxide, the latter in turn is degraded by PRDX3 to H_2_O. During this process PRDX3 is oxidized and forms a disulfide bridge at 2 cysteine residues. The oxidized PRDX3 form functions as a chaperone, but it must be reduced by thioredoxin to enable the reduction of hydrogen peroxide^33^. TST participates in the thioredoxin system by transferring sulfur to glutathione peroxidase forming glutathione persulfide (GSSH), which in turn reduces thioredoxin^34^. We found one TST spot higher and one TST spot lower abundant in HMU compared to LMU cows, thus we can only speculate which of these two spots represent the oxidized and reduced form. Nonetheless, these results suggest that HMU cows possess a higher mitochondrial oxidative stress level than LMU cows. On the other hand, higher abundances of PRDX6, which is located in the cytosol as well as peroxisomal catalase were found in LMU than HMU cows, suggesting that LMU cows have a higher oxidative stress levels in the cytosol and in peroxisomes. Why the oxidative stress defense system is activated in different cell compartments in LMU and HMU cows needs to be elucidated in further studies.

### Diet comparisons

The dietary crude protein concentration is known to affect urea metabolism and excretion^9,35,36^. Accordingly, we observed higher plasma and milk urea concentrations, a larger urea pool and greater urinary urea and N excretions on the NP than LP diet. However, the *ARG1* mRNA expression was not affected by the diet, suggesting that urea synthesis is not controlled on the transcriptional level, as mentioned earlier^37^. However, the protein abundance of ASS1, another enzyme of the urea cycle, was higher on the LP than NP diet. ASS1 catalyzes the synthesis of arginine from citrulline and aspartate, the latter providing N from endogenous sources. These results suggest that the urea produced on the LP diet contains more endogenous than ruminally-derived N.

Plasma serine and glycine concentrations increased with decreasing crude protein intake in LMU but not HMU cows, which is in line with a previous study^9^. It seems that LMU cows possess an increased serine and glycine catabolism on the LP diet, which is reflected by a greater abundance of SHMT1 and GLYAT of cows on the LP diet. Moreover, the abundance of ALDH1L1, which forms tetrahydrofolate as cofactor of SHMT1, was also higher expressed on the LP diet, underscoring increased hepatic serine catabolism during LP feeding. Furthermore, we found an ambiguous picture for the expression of ALDH6A1, with 2 spots higher and 1 spot lower abundant during LP feeding. ALDH6A1 is involved in the conversion of branched-chain amino acids, primarily isoleucine to propionyl-CoA, but despite of its strong regulation, plasma isoleucine concentrations were not affected by diet. The AA-degrading enzymes mentioned above provide various substrates entering the TCA cycle, suggesting increased activity of the TCA cycle^16^, which is supported by the higher expression of ACO2 on the LP diet. In addition, plasma methylhistidine concentrations were reported to be higher on the LP than NP diet^9^, further indicating higher endogenous protein degradation and likely amino acid supply to the liver on the LP diet.

Except for 3-hydroxyacyl-CoA dehydrogenase, the enzymes of the mitochondrial β-oxidation pathway namely ACADM, ECHS1, and ACAA2 were more abundant on the LP than NP diet. In studies with lactating mice fed different protein levels^19,38^, upregulation of ACAA2 was also observed on a diet with reduced protein content. Thus, our results suggest that cows evolve increased fatty acid degradation on the LP diet. Perhaps the lower dietary N content diminished rumen microbial growth, which resulted in an insufficient energy supply to the host. However, the higher hepatic fat oxidation on the LP diet is not reflected by increased plasma NEFA concentrations as reported earlier^9^, but accompanied by higher GPD1 protein expression, which would increase the transfer of reducing equivalents from the cytosol to mitochondria and thus facilitate mitochondrial β-oxidation. The increased plasma AST concentration mirrors the elevated mitochondrial activity on the LP diet and has been associated with increased lipolysis^39^, but was also found in cows under heat stress^40^, which is associated with decreased feed intake and thus low protein intake.

## Conclusion

The results obtained do not support our hypothesis. Although dairy cows with intrinsically high milk urea concentration have higher plasma urea, uric acid, and hippuric acid concentrations, these differences are not associated with increased activation of the hepatic urea cycle or pathways synthesizing non-urea N metabolites, at least not when evaluated by proteome and mRNA expression analyses. Instead, cows secreting high milk urea concentrations show signs of a higher mitochondrial oxidative stress level, which was unexpected. However, other factors, such as an increased ruminal NH_3_ synthesis and a lower renal clearance rate seem to account more for the differences in milk urea concentrations. In addition, plasma, milk and urinary urea concentrations increased with increasing N intake, but we did not observe differences in the mRNA expression of urea cycle enzymes, indicating that urea synthesis is not controlled on the transcriptional level. The reason for increased expression of hepatic enzymes catabolizing fatty acids during feeding a reduced N plane remains to be investigated in future studies.

## Supplementary Information

Supplementary Information.

## Data Availability

The mass spectrometry proteomics data have been deposited to the ProteomeXchange Consortium via the PRIDE^21^ partner repository with the with identifier PXD033412 (http://www.ebi.ac.uk/pride/archive/projects/PXD033412). Further data are available from the corresponding author on request.
