# Supplementary material for: Hepatic urea, creatinine and uric acid metabolism in dairy cows with divergent milk urea concentrations
Source: Sci Rep. 2022 Oct 20;12:17593. doi: 10.1038/s41598-022-22536-y (PMC9585098; doi:10.1038/s41598-022-22536-y)

Hepatic urea, creatinine and uric acid metabolism in dairy cows with divergent milk urea concentrations

Marie C. Prahl, Carolin B.M. Müller, Dirk Albrecht, Franziska Koch, Klaus Wimmers, Björn Kuhla

Supplementary Material

Supplemental Table 1: Primer sequences 5‘ – 3‘

Gene[1]	Primer sequences	Amplicon length [bp]	PCR efficiency [%]	References[2]
ARG1	F: CCATCTTTCACGCCAGCTAC	224	1.72	Takagi M., 2008
	R: TGGTTACCCTCTCGAGCAAC			
GAMT	F: ACGATACATACCCGCTGTCG	112	1.80	
	R: AGGTTACAGTAGGTGAGGACA			
GATM	F: TGCTGAAATTGAAGAAATGTGCA	127	1.83	
	R: CGCGCCATATAAACCCGTAG			
NOS2	F: ATCTGCAGACACGTGCGTTA	105	1.79	Shen et al., 2019
	R: GTTCCAGACCCGGAAGTCAT			
UOX	F: ATGGAAATCAGTCTGCCGAAC	159	1.83	
	R: TCACAGCCTTGAAGTCAGC			
XDH	F: AAGTCACGGCTCTCAGTGT	192	1.82	Bühler et al., 2016
	R: CCACAGCATCCACCATTCTT			
PPIA	F: GGATTTATGTGCCAGGGTGGTGA	120	1.81	Bonnet et al., 2013
	R: CAAGATGCCAGGACCTGTATG			
EMD	F: GCCCTCAGCTTCACTCTCAGA	100	1.88	Saremi et al., 2012
	R: GAGGCGTTCCCGATCCTT			

[1] ARG1, Arginase 1; GAMT, gauanidinoacetate N-methyltransferase; GATM, arginine:glycine aminotransferase; NOS2, nitric oxide synthase; UOX, urate oxidase; XDH, xanthine dehydrogenase; PPIA, peptidylprolyl isomerase A; EMD, emerin.

[2] Takagi M., J Anim Sci. 2008; 86(7):1526-32.; Shen et al., J Dairy Sci. 2019; 102(11):10554-10563.; Bühler et al., J Anim Physiol Anim Nutr. 2018; 102:e87–e98.; Bonnet et al., Animal (2013), 7:8, pp 1344-1353.; Saremi et al., J.Dairy Sci. 2012; 95:3131-3138.

Supplementary Table 2a. MALDI-TOF-MS based protein identification of differentially expressed spots between dairy cows with high (HMU) and low (LMU) milk urea concentration.

Spot ID	Protein Name	Accession Number	Protein MW	Peptide Count	Protein Score	Protein Score C.I. %	Total Ion Score	Total Ion Score C.I. %	Total MS Ion Cluster Area	MS Ion Cluster Area Matched %
63	Albumin OS=Bos taurus OX=9913 GN=ALB PE=1 SV=4	P02769	69248.44	16.0	238.0	100.0	146.12	100.0	254647	79.1
95	Formimidoyltetrahydrofolate cyclodeaminase OS=Bos indicus x Bos taurus OX=30522 GN=FTCD PE=3 SV=1	A0A4W2F282	59040.56	15	334.0	100.0	241.24	100.0	373664	87.2
113	ATP synthase subunit beta, mitochondrial OS=Bos taurus OX=9913 GN=ATP5F1B PE=1 SV=2	P00829	56248.57	16	418.0	100.0	297.42	100.0	662278	149.9
116	ATP synthase subunit beta, mitochondrial OS=Bos taurus OX=9913 GN=ATP5F1B PE=1 SV=2	P00829	56248.57	11	183.0	100.0	118.25	100.0	111432	45.1
125	26S proteasome regulatory subunit 6B OS=Bos taurus OX=9913 GN=PSMC4 PE=2 SV=1	Q3T030	47336.54	11	100.0	100.0	37.68	95.3	121395	20.2
129	Albumin OS=Bos taurus OX=9913 GN=ALB PE=4 SV=1	A0A3Q1LNN7	66197.13	6	163.0	100.0	129.67	100.0	96370	147.4
133	3-ketoacyl-CoA thiolase, mitochondrial OS=Bos taurus OX=9913 GN=ACAA2 PE=1 SV=1	A0A3Q1MDH5	41915.29	11.0	311.0	100.0	232.83	100.0	197351	98.4
148	Reticulocalbin 1 OS=Bos taurus OX=9913 GN=RCN1 PE=1 SV=2	G3N2L2	38796.95	11.0	170.0	100.0	90.34	100.0	104052	96.9
158	PKS_ER domain-containing protein OS=Bos indicus x Bos taurus OX=30522 PE=3 SV=1	A0A4W2DFI6	44060.93	9	206.0	100.0	151.65	100.0	97389	74.0
164	Galactokinase OS=Bos taurus OX=9913 GN=GALK1 PE=2 SV=2	A6H768	42200.56	7	196.0	100.0	137.86	100.0	26404	99.0
184	Dihydrodiol dehydrogenase 3 OS=Bos taurus OX=9913 PE=2 SV=1	P52898	36761.21	3	98.7	100.0	76.11	100.0	17981	42.6
188	Glyceraldehyde-3-phosphate dehydrogenase OS=Bos taurus OX=9913 GN=GAPDH PE=1 SV=4	P10096	35845.30	5.0	94.8	100.0	71.71	100.0	128849	36.2
191	PKS_ER domain-containing protein OS=Bos indicus x Bos taurus OX=30522 GN=CRYZ PE=3 SV=1	A0A4W2GVP7	36944.59	3	93.2	100.0	75.52	100.0	26005	63.2
203	Malate dehydrogenase, mitochondrial OS=Bos taurus OX=9913 GN=MDH2 PE=1 SV=1	Q32LG3	35645.77	8	178.0	100.0	127.29	100.0	122742	46.2
206	10-formyltetrahydrofolate dehydrogenase OS=Bos taurus OX=9913 GN=ALDH1L1 PE=2 SV=1	A7YY67	98675.61	12	90.7	100.0	41.70	98.2	181608	18.4
216	Thiosulfate sulfurtransferase OS=Bos taurus OX=9913 GN=TST PE=1 SV=3	P00586	33274.74	10	183.0	100.0	111.89	100.0	131243	32.7
225	Glycine N-acyltransferase OS=Bos taurus OX=9913 GN=GLYAT PE=1 SV=2	Q2KIR7	33884.97	8	122.0	100.0	61.93	100.0	80967	92.9
240	Endoplasmic reticulum resident protein 29 OS=Bos taurus OX=9913 GN=ERP29 PE=1 SV=2	P81623	28787.91	8.0	106.0	100.0	55.72	99.9	207328	64.6
241	Glutathione transferase OS=Bos taurus OX=9913 GN=GSTM4 PE=2 SV=1	A1A4L7	25653.02	2	139.0	100.0	118.04	100.0	7037	80.0
256	Peptide-methionine (S)-S-oxide reductase OS=Bos indicus x Bos taurus OX=30522 GN=MSRA PE=3 SV=1	A0A4W2H2Q0	21397.71	8	78.1	99.8	11.46	0.0	228805	54.7
275	ATP synthase subunit alpha, mitochondrial OS=Bos taurus OX=9913 GN=ATP5F1A PE=1 SV=1	P19483	59682.70	7	306.0	100.0	261.44	100.0	55183	135.2
295	2-oxo-4-hydroxy-4-carboxy-5-ureidoimidazoline decarboxylase OS=Bos taurus OX=9913 GN=URAD PE=2 SV=1	A5PJD0	18929.91	8	228.0	100.0	151.36	100.0	431513	128.2
328	Superoxide dismutase [Cu-Zn] OS=Bos taurus OX=9913 GN=SOD1 PE=1 SV=2	P00442	15672.82	7	169.0	100.0	101.25	100.0	273382	48.1
337	Cytochrome b5 type A OS=Bos indicus x Bos taurus OX=30522 GN=CYB5A PE=3 SV=1	A0A4W2I6Q0	11175.51	3.0	97.4	100.0	73.94	100.0	88207	9.4
354	Carrier domain-containing protein OS=Bos indicus x Bos taurus OX=30522 GN=ALDH1L1 PE=3 SV=1	A0A4W2HFQ9	87482.93	10.0	212.0	100.0	161.89	100.0	54422	43.9
377	Aldedh domain-containing protein OS=Bos indicus x Bos taurus OX=30522 GN=ALDH9A1 PE=3 SV=1	A0A4W2E109	65935.44	5	160.0	100.0	146.11	100.0	384768	135.8
401	Aldo_ket_red domain-containing protein OS=Bos indicus x Bos taurus OX=30522 PE=3 SV=1	A0A4W2E8C6	34359.23	10.0	222.0	100.0	146.91	100.0	417839	41.7
412	Uncharacterized protein OS=Bos indicus x Bos taurus OX=30522 GN=MROH2B PE=4 SV=1	A0A4W2FFC0	174551.16	22	74.4	99.5			112662	17.3
465	Phosphoenolpyruvate carboxykinase (GTP) OS=Bos taurus OX=9913 GN=PCK2 PE=1 SV=1	F1MDS3	70604.73	12	178.0	100.0	120.55	100.0	346422	61.1
507	Methylmalonate-semialdehyde dehydrogenase [acylating], mitochondrial OS=Bos taurus OX=9913 GN=ALDH6A1 PE=3 SV=1	A0A3Q1LN22	64037.30	15	237.0	100.0	135.08	100.0	533407	92.2
509	Glutamate dehydrogenase (NAD(P)(+)) OS=Bos taurus OX=9913 GN=GLUD1 PE=1 SV=1	A0A140T871	58009.68	20	234.0	100.0	89.20	100.0	605868	83.7
515	Omega-amidase NIT2 OS=Bos taurus OX=9913 GN=NIT2 PE=2 SV=1	Q2T9R6	61592.42	6	90.6	100.0	54.48	99.9	63504	94.1
550	Albumin OS=Bos taurus OX=9913 GN=ALB PE=4 SV=1	A0A3Q1LNN7	30498.56	9	91.1	100.0	57.54	100.0	104621	117.0
570	Endoplasmic reticulum chaperone BiP OS=Bos taurus OX=9913 GN=HSPA5 PE=2 SV=1	Q0VCX2	66197.13	18	339.0	100.0	229.57	100.0	187363	66.5
574	Albumin OS=Bos taurus OX=9913 GN=ALB PE=4 SV=1	A0A140T897	69278.45	17.0	328.0	100.0	224.92	100.0	477446	127.9
580	Protein disulfide-isomerase A3 OS=Bos taurus OX=9913 GN=PDIA3 PE=2 SV=1	P38657	56893.87	10.0	66.6	96.9	25.84	28.3	68482	21.9
582	Retinal dehydrogenase 1 OS=Bos taurus OX=9913 GN=ALDH1A1 PE=1 SV=3	P48644	54770.61	5	141.0	100.0	105.55	100.0	12500	88.7
601	Aldedh domain-containing protein OS=Bos indicus x Bos taurus OX=30522 GN=ALDH9A1 PE=3 SV=1	A0A4W2E109	65935.44	9.0	135.0	100.0	99.90	100.0	71240	39.2
611	Tubulin beta-4A chain OS=Bos taurus OX=9913 GN=TUBB4A PE=2 SV=1	Q3ZBU7	72355.53	8	172.0	100.0	119.69	100.0	33157	69.6
619	Alpha-1-acid glycoprotein OS=Bos taurus OX=9913 GN=agp PE=2 SV=2	Q5GN72	23143.67	6.0	188.0	100.0	142.68	100.0	265453	132.0
621	N(G),N(G)-dimethylarginine dimethylaminohydrolase 1 OS=Bos taurus OX=9913 GN=DDAH1 PE=1 SV=3	P56965	49553.90	8	96.7	100.0	45.58	99.5	397242	26.9
626	Actin gamma 1 OS=Bos indicus x Bos taurus OX=30522 GN=ACTG1 PE=3 SV=1	A0A4W2DZW4	31269.07	3	172.0	100.0	149.00	100.0	28581	111.5
644	Catalase OS=Bos taurus OX=9913 GN=CAT PE=1 SV=3	P00432	36519.00	14	199.0	100.0	108.75	100.0	380180	71.6
655	Aldedh domain-containing protein OS=Bos indicus x Bos taurus OX=30522 GN=ALDH6A1 PE=3 SV=1	A0A4W2ER80	59877.75	7	134.0	100.0	68.02	100.0	11122	106.4
665	Heat shock 27 kDa protein OS=Bos taurus OX=9913 GN=HSPB1 PE=2 SV=1	A0A1C9EIX3	59125.53	8	113.0	100.0	44.66	99.2	207515	42.9
669	Proteasome (Prosome, macropain) activator subunit 1 (PA28 alpha) OS=Bos taurus OX=9913 GN=PSME1 PE=2 SV=1	Q2KJE7	28675.06	11	180.0	100.0	110.02	100.0	95956	55.8
676	Thioredoxin-dependent peroxide reductase, mitochondrial OS=Bos taurus OX=9913 GN=PRDX3 PE=1 SV=2	P35705	28177.42	4	211.0	100.0	189.56	100.0	434588	122.1
683	Peroxiredoxin-6 OS=Bos taurus OX=9913 GN=PRDX6 PE=1 SV=3	O77834	25051.14	4	158.0	100.0	119.17	100.0	20909	112.5
712	Glycine N-acyltransferase OS=Bos taurus OX=9913 GN=GLYAT PE=1 SV=2	Q2KIR7	33884.97	7.0	83.5	99.9	39.26	96.4	124534	63.9
713	Thiosulfate sulfurtransferase OS=Bos taurus OX=9913 GN=TST PE=1 SV=3	P00586	33274.74	5	78.9	99.8	54.29	99.9	85197	53.8
714	Thiosulfate sulfurtransferase OS=Bos taurus OX=9913 GN=TST PE=1 SV=3	P00586	33274.74	7.0	93.1	100.0	51.47	99.8	80335	23.8
718	Globin A1 OS=Bos taurus OX=9913 GN=HBB PE=3 SV=1	D4QBB4	15944.31	7.0	89.1	100.0	30.49	73.4	125140	34.9
734	HIT domain-containing protein OS=Bos indicus x Bos taurus OX=30522 PE=4 SV=1	A0A4W2GQ06	13828.07	2	64.1	94.6	49.54	99.7	115413	65.5

Supplementary Table 2b. MALDI-TOF-MS based protein identification of differentially expressed spots between dairy cows fed a normal (NP) or low dietary crude protein level (LP).

Spot ID	Protein Name	Accession Number	Protein MW	Peptide Count	Protein Score	Protein Score C.I. %	Total Ion Score	Total Ion Score C.I. %	Total MS Ion Cluster Area	MS Ion Cluster Area Matched %
78	60 kDa chaperonin (Fragment) OS=Bos taurus OX=9913 GN=HSPD1 PE=2 SV=1	Q3T179	42137.67	12	233.0	100.0	151.02	100.0	355473	60.8
92	60 kDa chaperonin (Fragment) OS=Bos taurus OX=9913 GN=HSPD1 PE=2 SV=1	Q3T179	42137.67	6	123.0	100.0	95.27	100.0	170582	43.8
112	Serine hydroxymethyltransferase OS=Bos taurus OX=9913 GN=SHMT1 PE=3 SV=1	A0A140T896	52934.96	12	185.0	100.0	116.91	100.0	107792	52.9
115	ATP synthase subunit beta, mitochondrial OS=Bos taurus OX=9913 GN=ATP5F1B PE=1 SV=2	P00829	56248.57	17	462.0	100.0	330.11	100.0	321772	137.6
127	Protein disulfide-isomerase OS=Bos taurus OX=9913 GN=P4HB PE=1 SV=1	A0A3Q1LPQ0	54365.41	8.0	117.0	100.0	80.56	100.0	57708	17.9
131	Albumin OS=Bos taurus OX=9913 GN=ALB PE=4 SV=1	A0A3Q1LNN7	66197.13	9.0	228.0	100.0	189.28	100.0	907380	105.4
137	Argininosuccinate synthase OS=Bos indicus x Bos taurus OX=30522 GN=ASS1 PE=3 SV=1	A0A4W2HQA2	44636.98	7.0	68.5	98.0	34.29	89.8	405802	40.9
144	3-ketoacyl-CoA thiolase, mitochondrial OS=Bos taurus OX=9913 GN=ACAA2 PE=2 SV=1	Q3T0R7	42104.50	9	140.0	100.0	53.24	99.7	22316	55.1
183	Glyceraldehyde-3-phosphate dehydrogenase OS=Bos taurus OX=9913 GN=GAPDH PE=1 SV=4	P10096	35845.30	4	129.0	100.0	106.45	100.0	57516	84.0
184	Retinal dehydrogenase 1 OS=Bos taurus OX=9913 GN=ALDH1A1 PE=1 SV=3	P48644	54770.61	9.0	129.0	100.0	89.38	100.0	171238	16.4
195	Aldo_ket_red domain-containing protein OS=Bos indicus x Bos taurus OX=30522 GN=LOC113903410 PE=3 SV=1	A0A4W2HP82	34331.13	2	123.0	100.0	106.51	100.0	6731	67.5
200	Glycerol-3-phosphate dehydrogenase [NAD(+)], cytoplasmic OS=Bos taurus OX=9913 GN=GPD1 PE=2 SV=3	Q5EA88	37623.42	9.0	161.0	100.0	102.27	100.0	140165	58.5
206	10-formyltetrahydrofolate dehydrogenase OS=Bos taurus OX=9913 GN=ALDH1L1 PE=2 SV=1	A7YY67	98675.61	12	90.7	100.0	41.70	98.2	181608	18.4
228	Enoyl-CoA hydratase 1 OS=Bos taurus OX=9913 GN=ECH1 PE=1 SV=2	F6PRB5	35240.02	11.0	437.0	100.0	352.55	100.0	161452	117.5
232	10-formyltetrahydrofolate dehydrogenase OS=Bos indicus x Bos taurus OX=30522 GN=ALDH1L1 PE=3 SV=1	A0A4W2FME8	98718.62	3	82.5	99.9	68.83	100.0	7999	53.1
234	Enoyl-CoA hydratase, short chain 1 OS=Bos indicus x Bos taurus OX=30522 GN=ECHS1 PE=3 SV=1	A0A4W2CIV9	38303.60	7.0	134.0	100.0	94.06	100.0	214887	22.2
238	Uncharacterized protein OS=Bos indicus x Bos taurus OX=30522 GN=ACSM1 PE=3 SV=1	A0A4W2G491	64850.80	3	235.0	100.0	216.91	100.0	15882	82.8
239	3-hydroxyacyl-CoA dehydrogenase type-2 OS=Bos taurus OX=9913 GN=HSD17B10 PE=1 SV=3	O02691	27123.28	8.0	272.0	100.0	211.26	100.0	289534	98.3
257	Es1 protein OS=Bos taurus OX=9913 GN=ES1 PE=1 SV=1	Q3T0U3	28680.95	2.0	68.2	97.9	59.66	100.0	92427	20.7
266	NAD(P)-bd_dom domain-containing protein OS=Bos indicus x Bos taurus OX=30522 GN=BLVRB PE=4 SV=1	A0A4W2EJX3	20837.80	6	157.0	100.0	96.81	100.0	53878	85.3
270	4-hydroxy-2-oxoglutarate aldolase, mitochondrial OS=Bos taurus OX=9913 GN=HOGA1 PE=3 SV=1	A0A3Q1LUQ5	45658.55	2	113.0	100.0	93.84	100.0	2651	119.6
310	Calmodulin OS=Bos indicus x Bos taurus OX=30522 PE=3 SV=1	A0A4W2D3C9	18480.48	2	161.0	100.0	137.19	100.0	5590	94.9
311	Methanethiol oxidase OS=Bos taurus OX=9913 GN=SELENBP1 PE=1 SV=1	Q2KJ32	52521.63	11.0	95.1	100.0	30.17	76.2	181503	38.6
321	Nucleoside diphosphate kinase B OS=Bos taurus OX=9913 GN=NME2 PE=1 SV=1	Q3T0Q4	17304.98	6	131.0	100.0	89.98	100.0	179894	120.0
323	Peptidyl-prolyl cis-trans isomerase OS=Bos indicus x Bos taurus OX=30522 GN=PPIA PE=3 SV=1	A0A4W2EHX4	17857.83	7	189.0	100.0	132.57	100.0	236005	94.6
337	Cytochrome b5 type A OS=Bos indicus x Bos taurus OX=30522 GN=CYB5A PE=3 SV=1	A0A4W2I6Q0	11175.51	3.0	97.4	100.0	73.94	100.0	88207	9.4
377	Aldedh domain-containing protein OS=Bos indicus x Bos taurus OX=30522 GN=ALDH9A1 PE=3 SV=1	A0A4W2E109	65935.44	5	160.0	100.0	146.11	100.0	384768	135.8
379	D-dopachrome tautomerase OS=Bos taurus OX=9913 GN=DDT PE=3 SV=1	A0A0F7RQ40	12868.77	2	89.8	100.0	70.14	100.0	17554	33.0
387	Fatty acid-binding protein, liver OS=Bos taurus OX=9913 GN=FABP1 PE=1 SV=1	P80425	14218.22	1	105.0	100.0	93.00	100.0	7679	22.4
442	Glycine N-acyltransferase OS=Bos taurus OX=9913 GN=GLYAT PE=4 SV=1	A0A3Q1MPE2	42739.48	11.0	131.0	100.0	60.63	100.0	652310	87.1
448	PKS_ER domain-containing protein OS=Bos indicus x Bos taurus OX=30522 GN=SORD PE=3 SV=1	A0A4W2CN81	38074.69	9	90.3	100.0	31.09	78.0	379869	12.9
451	Dihydrodiol dehydrogenase 3 OS=Bos taurus OX=9913 PE=2 SV=1	P52898	36761.21	7	226.0	100.0	172.26	100.0	69358	146.2
474	Beta-1 metal-binding globulin OS=Bos indicus x Bos taurus OX=30522 GN=TF PE=3 SV=1	A0A4W2F0Y8	77809.88	11.0	82.4	99.9	40.32	98.2	63079	31.5
476	Transferrin OS=Bos indicus x Bos taurus OX=30522 GN=TF PE=3 SV=1	A0A4W2DKE0	70709.71	11.0	70.9	98.9	25.15	21.3	143213	15.8
481	3-hydroxy-3-methylglutaryl coenzyme A synthase OS=Bos indicus x Bos taurus OX=30522 GN=HMGCS2 PE=3 SV=1	A0A4W2GLH2	52710.48	11	114.0	100.0	51.29	99.8	196564	85.6
499	Medium-chain specific acyl-CoA dehydrogenase, mitochondrial OS=Bos taurus OX=9913 GN=ACADM PE=2 SV=1	Q3SZB4	46543.72	17	435.0	100.0	302.98	100.0	596163	86.3
536	Nucleoside-diphosphate kinase OS=Bos indicus x Bos taurus OX=30522 GN=AK2 PE=3 SV=1	A0A4W2EIQ0	21261.08	5.0	92.4	100.0	62.77	100.0	72436	11.0
538	Enoyl-CoA hydratase, short chain 1 OS=Bos indicus x Bos taurus OX=30522 GN=ECHS1 PE=3 SV=1	A0A4W2CIV9	38303.60	9	356.0	100.0	297.37	100.0	662795	177.2
540	Peroxiredoxin-6 OS=Bos taurus OX=9913 GN=PRDX6 PE=1 SV=3	O77834	25051.14	14.0	300.0	100.0	174.37	100.0	390012	75.6
549	Heme-binding protein 1 OS=Bos taurus OX=9913 GN=HEBP1 PE=2 SV=1	Q148C9	21216.44	10	240.0	100.0	156.19	100.0	383716	90.8
550	Uncharacterized protein OS=Bos indicus x Bos taurus OX=30522 GN=ALB PE=4 SV=1	A0A4W2EVR9	69457.52	11	104.0	100.0	57.54	100.0	104621	117.5
555	Heat shock 27 kDa protein OS=Bos taurus OX=9913 GN=HSPB1 PE=2 SV=1	A0A1C9EIX3	22351.30	9.0	233.0	100.0	156.92	100.0	223994	114.2
568	Endoplasmic reticulum chaperone BiP OS=Bos taurus OX=9913 GN=HSPA5 PE=2 SV=1	Q0VCX2	72355.53	4	80.7	99.9	66.92	100.0	15400	19.6
578	Thioredoxin domain-containing protein OS=Bos indicus x Bos taurus OX=30522 GN=PDIA3 PE=3 SV=1	A0A4W2CAQ4	51889.46	5	120.0	100.0	40.07	79.4	3795	180.0
580	Protein disulfide-isomerase A3 OS=Bos taurus OX=9913 GN=PDIA3 PE=2 SV=1	P38657	56893.87	10.0	66.6	96.9	25.84	28.3	68482	21.9
582	Aldedh domain-containing protein OS=Bos indicus x Bos taurus OX=30522 GN=ALDH1A1 PE=3 SV=1	A0A4W2FVQ1	49314.00	5	143.0	100.0	105.55	100.0	12500	88.7
584	Aldedh domain-containing protein OS=Bos indicus x Bos taurus OX=30522 GN=ALDH9A1 PE=4 SV=1	A0A4W2DGD9	51676.03	7	170.0	100.0	108.02	100.0	12788	56.7
585	Aldedh domain-containing protein OS=Bos indicus x Bos taurus OX=30522 GN=ALDH2 PE=3 SV=1	A0A4W2H287	53564.31	12	228.0	100.0	152.16	100.0	249417	88.0
601	4-trimethylaminobutyraldehyde dehydrogenase OS=Bos taurus OX=9913 GN=ALDH9A1 PE=2 SV=1	Q2KJH9	53942.07	8.0	136.0	100.0	99.90	100.0	71240	39.0
611	Gluconolactonase OS=Bos indicus x Bos taurus OX=30522 GN=RGN PE=3 SV=1	A0A4W2CH43	32876.27	12	91.1	100.0			569524	25.6
617	Cathepsin Z OS=Bos taurus OX=9913 GN=CTSZ PE=2 SV=2	P05689	33864.20	3.0	87.3	100.0	75.03	100.0	65078	47.3
627	Protein disulfide-isomerase OS=Bos taurus OX=9913 GN=P4HB PE=1 SV=1	P05307	57229.79	4	154.0	100.0	125.20	100.0	10234	93.9
635	Nicotinamide N-methyltransferase-like OS=Bos taurus OX=9913 GN=LOC511161 PE=3 SV=1	V6F9B5	30017.29	3	105.0	100.0	83.70	100.0	10285	23.2
639	Aconitate hydratase, mitochondrial OS=Bos indicus x Bos taurus OX=30522 GN=ACO2 PE=3 SV=1	A0A4W2CI16	77116.51	11.0	168.0	100.0	118.32	100.0	107852	41.4
644	Catalase OS=Bos taurus OX=9913 GN=CAT PE=1 SV=3	P00432	59877.75	14	199.0	100.0	108.75	100.0	380180	71.6
655	Methylmalonate-semialdehyde dehydrogenase [acylating], mitochondrial OS=Bos taurus OX=9913 GN=ALDH6A1 PE=3 SV=1	A0A3Q1LN22	58009.68	7	133.0	100.0	68.02	100.0	11122	106.4
662	Heat shock 27 kDa protein OS=Bos taurus OX=9913 GN=HSPB1 PE=2 SV=1	A0A1C9EIX3	22351.30	9	268.0	100.0	181.34	100.0	240491	150.8
663	Heat shock 27 kDa protein OS=Bos taurus OX=9913 GN=HSPB1 PE=2 SV=1	A0A1C9EIX3	22351.30	11	265.0	100.0	163.91	100.0	608507	125.3
679	Isoform Soluble of Catechol O-methyltransferase OS=Bos taurus OX=9913 GN=COMT	A7MBI7-2	24775.78	5	303.0	100.0	271.82	100.0	400036	69.6
692	Enoyl-CoA hydratase, short chain 1 OS=Bos indicus x Bos taurus OX=30522 GN=ECHS1 PE=3 SV=1	A0A4W2CIV4	46628.28	6	93.0	100.0	70.06	100.0	57187	44.1
697	Aldedh domain-containing protein OS=Bos indicus x Bos taurus OX=30522 GN=ALDH6A1 PE=3 SV=1	A0A4W2IF52	54442.87	5.0	91.9	100.0	74.49	100.0	65921	12.4
717	Globin A1 OS=Bos taurus OX=9913 GN=HBB PE=3 SV=1	D4QBB4	15944.31	5	82.8	99.9	30.50	15.8	22120	38.8
719	Methylmalonate-semialdehyde dehydrogenase [acylating], mitochondrial OS=Bos taurus OX=9913 GN=ALDH6A1 PE=1 SV=1	Q07536	58025.66	7	81.5	99.9	52.32	99.8	99378	19.3
723	GTP cyclohydrolase 1 feedback regulatory protein OS=Bos taurus OX=9913 GN=GCHFR PE=3 SV=1	Q32L41	9550.87	4	223.0	100.0	166.93	100.0	32835	156.7
742	Globin A1 OS=Bos taurus OX=9913 GN=HBB PE=3 SV=1	D4QBB4	15944.31	8.0	120.0	100.0	48.11	99.6	338846	70.3
763	Uncharacterized protein OS=Bos indicus x Bos taurus OX=30522 GN=CBR3 PE=3 SV=1	A0A4W2G7Z6	39236.16	5	81.9	99.9	57.49	100.0	63202	16.6
768	PKS_ER domain-containing protein OS=Bos indicus x Bos taurus OX=30522 GN=CRYZ PE=3 SV=1	A0A4W2GVP7	36944.59	6	114.0	100.0	81.44	100.0	100593	85.3
774	GLOBIN domain-containing protein OS=Bos taurus OX=9913 GN=HBA1 PE=3 SV=1	A0A452DIQ5	14153.23	6.0	317.0	100.0	260.78	100.0	118726	126.5
777	GLOBIN domain-containing protein OS=Bos taurus OX=9913 GN=HBA1 PE=3 SV=1	A0A452DIQ5	14153.23	6	147.0	100.0	90.12	100.0	43317	48.5
780	Beta-2-microglobulin (Fragment) OS=Bos taurus OX=9913 PE=2 SV=1	Q862Q3	11036.53	2	70.1	98.6	56.46	99.9	70257	33.9
786	GTP cyclohydrolase 1 feedback regulatory protein OS=Bos taurus OX=9913 GN=GCHFR PE=3 SV=1	Q32L41	9550.87	4.0	208.0	100.0	174.22	100.0	264755	150.6
257a	Aldo_ket_red domain-containing protein OS=Bos indicus x Bos taurus OX=30522 GN=LOC113903410 PE=3 SV=1	A0A4W2HP82	34331.13	1	116.0	100.0	105.08	100.0	3098	127.6
528b	Quinone oxidoreductase-like protein 2 OS=Bos taurus OX=9913 PE=2 SV=2	A6QQF5	37644.31	4	86.4	100.0	57.64	100.0	7691	15.1

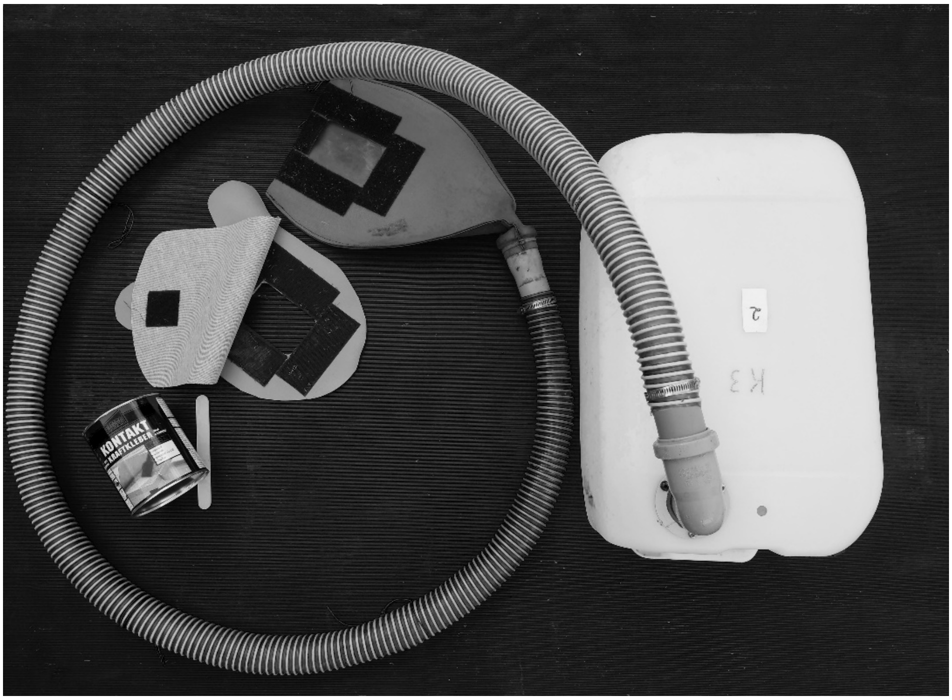

Figure 1a. Different components for urine collection: Synthetic leather urinal, sewn-on piece of synthetic leather, glue, flexible tube, and urine collection container.

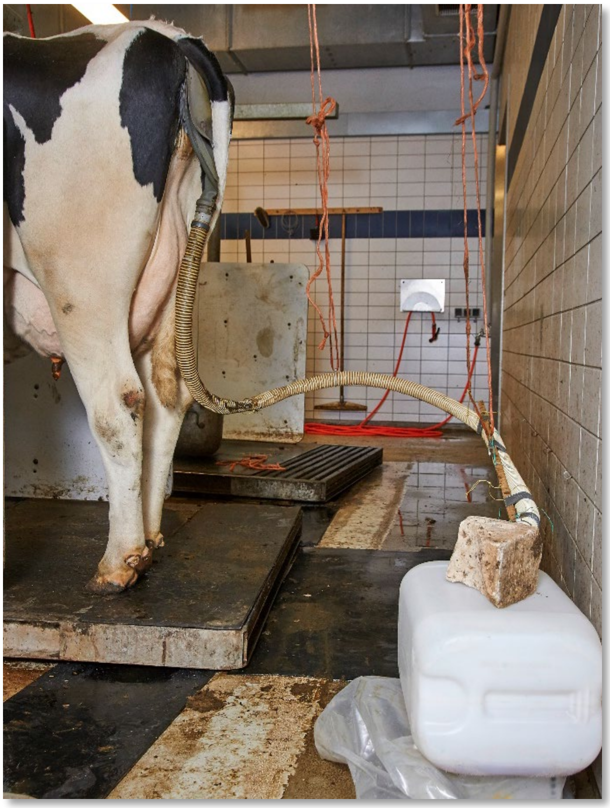

Figure 1b. Urinal attached to a dairy cow.

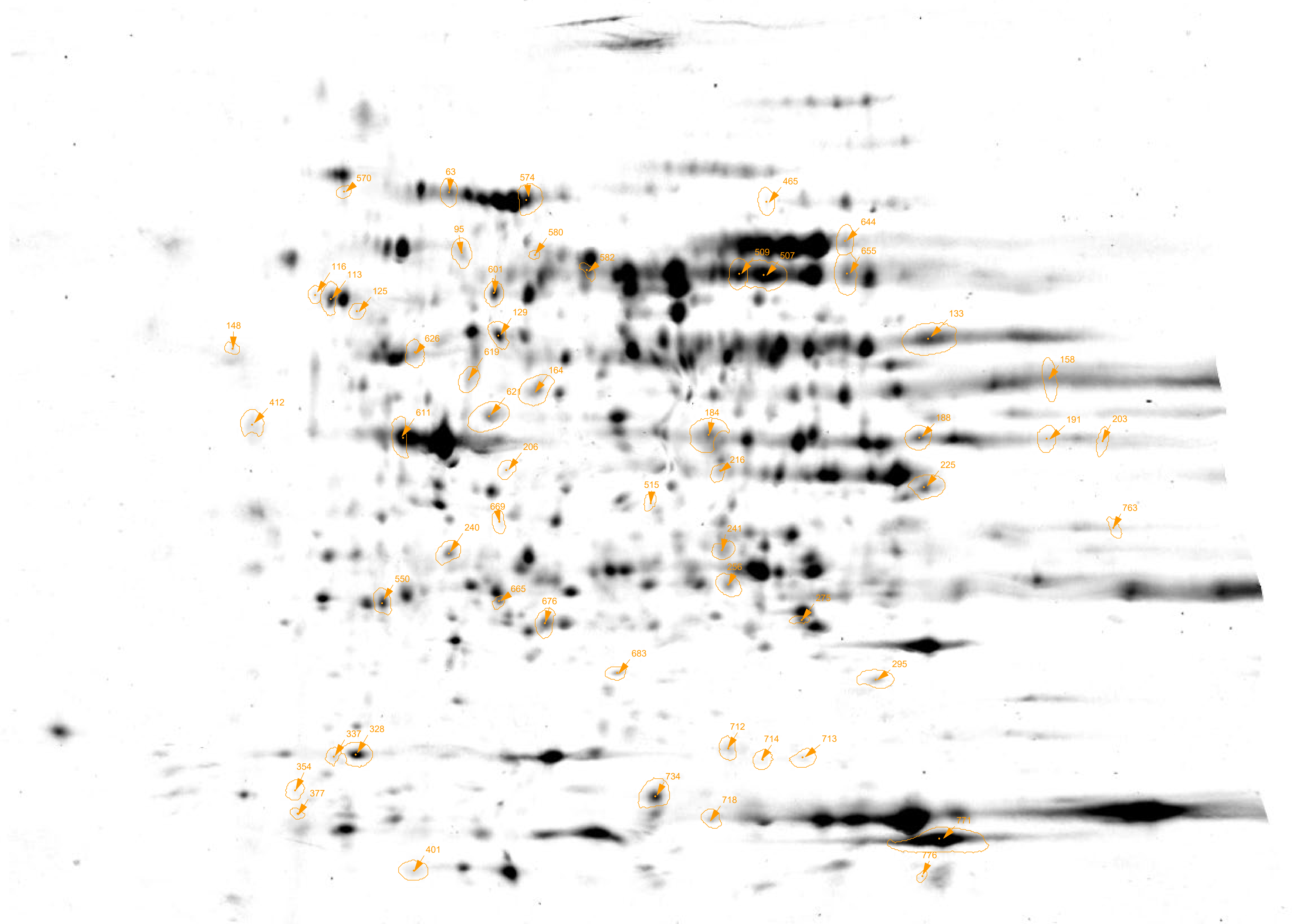

Supplementary Figure 2a. Master gel image after warping individual images in DELTA2D (version 4.6; DECODON, Greifswald, Germany; http://www.decodon.com). Spots whose abundances differ between HMU and LMU cows by *P* < 0.1 are highlighted. For their identity, see Supplementary Table 2a.

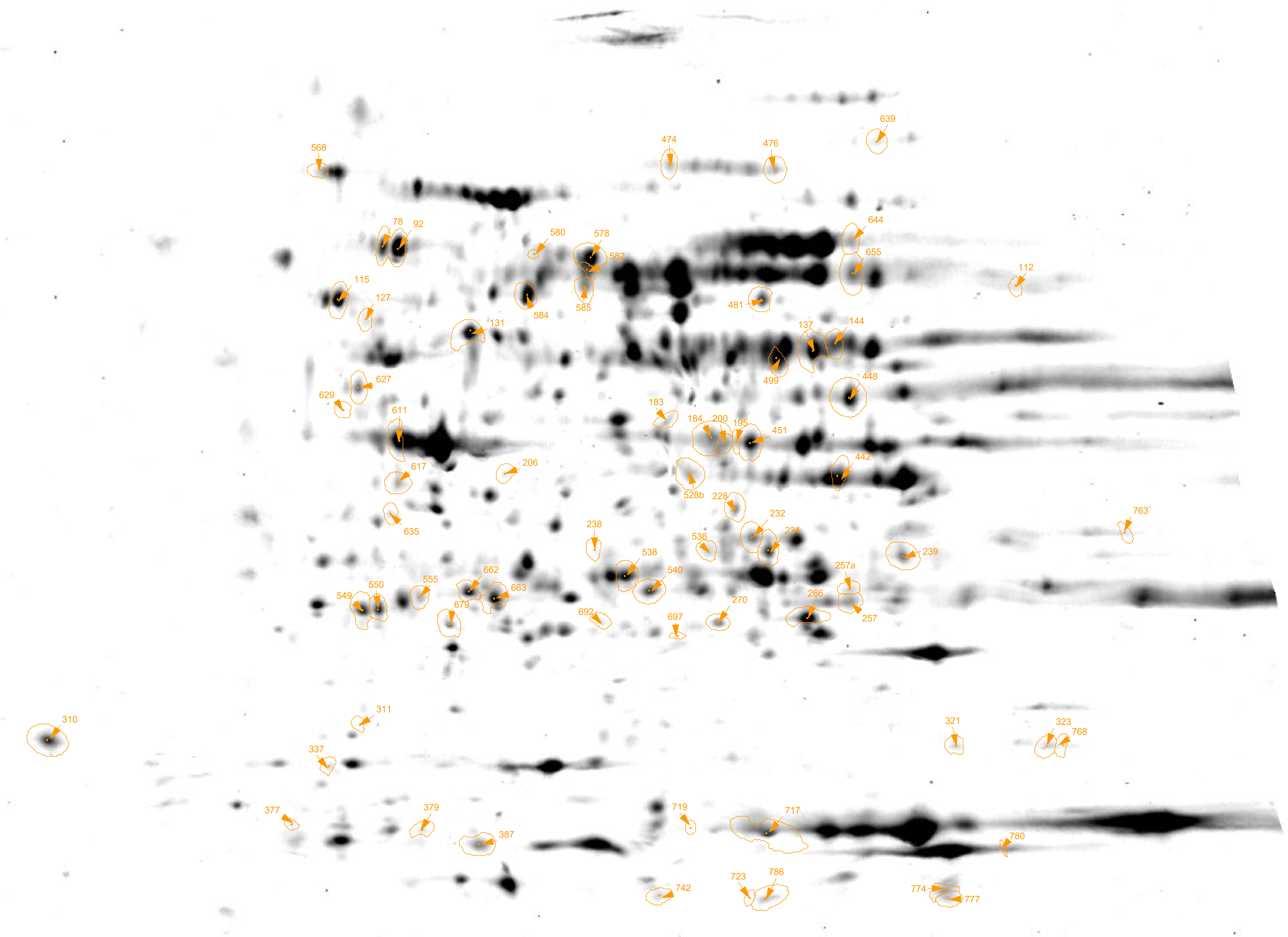

Supplementary Figure 2b. Master gel image after warping individual images in DELTA2D (version 4.6; DECODON, Greifswald, Germany; http://www.decodon.com). Spots whose abundances differ between LP and NP by $P < 0.1$ are highlighted. For their identity, see Supplementary Table 2b.

Overview about gel images on pages 9 to 80 of this file

**Group**	**Cow number**	**Technical repetition**	**Page in this file**	**Group**	**Cow number**	**Technical repetition**	**Page in this file**
HMU-LP	1	1	9	HMU-NP	1	1	27
	1	2	10		1	2	28
	2	1	11		2	1	29
	2	2	12		2	2	30
	3	1	13		3	1	31
	3	2	14		3	2	32
	4	1	15		4	1	33
	4	2	16		4	2	34
	5	1	17		5	1	35
	5	2	18		5	2	36
	6	1	19		6	1	37
	6	2	20		6	2	38
	7	1	21		7	1	39
	7	2	22		7	2	40
	8	1	23		8	1	41
	8	2	24		8	2	42
	9	1	25		9	1	43
	9	2	26		9	2	44
LMU-LP	1	1	45	LMU-NP	1	1	63
	1	2	46		1	2	64
	2	1	47		2	1	65
	2	2	48		2	2	66
	3	1	49		3	1	67
	3	2	50		3	2	68
	4	1	51		4	1	69
	4	2	52		4	2	70
	5	1	53		5	1	71
	5	2	54		5	2	72
	6	1	55		6	1	73
	6	2	56		6	2	74
	7	1	57		7	1	75
	7	2	58		7	2	76
	8	1	59		8	1	77
	8	2	60		8	2	78
	9	1	61		9	1	79
	9	2	62		9	2	80

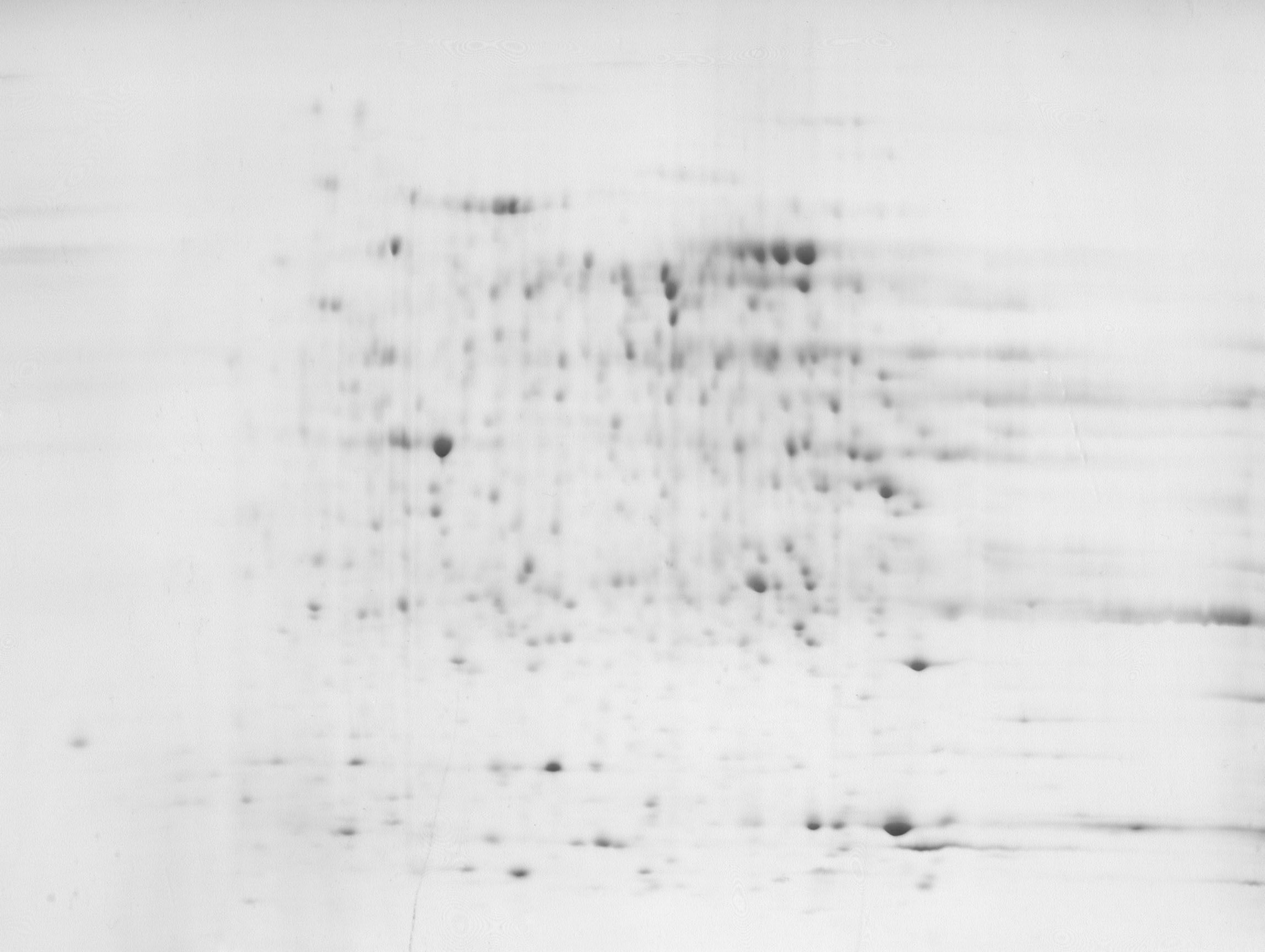

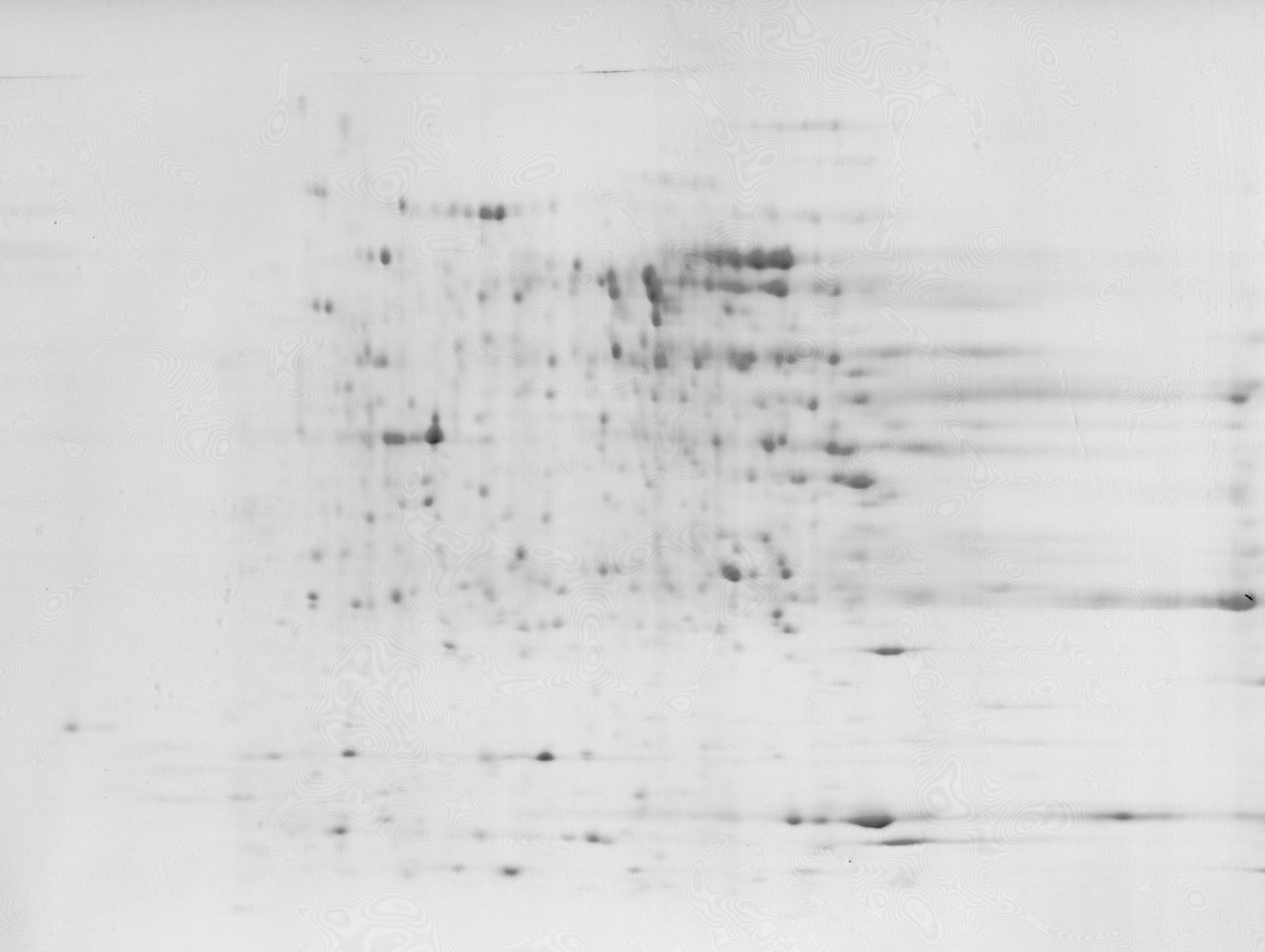

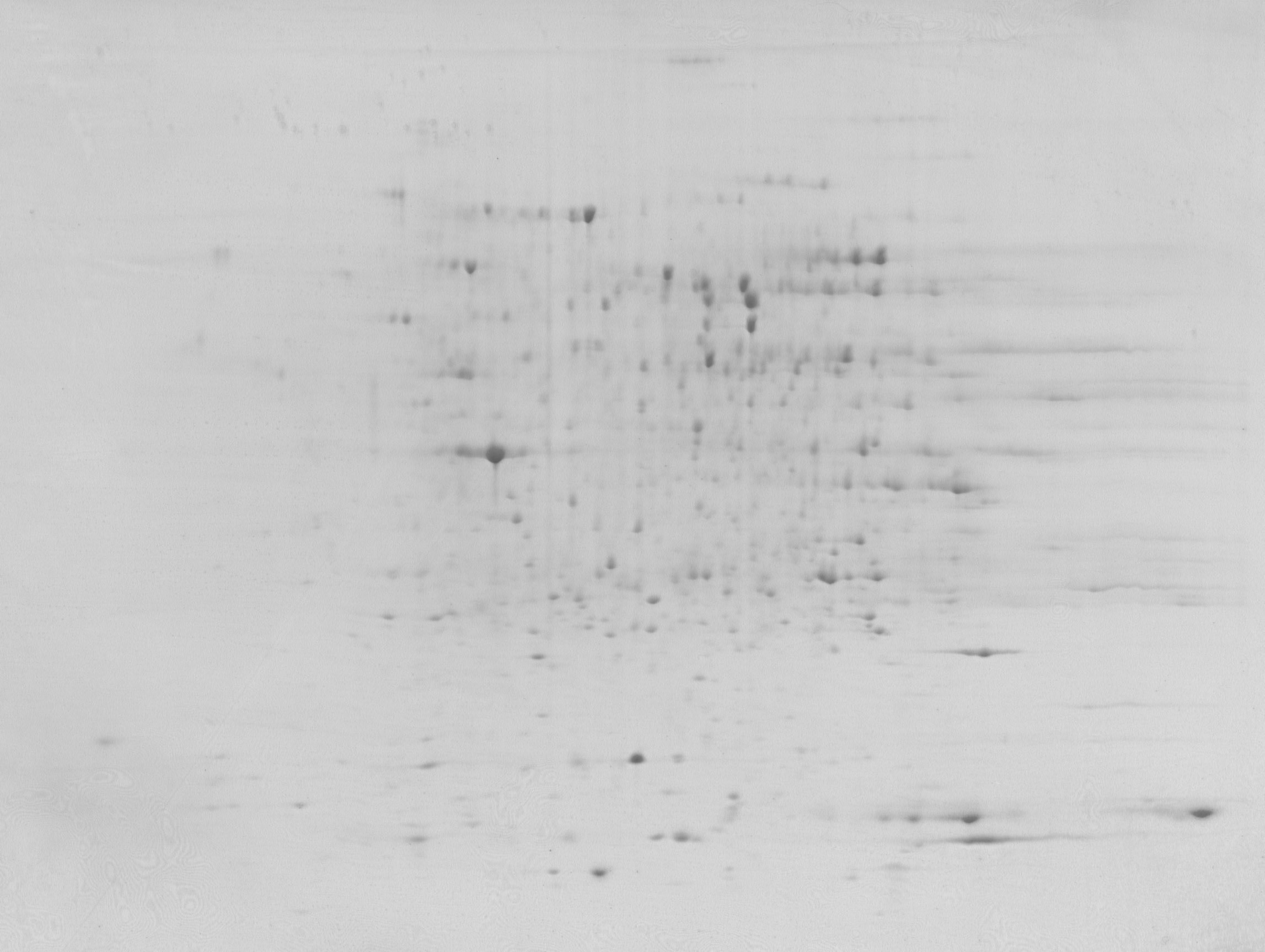

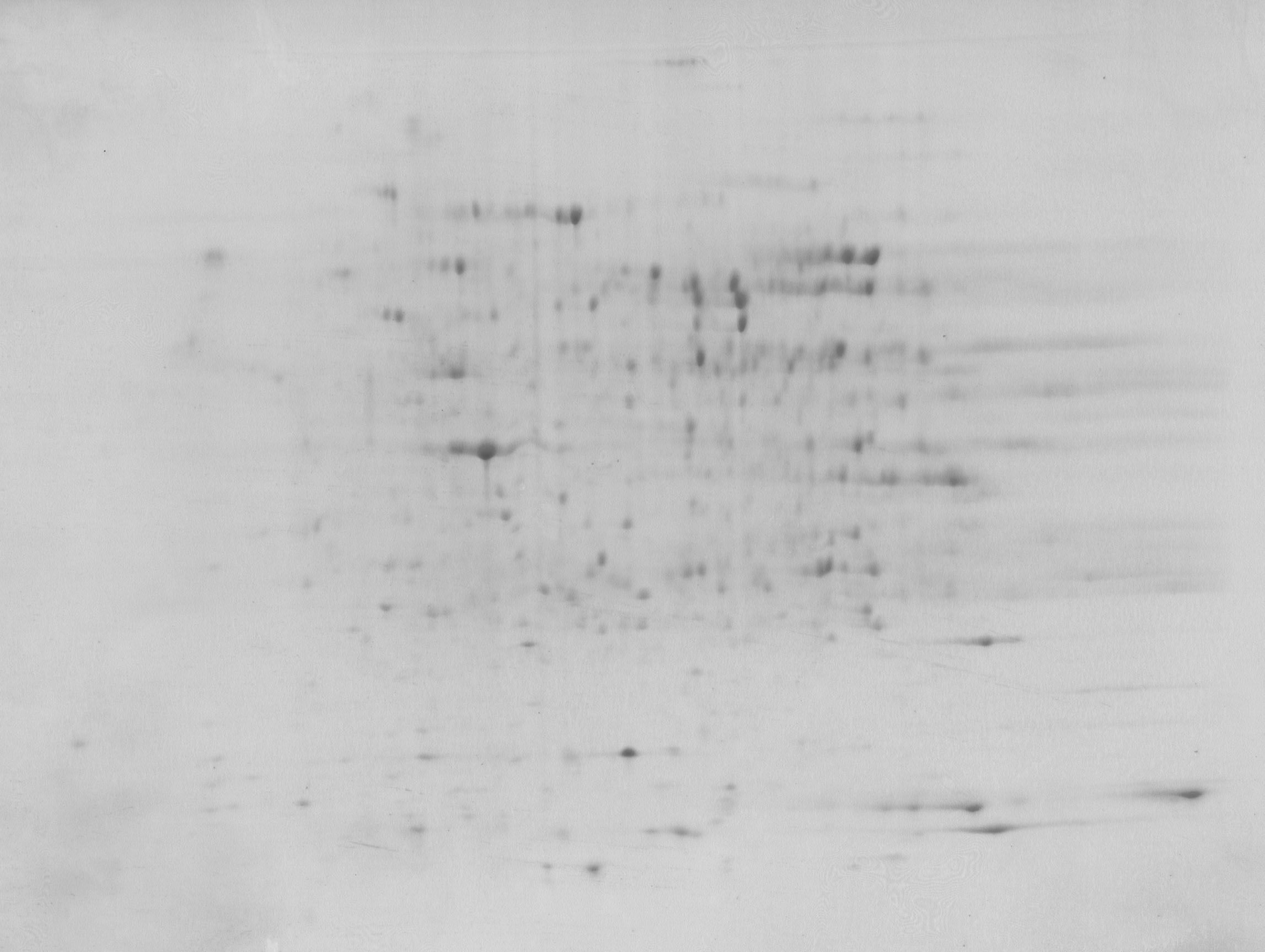

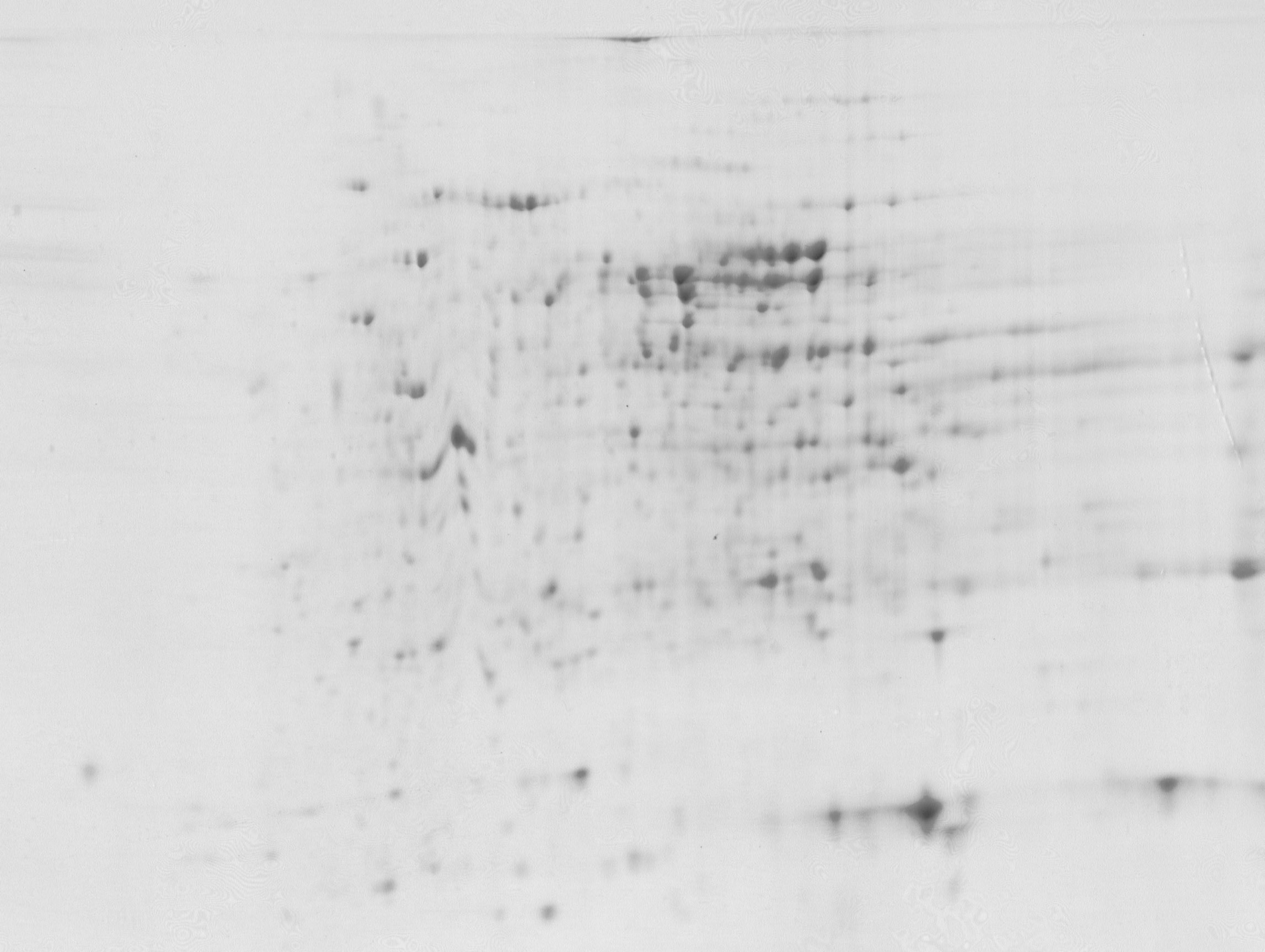

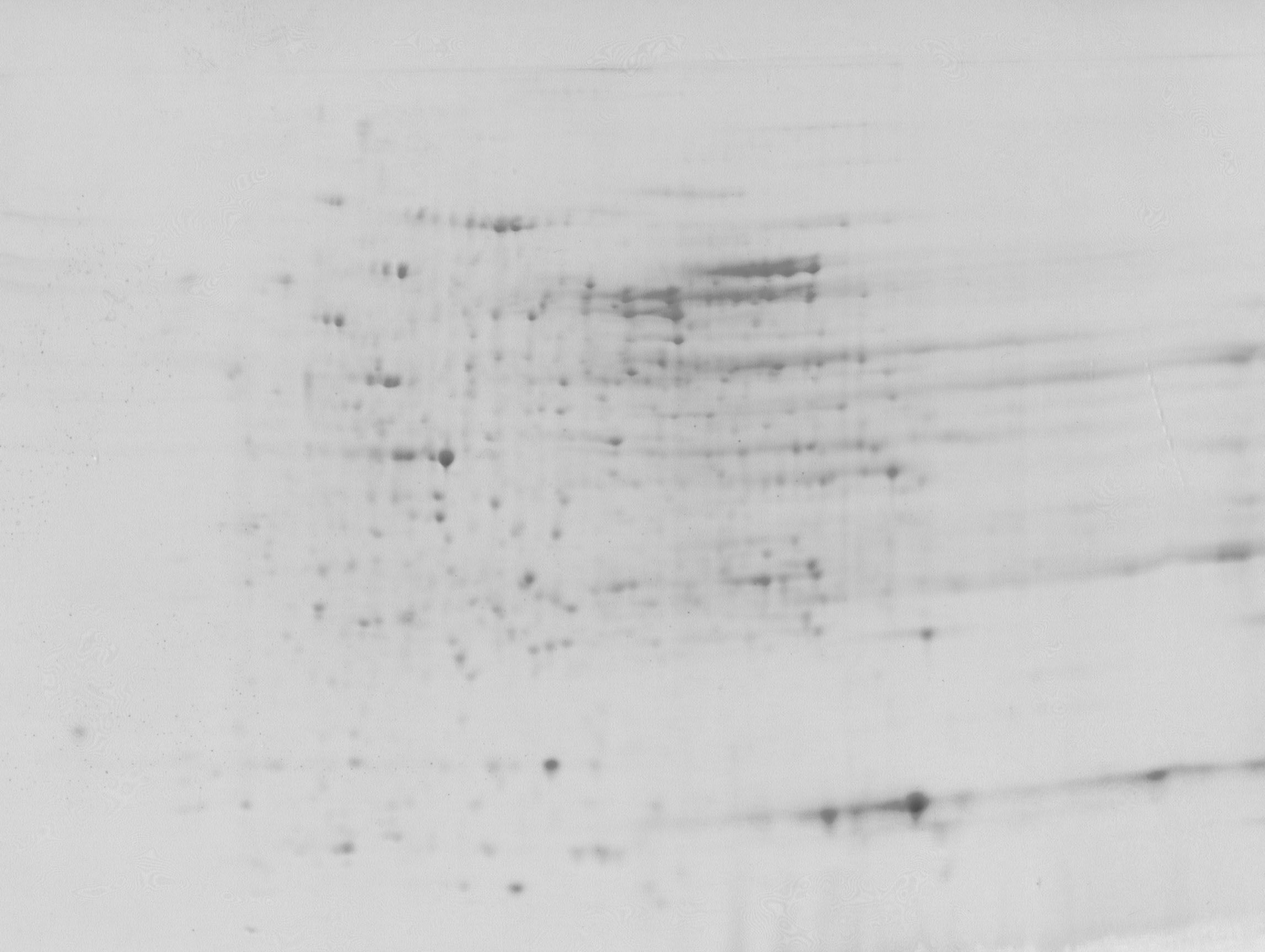

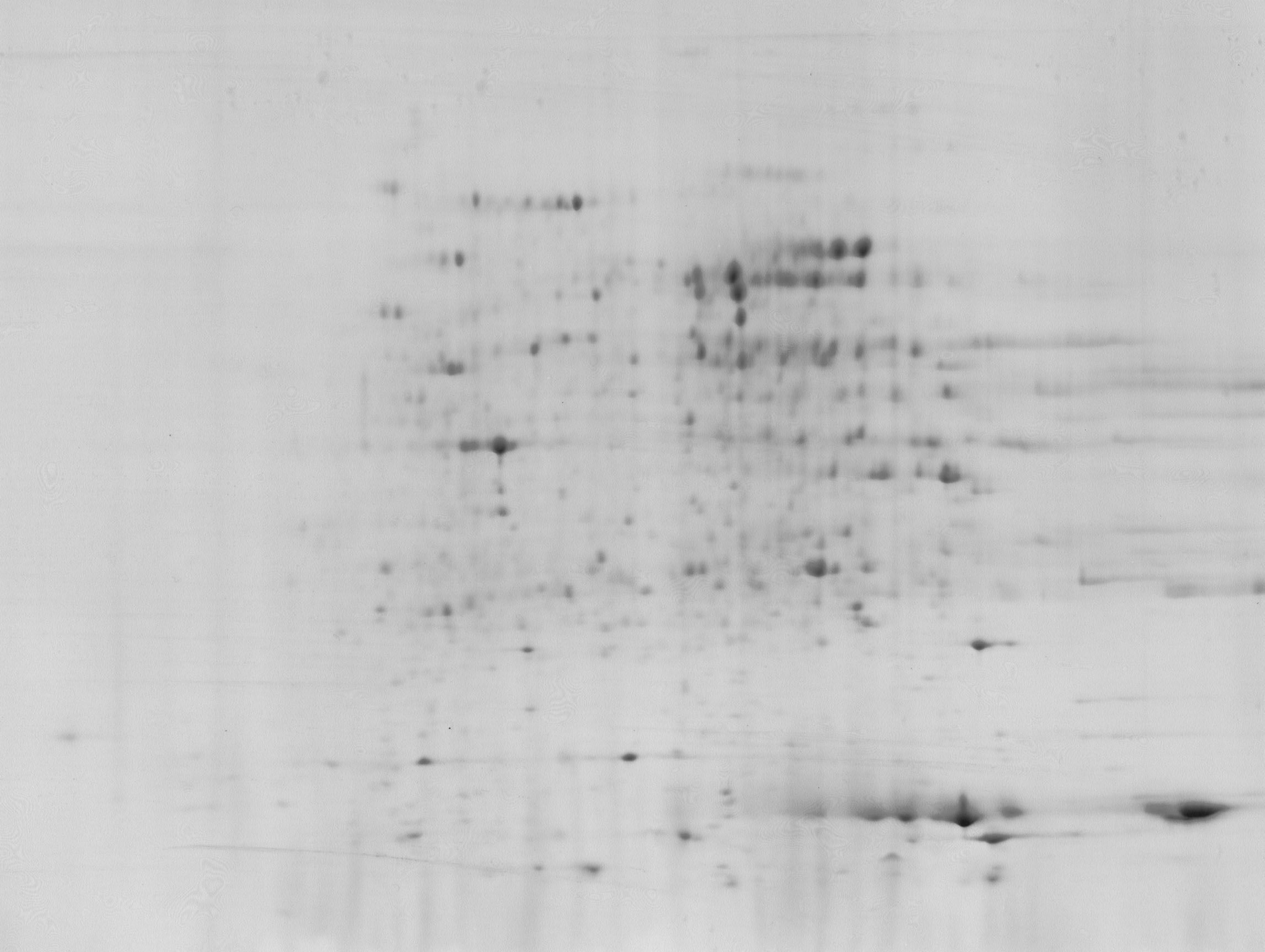

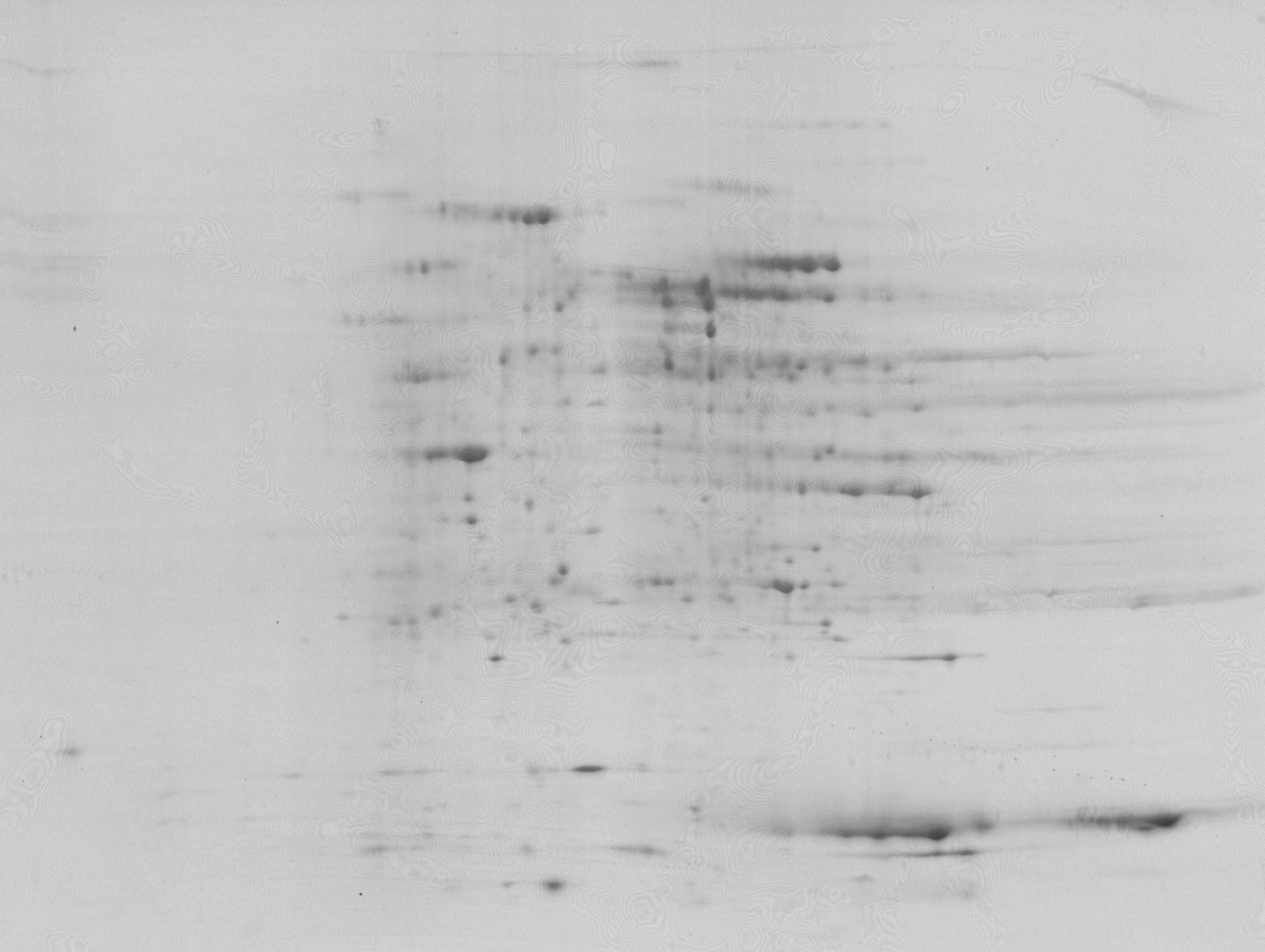

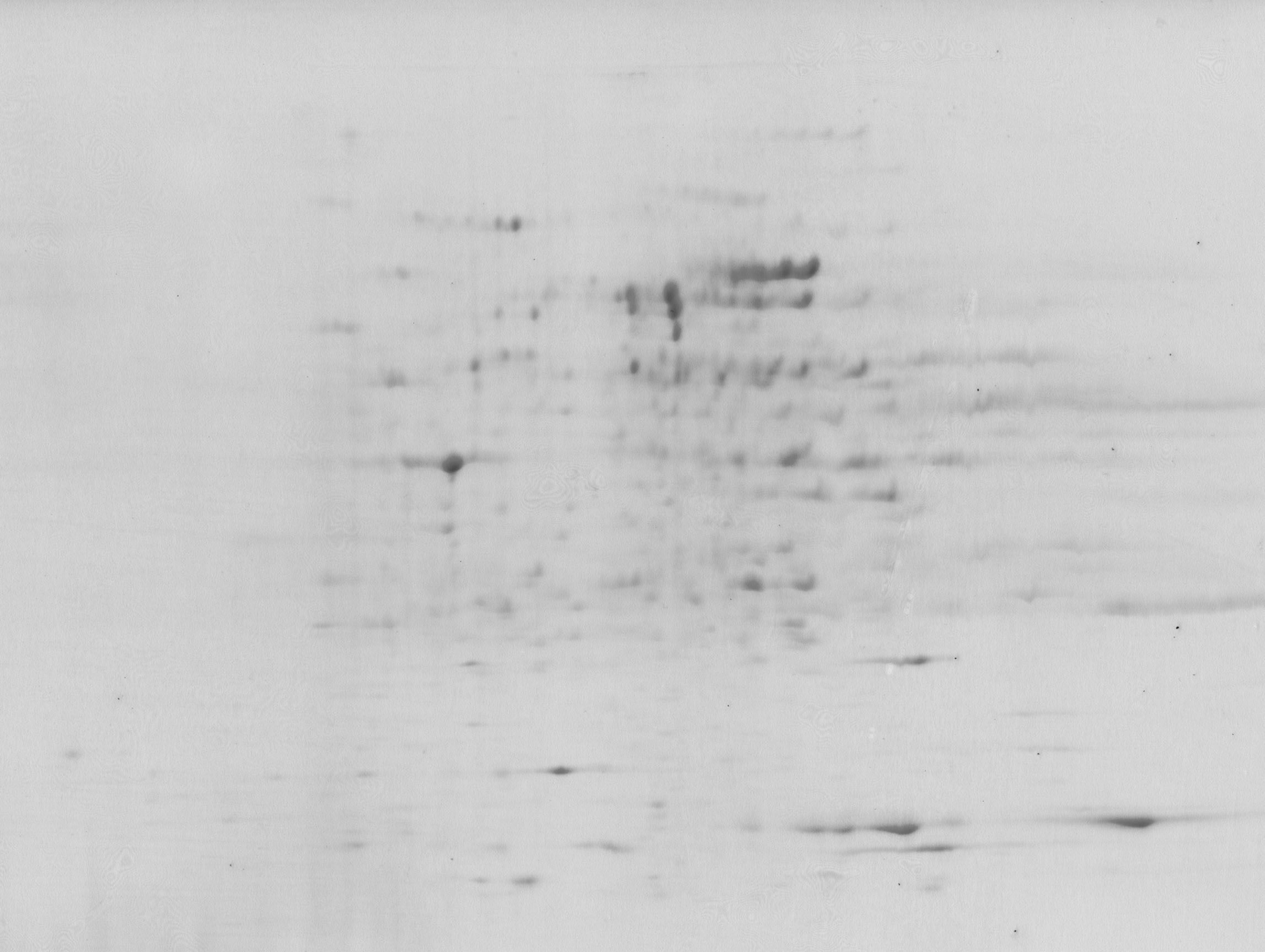

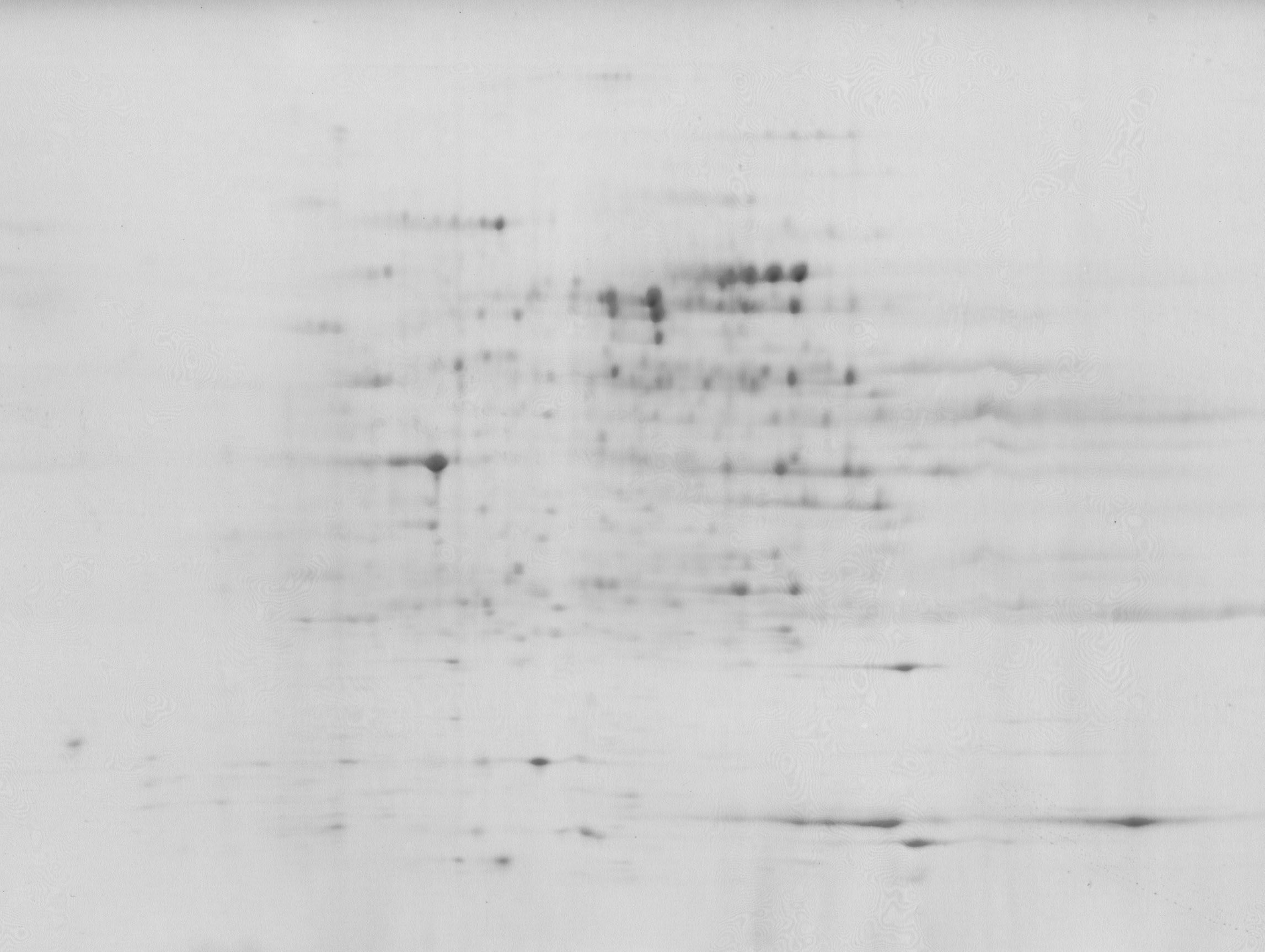

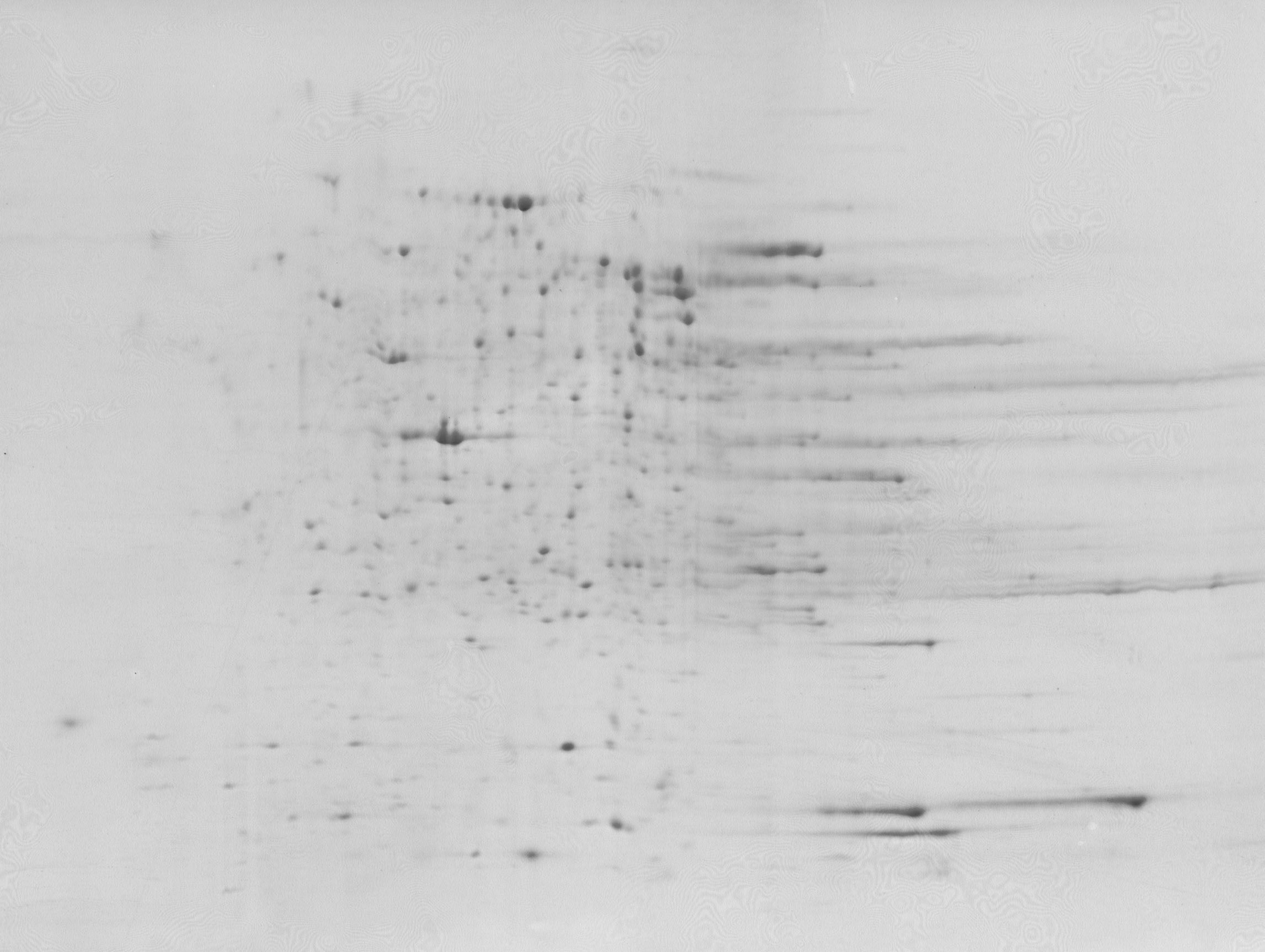

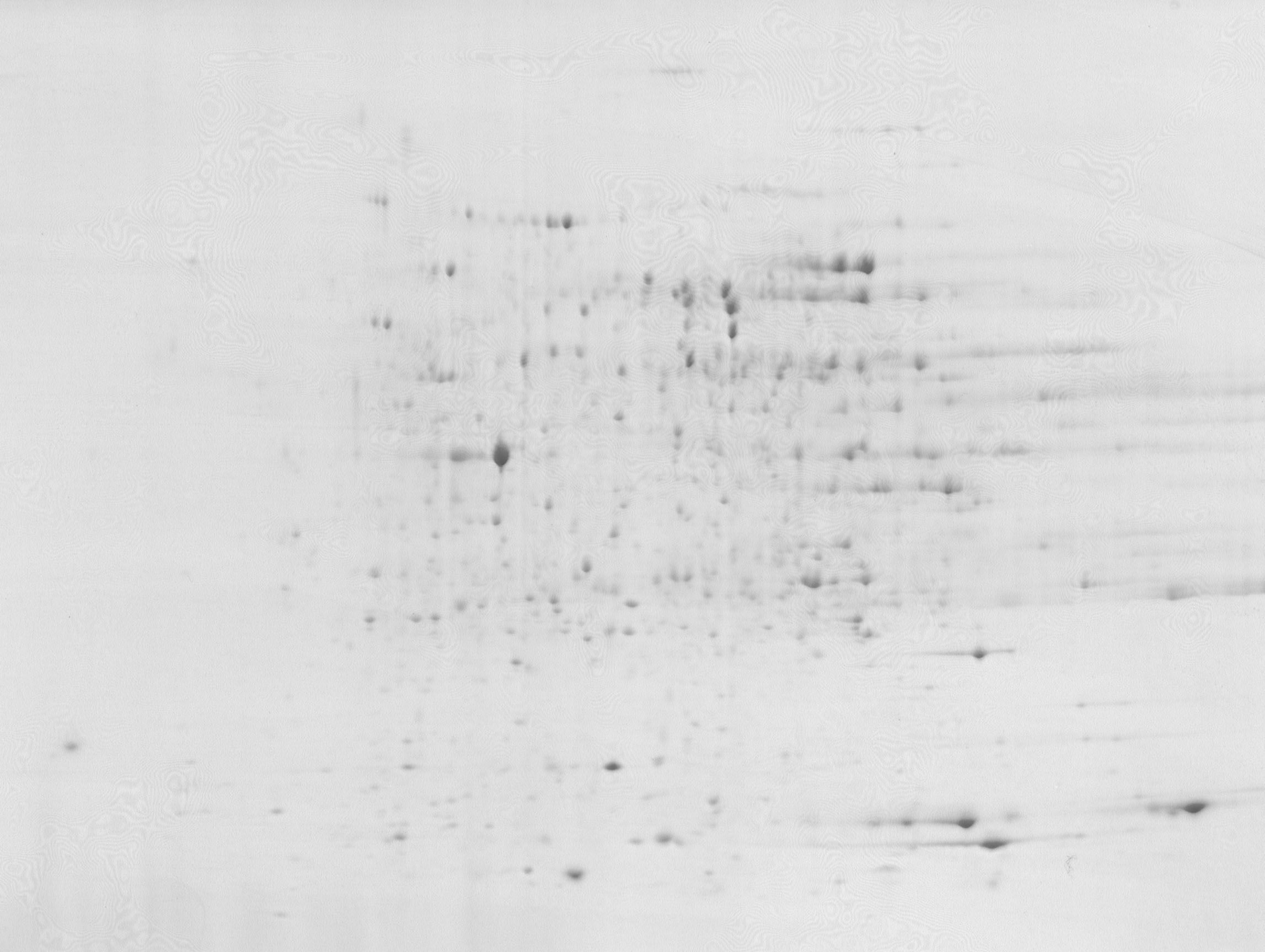

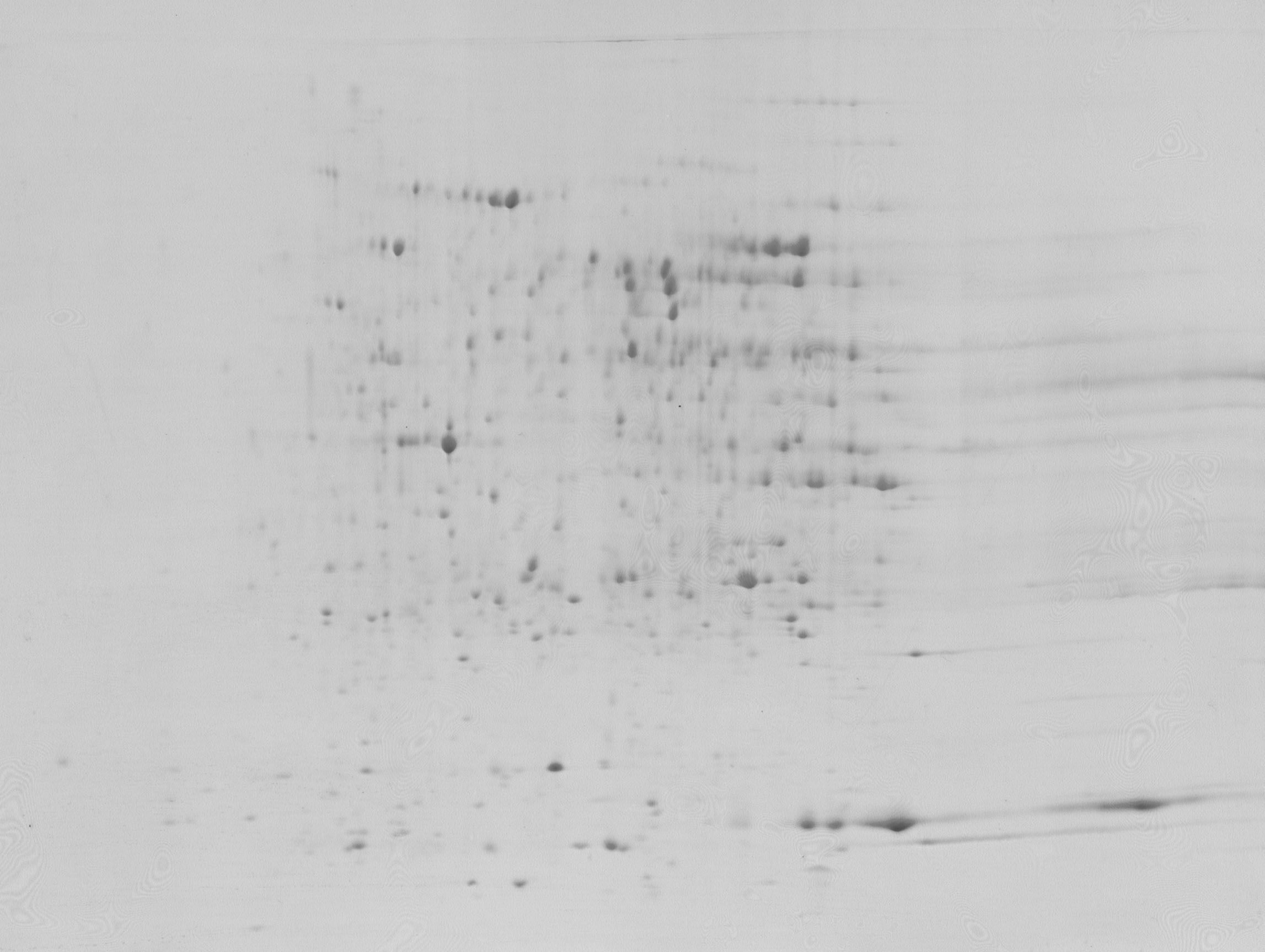

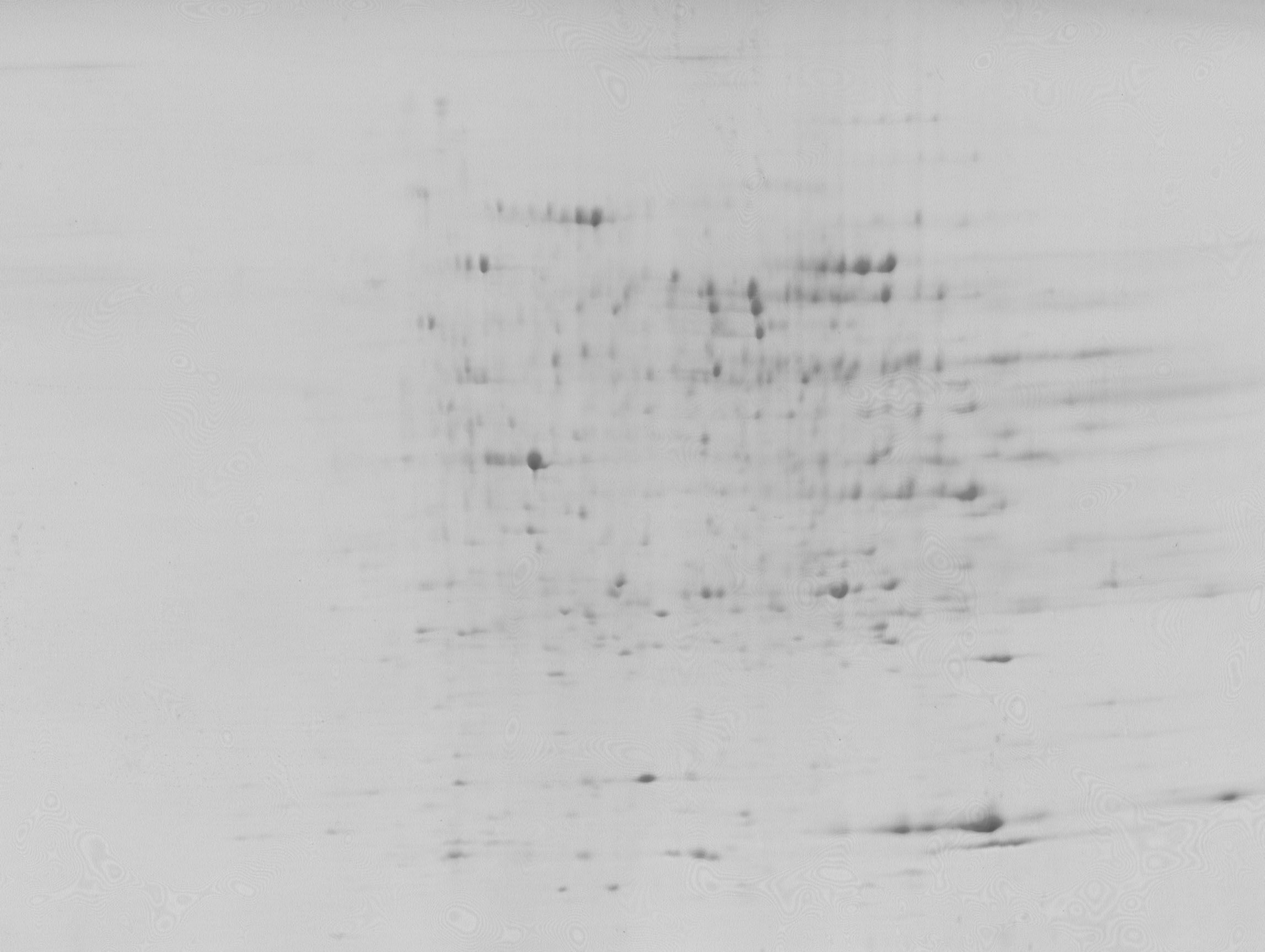

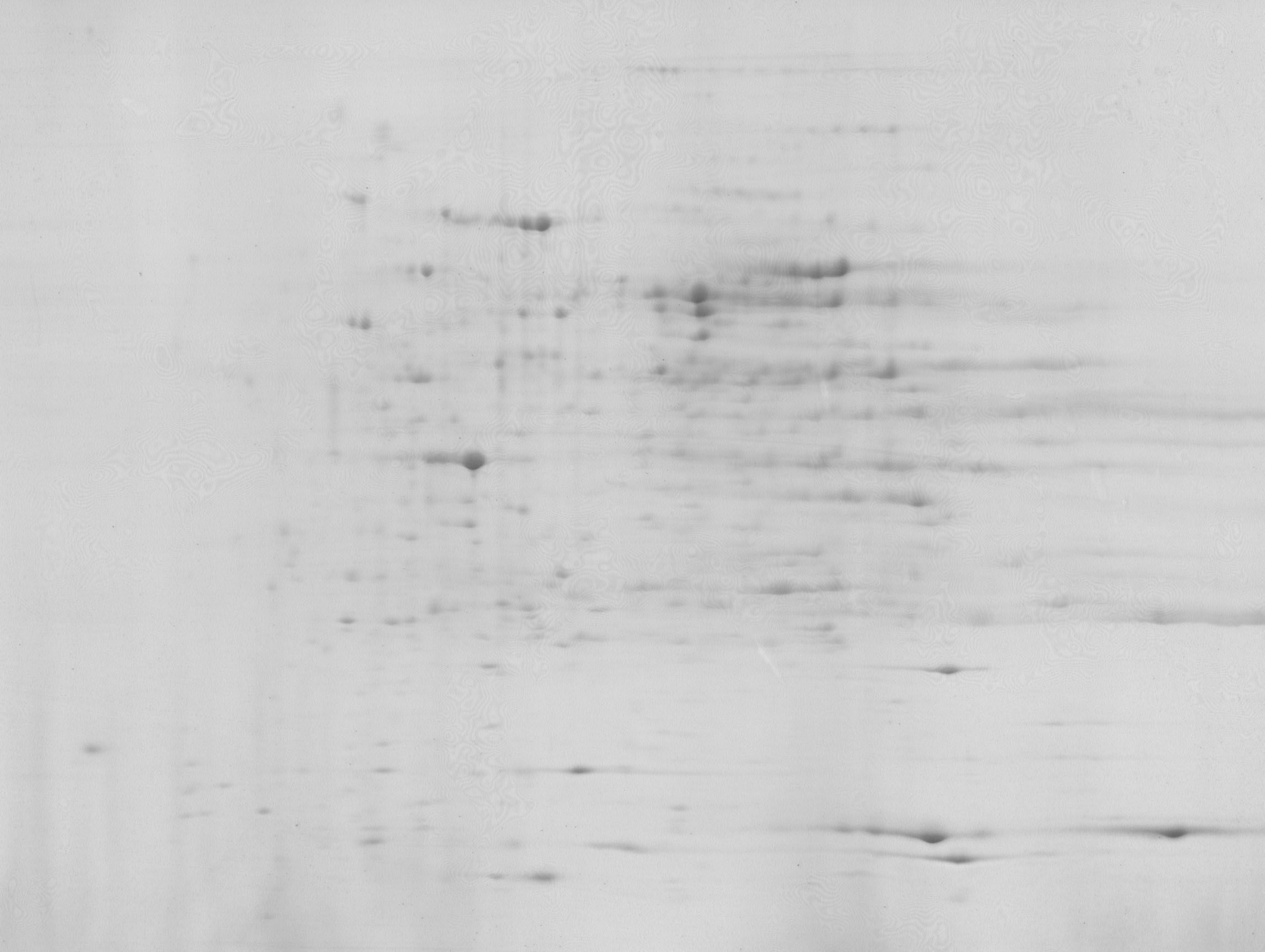

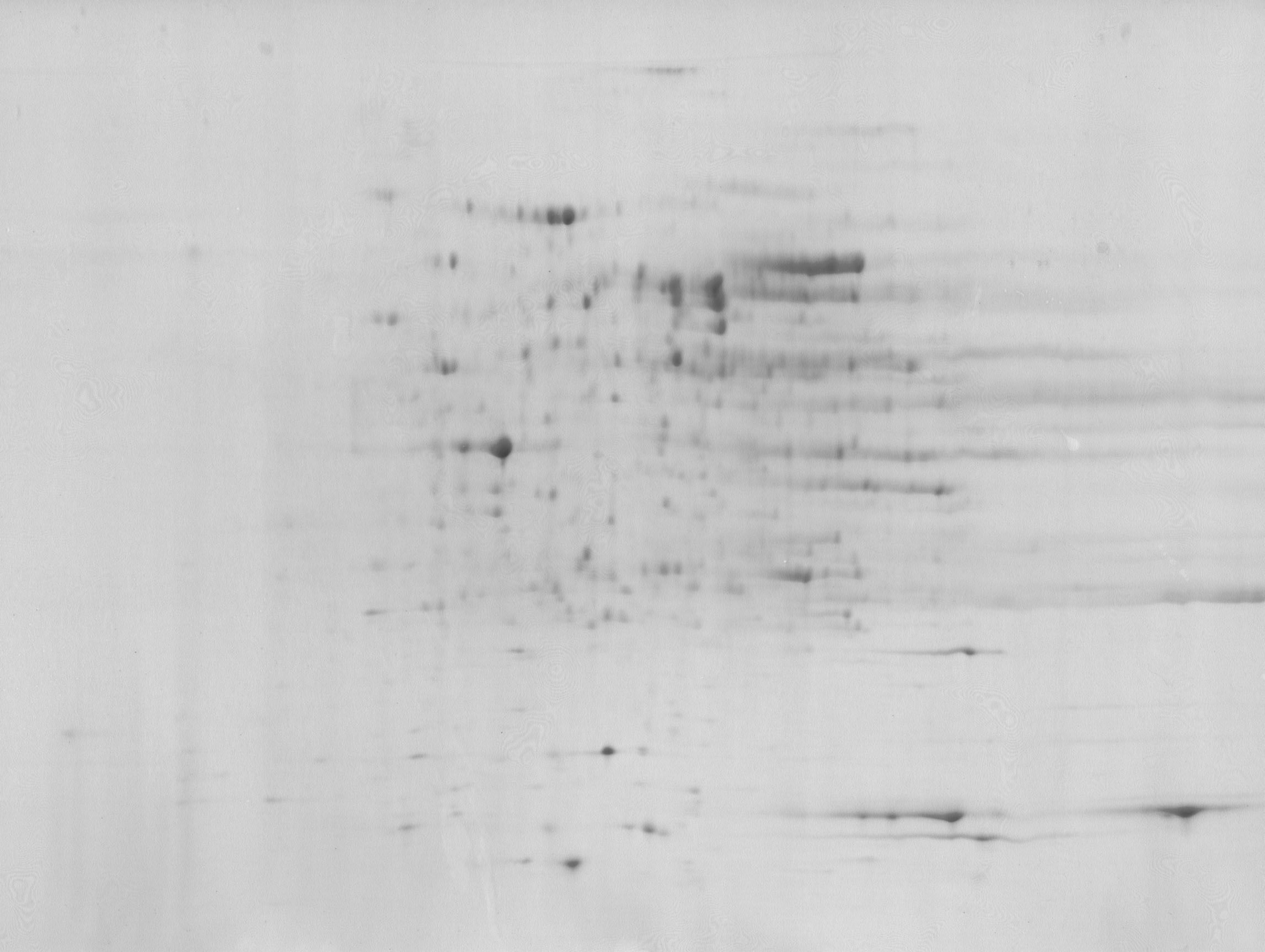

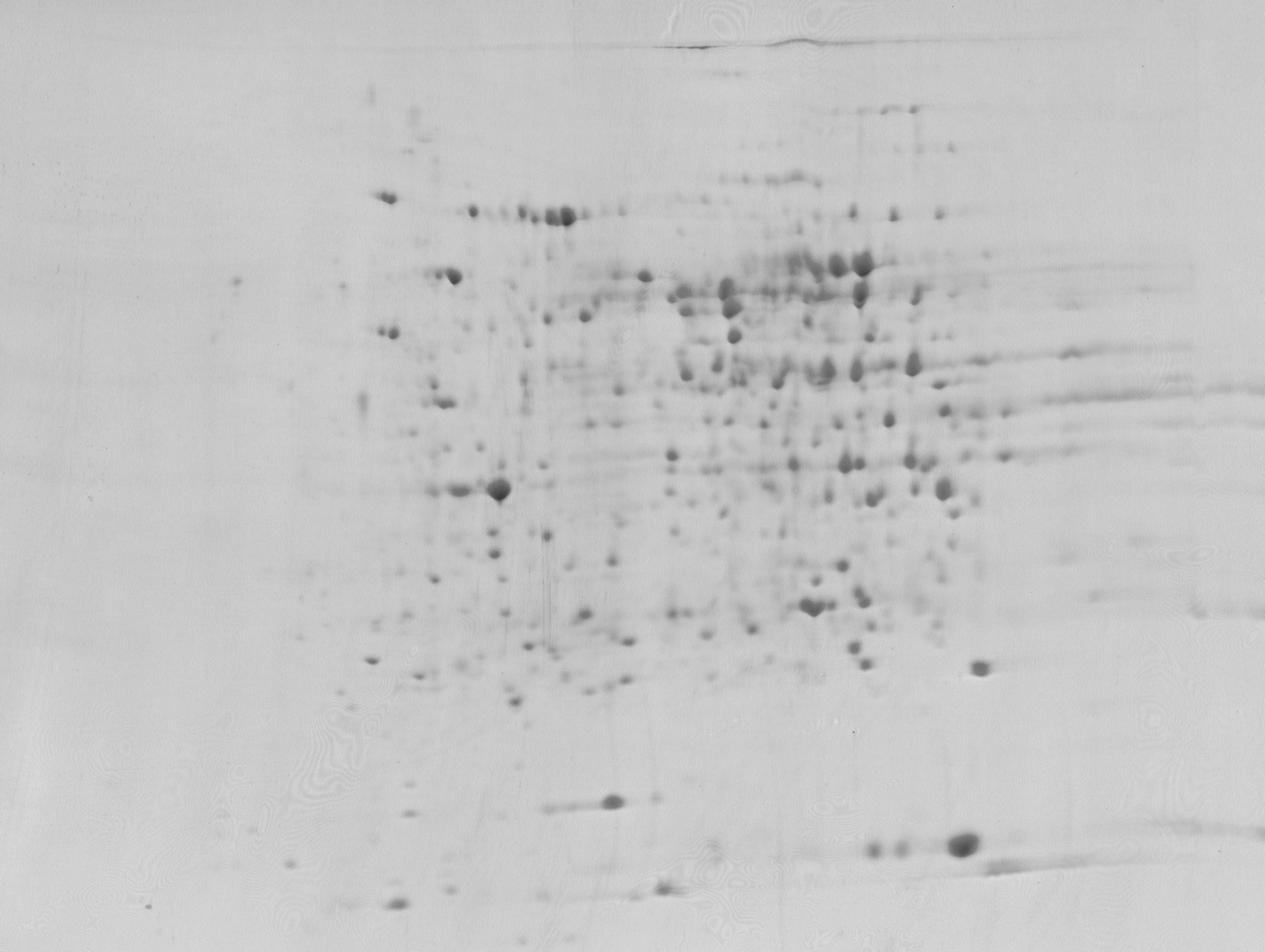

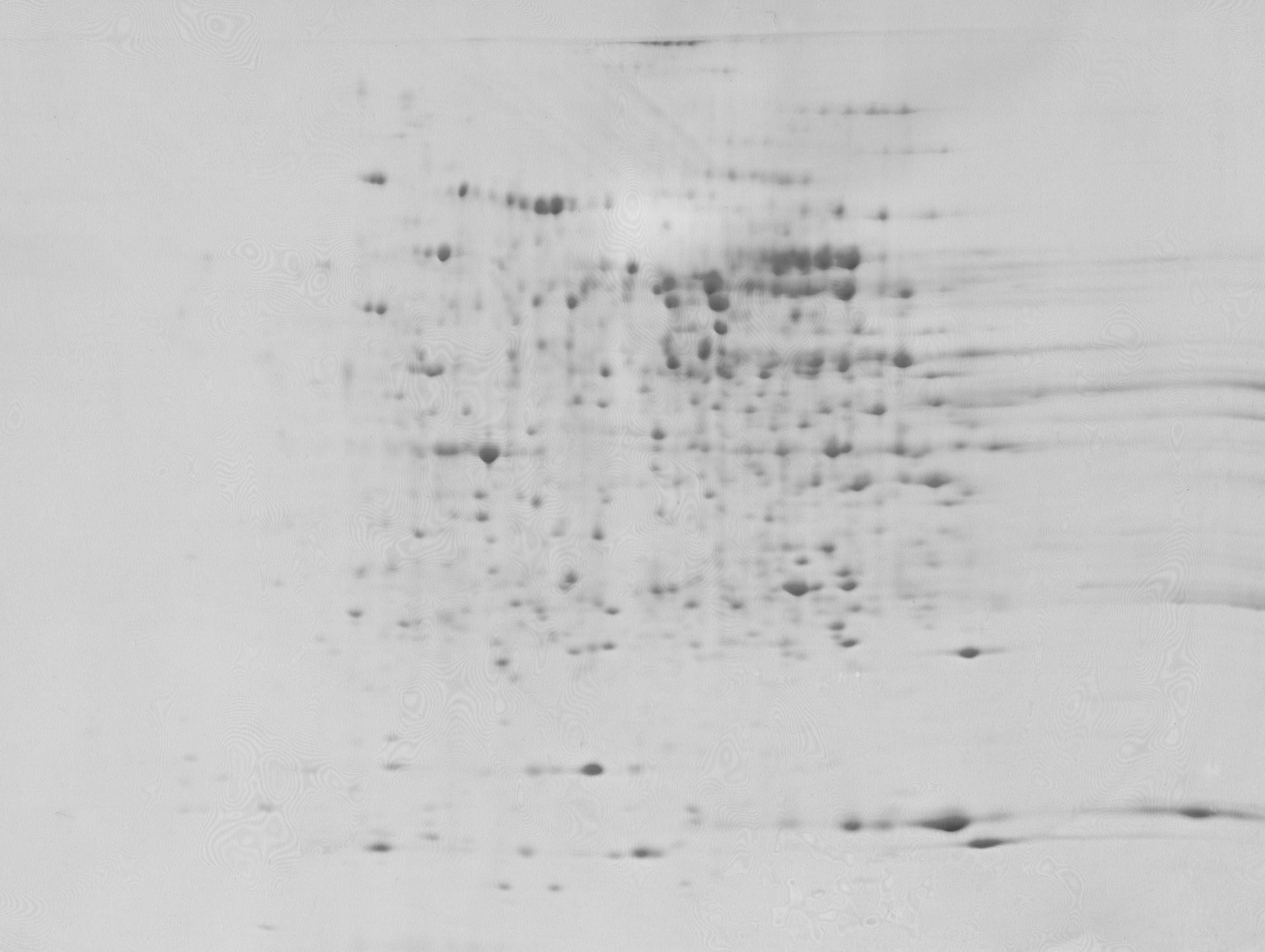

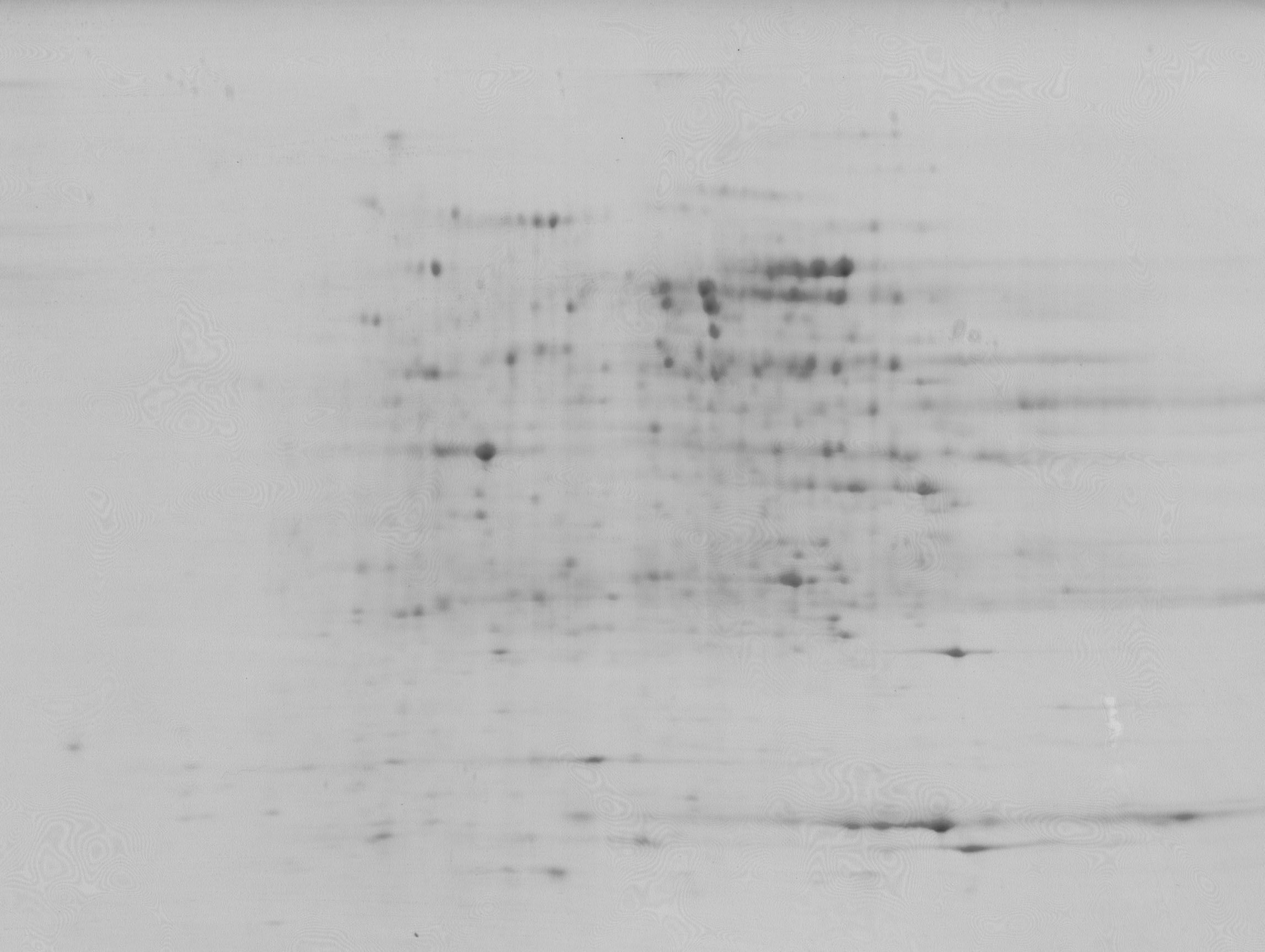

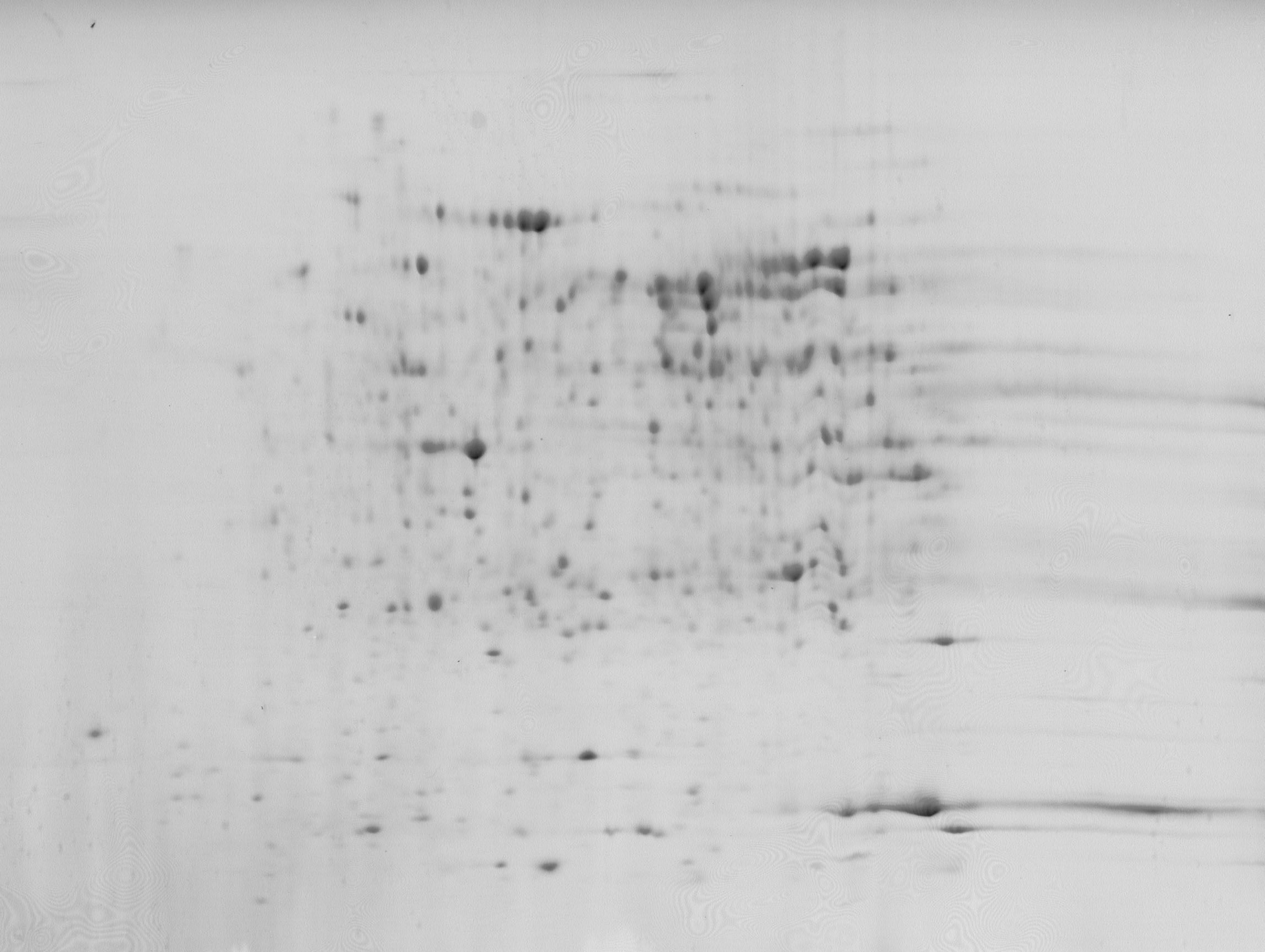

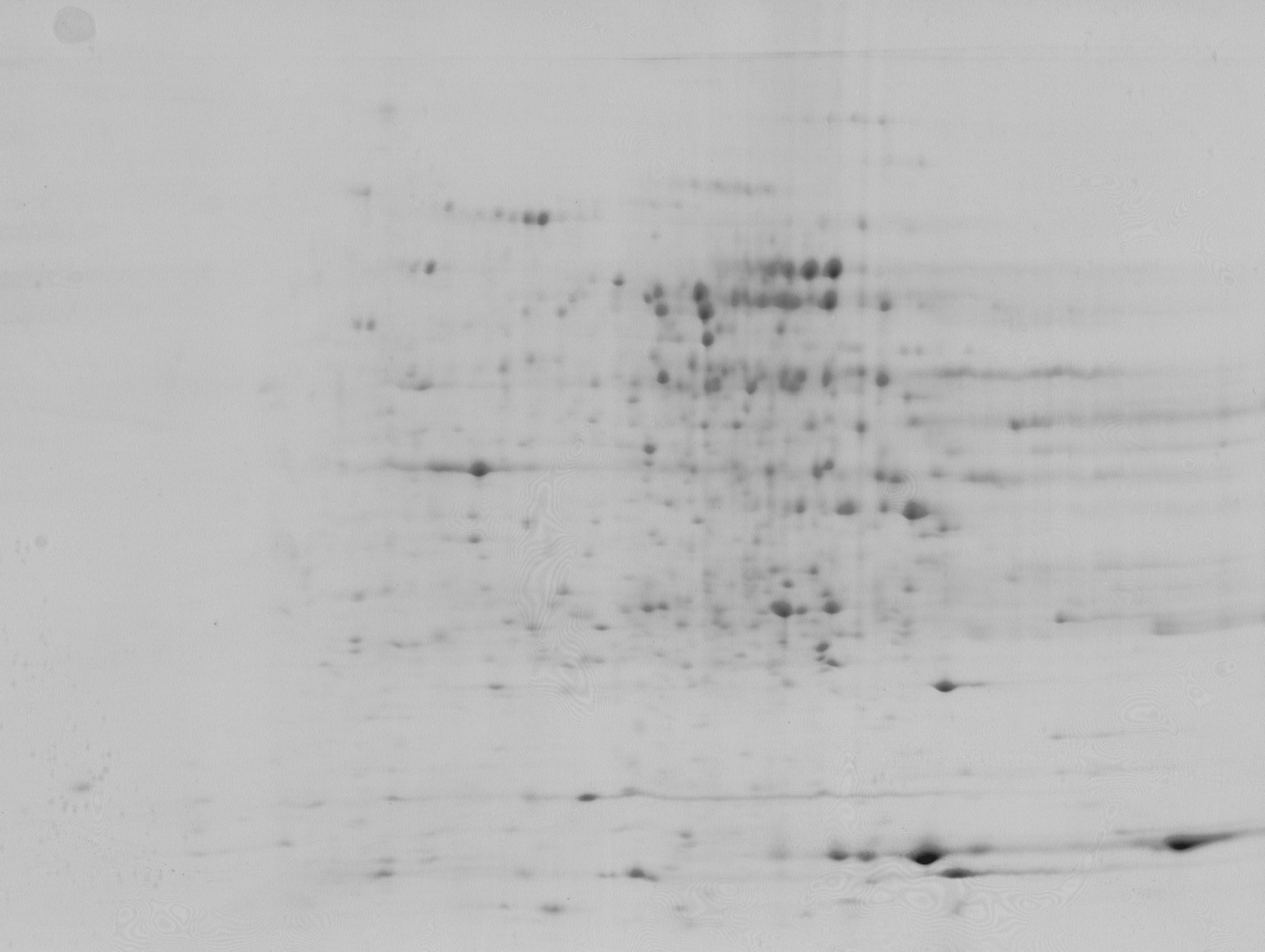

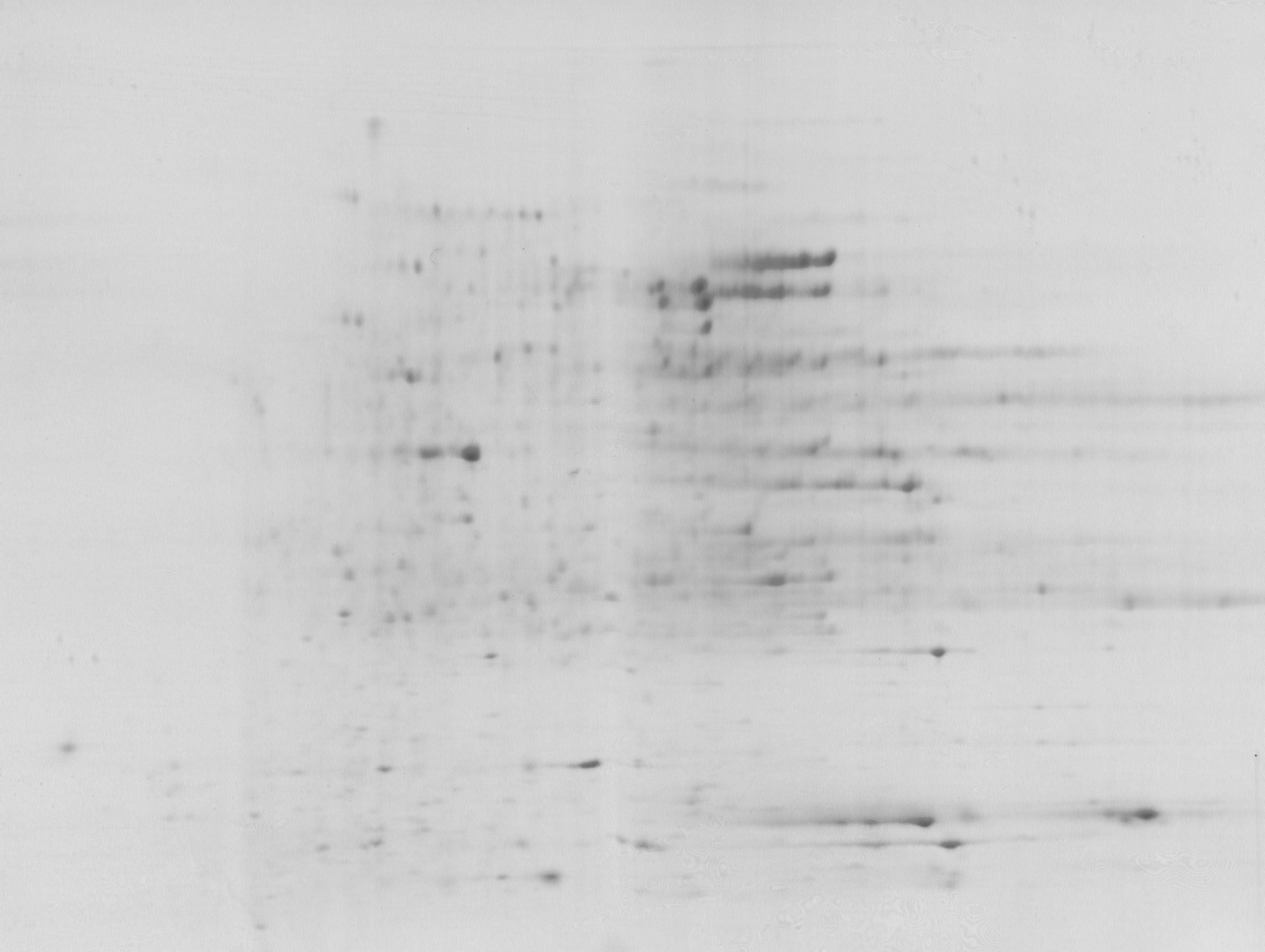

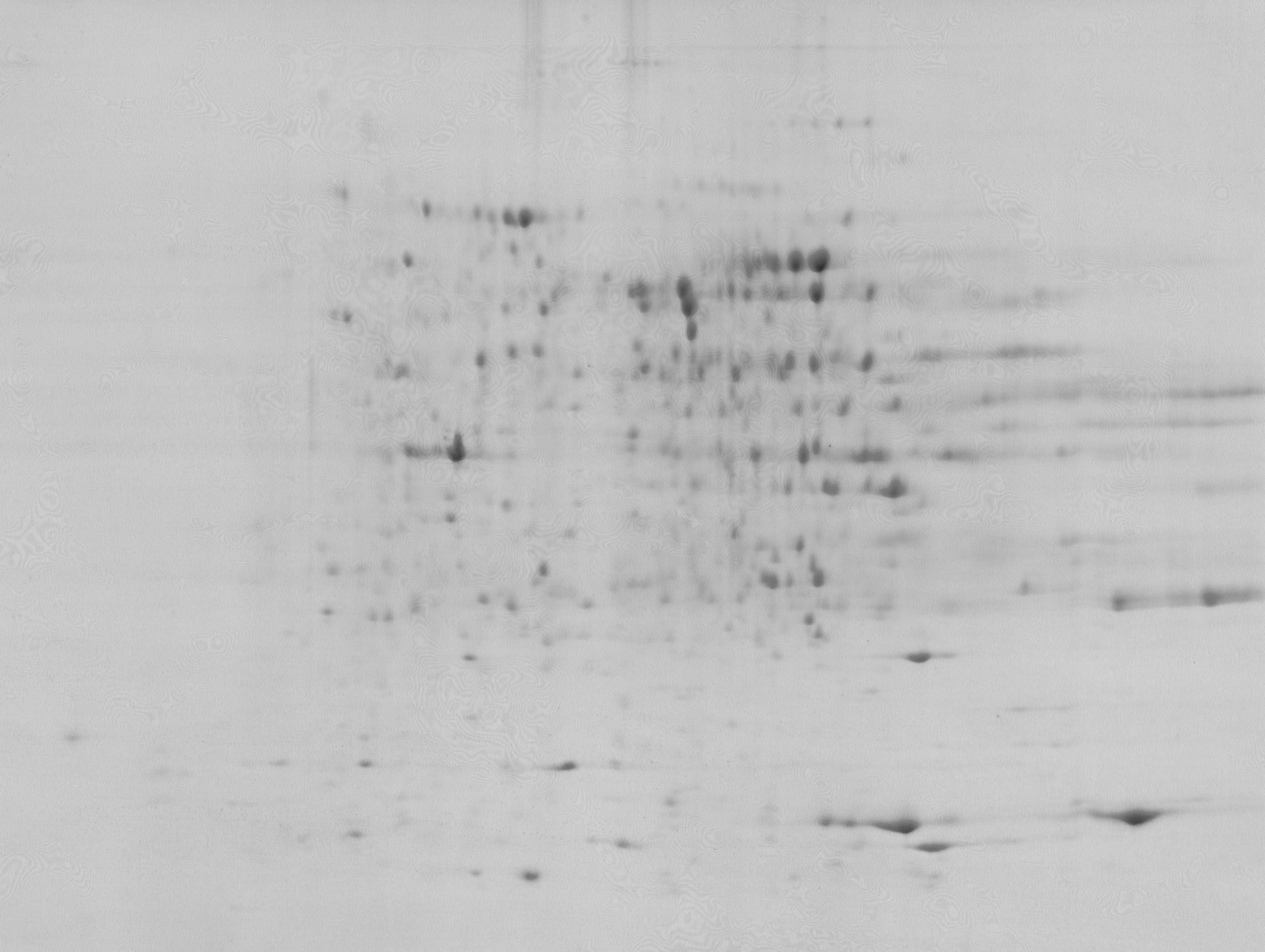

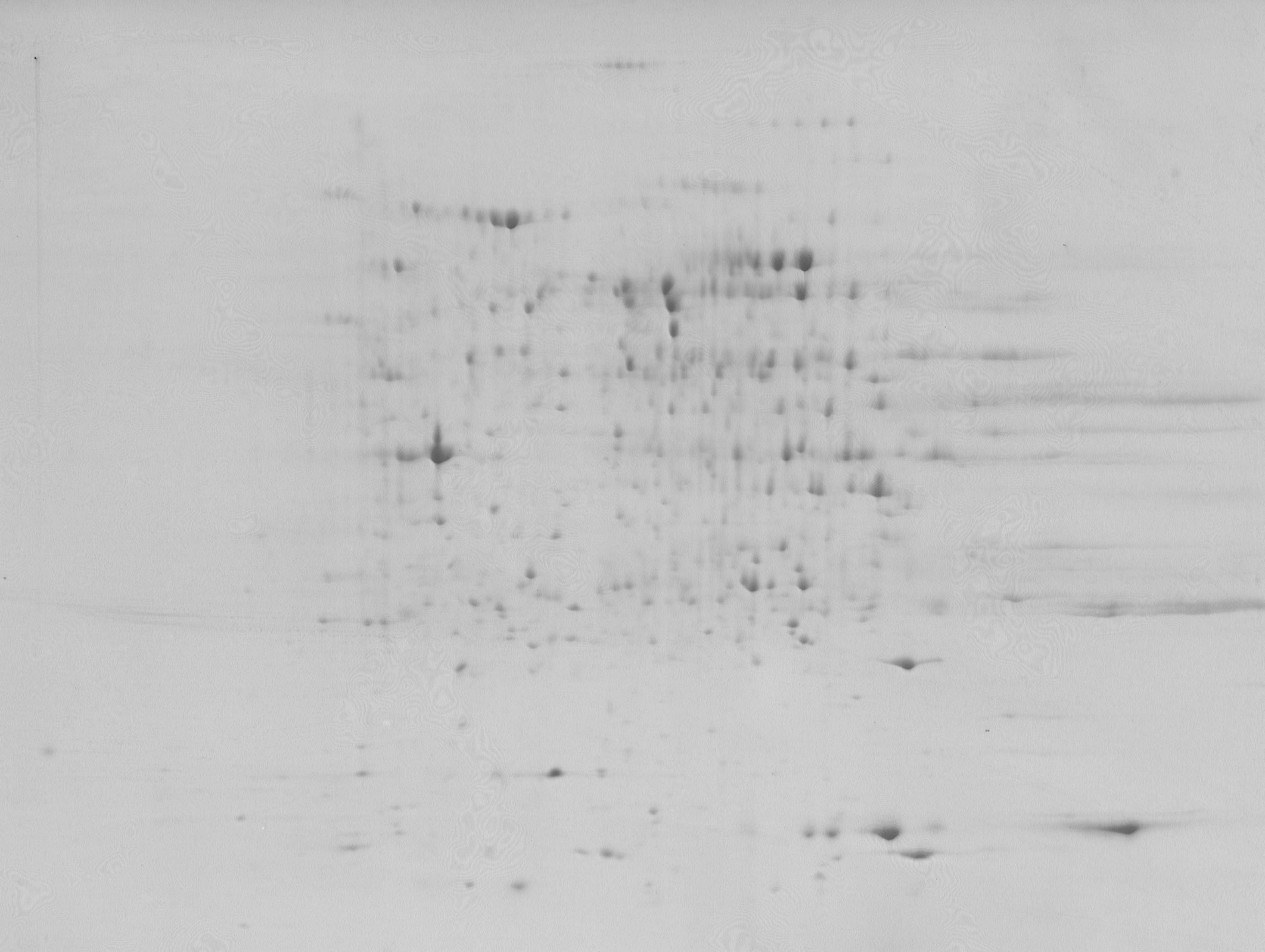

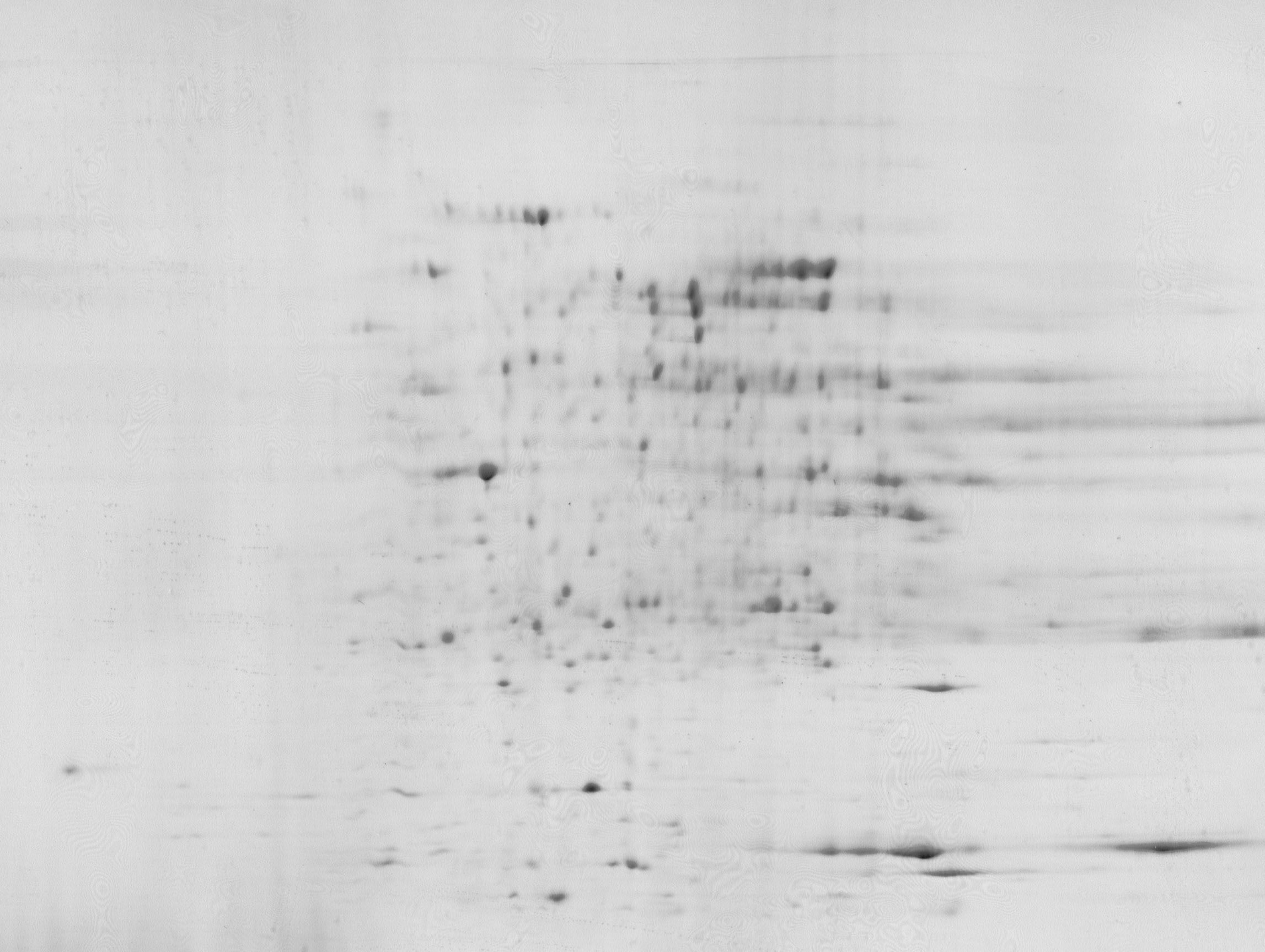

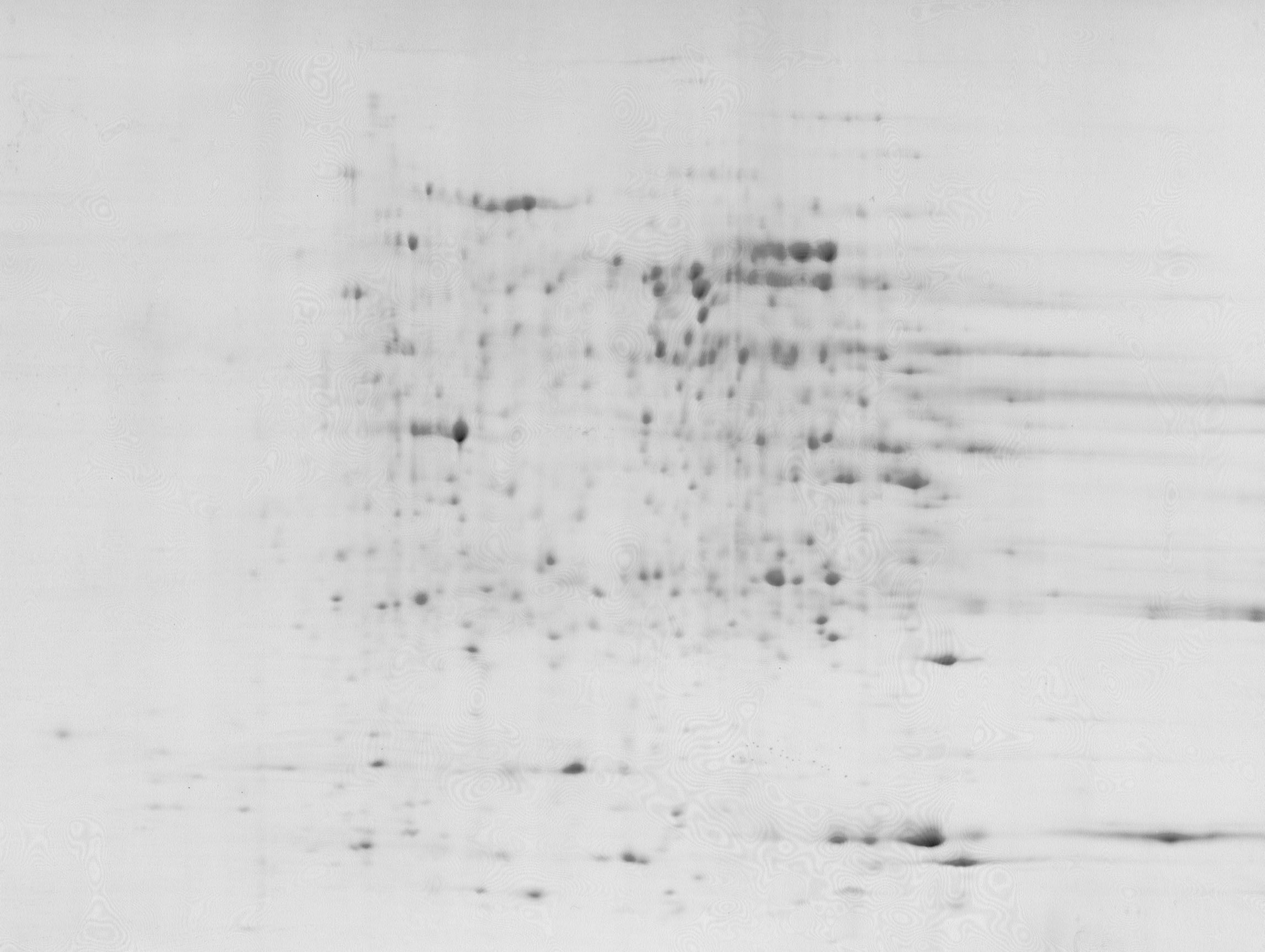

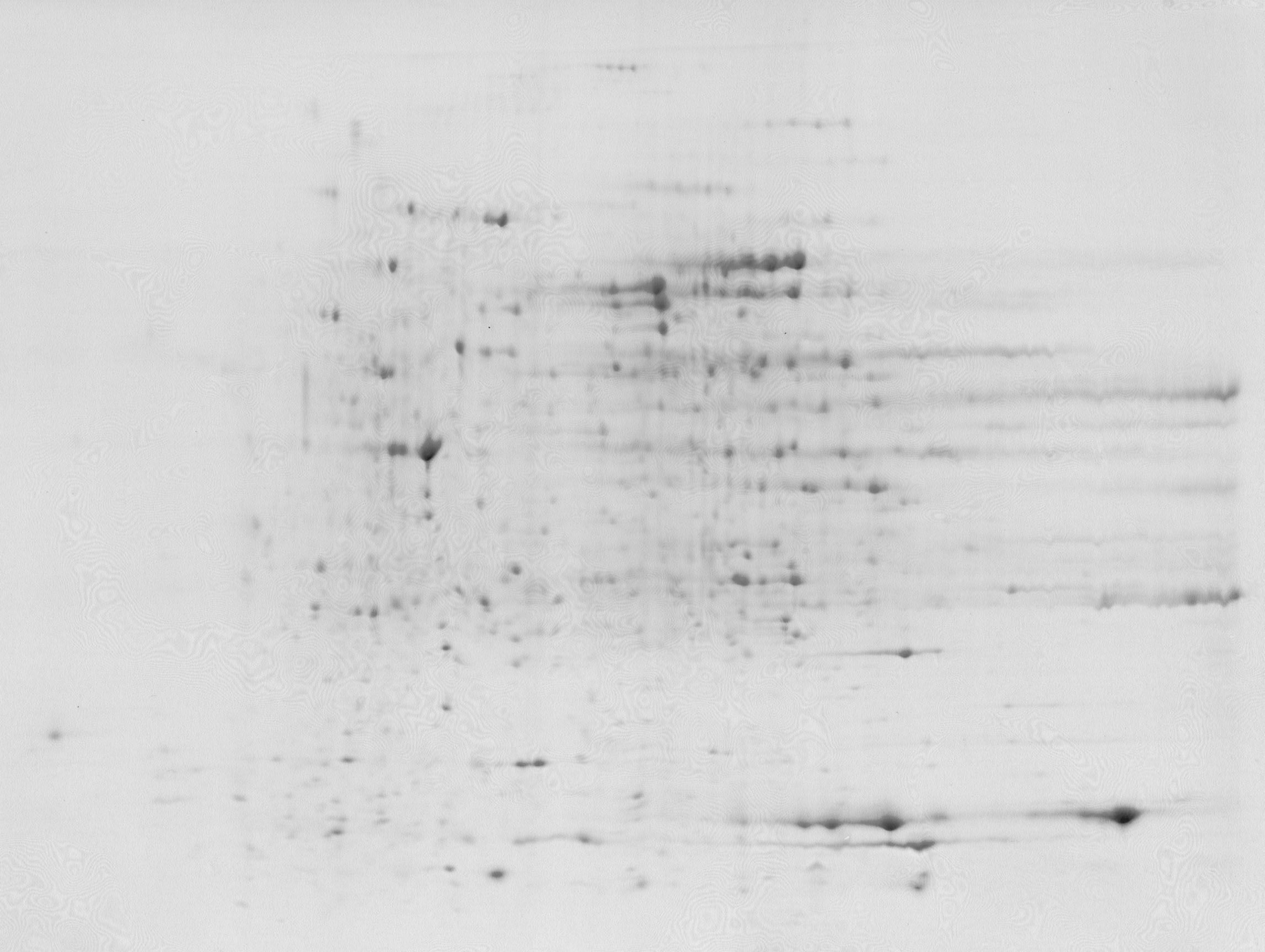

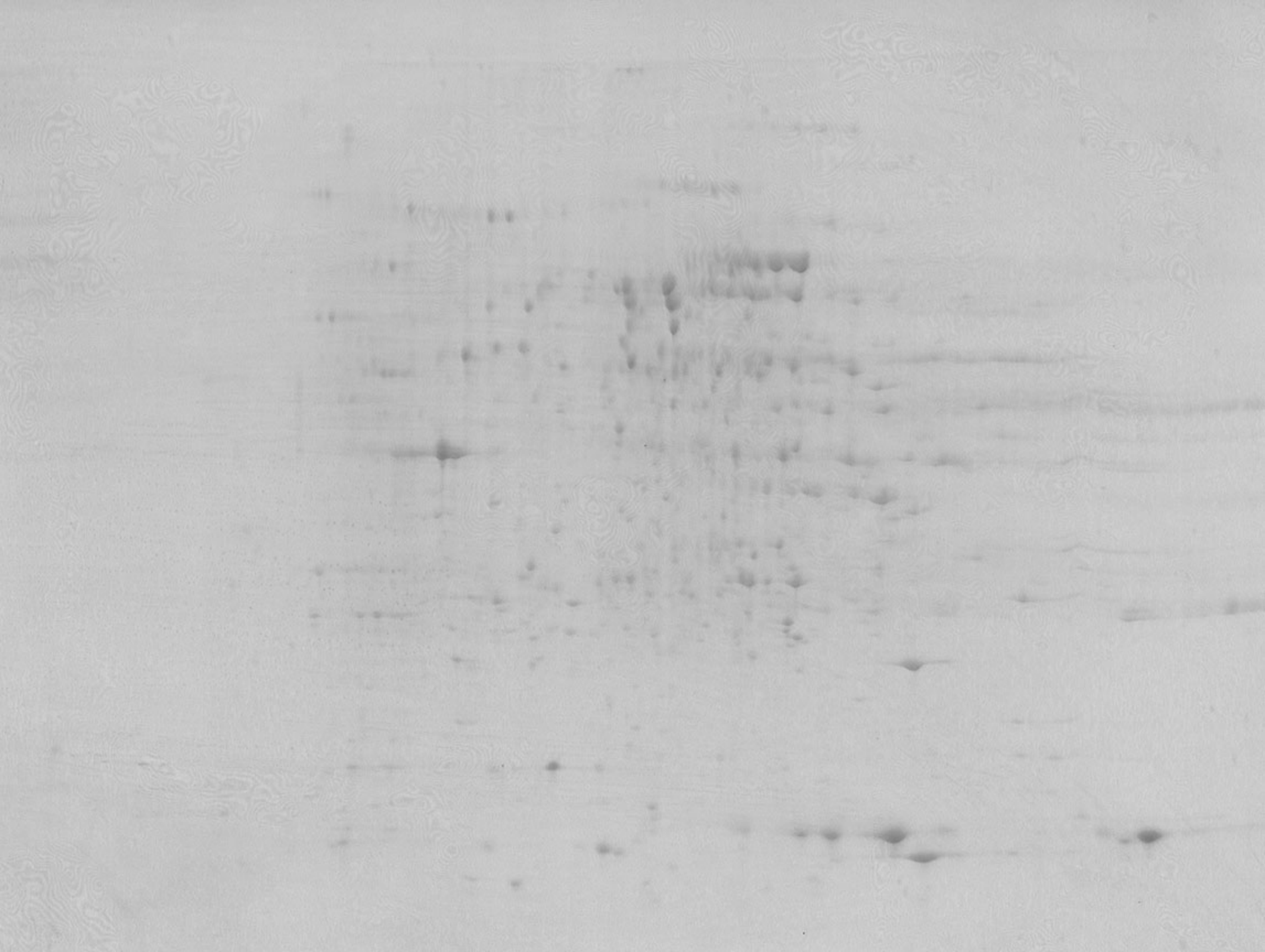

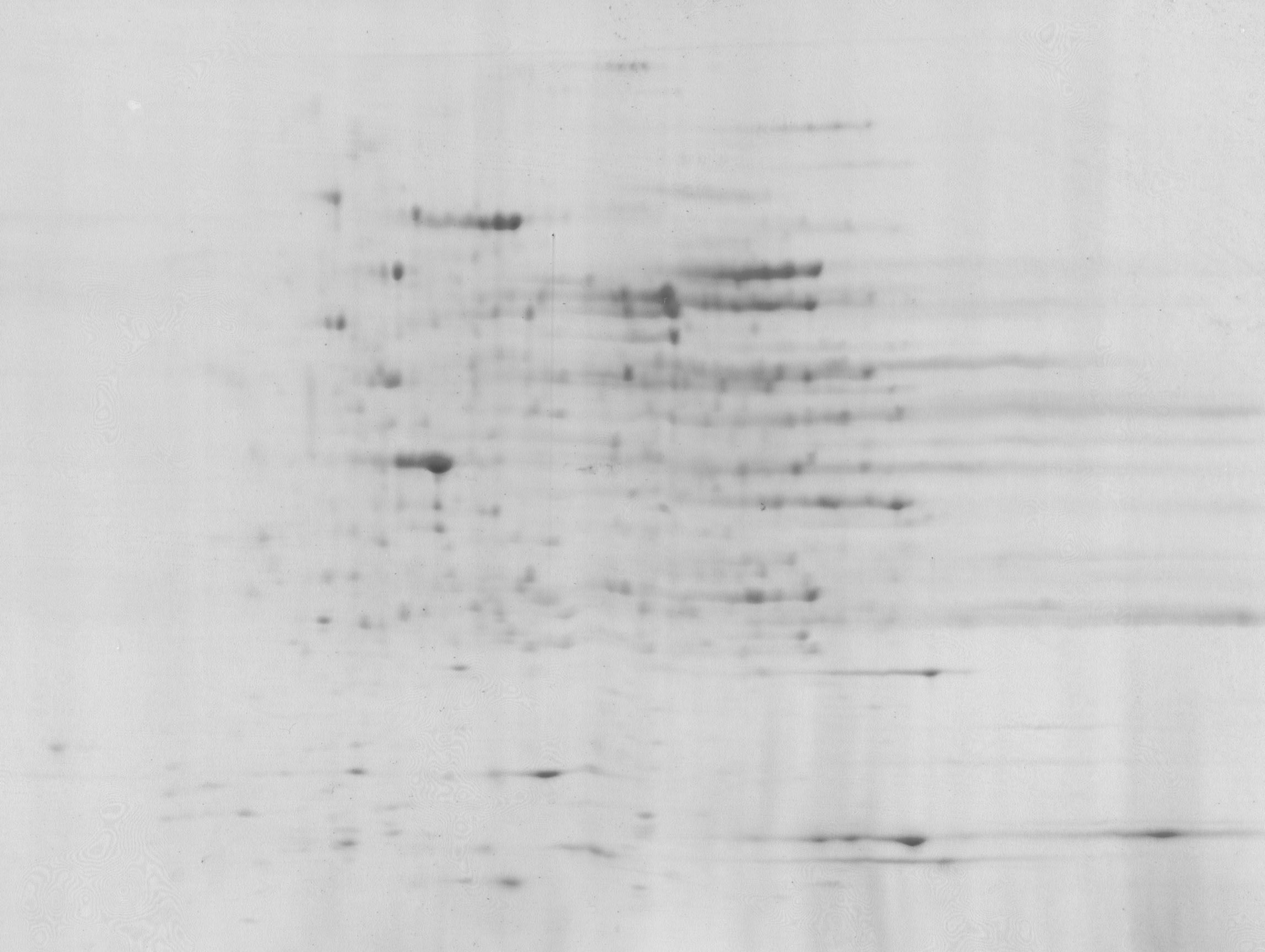

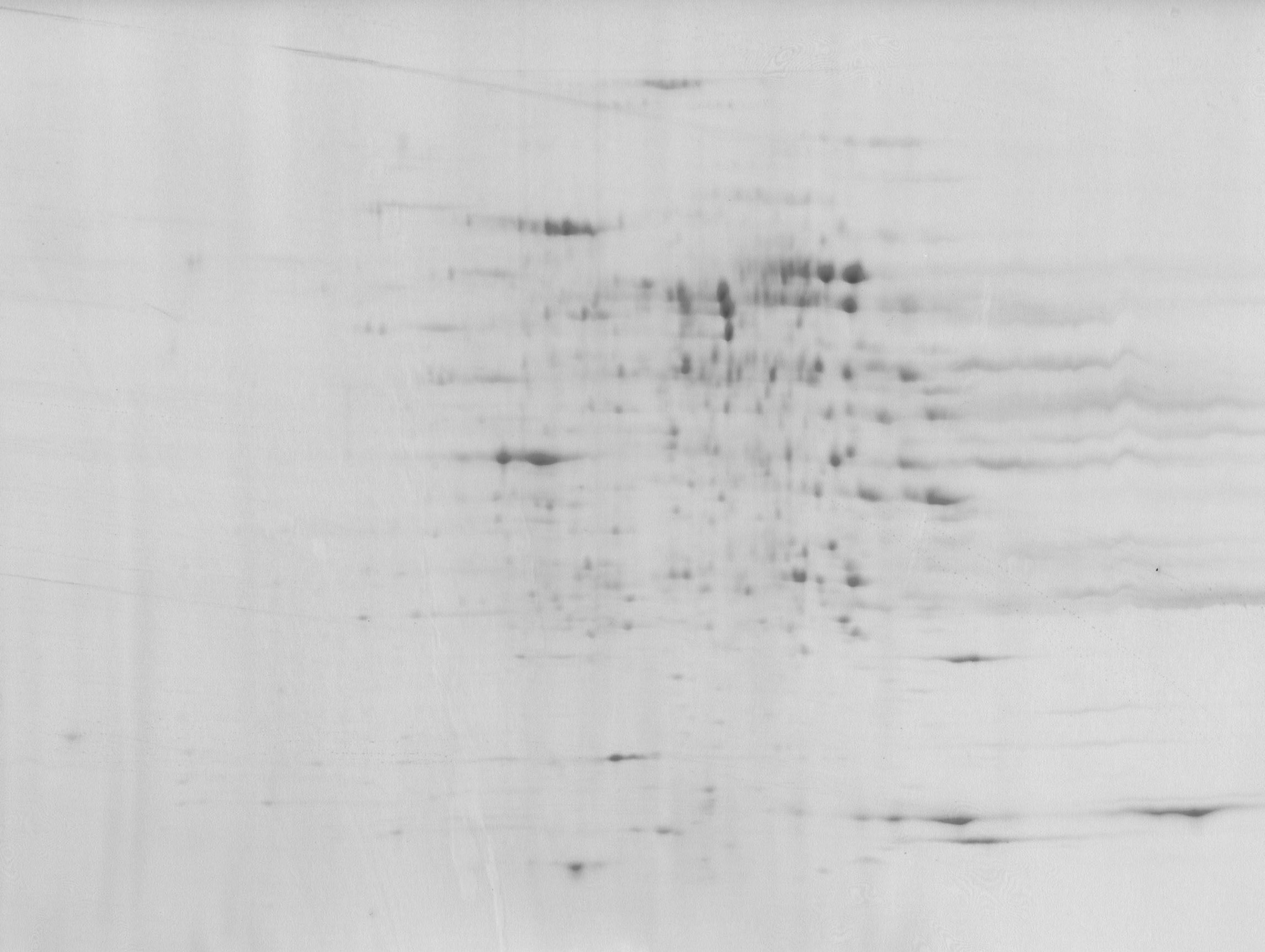

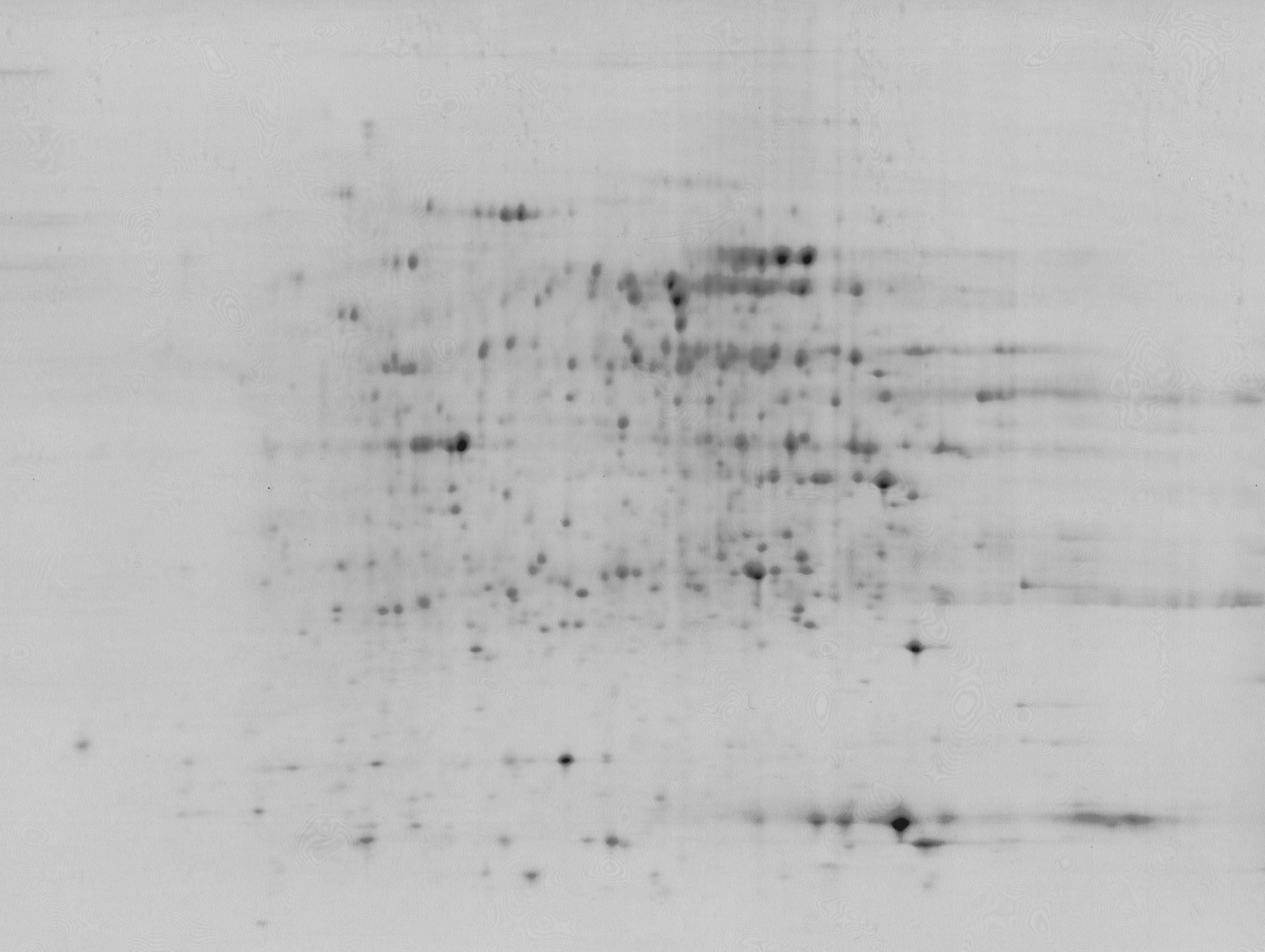

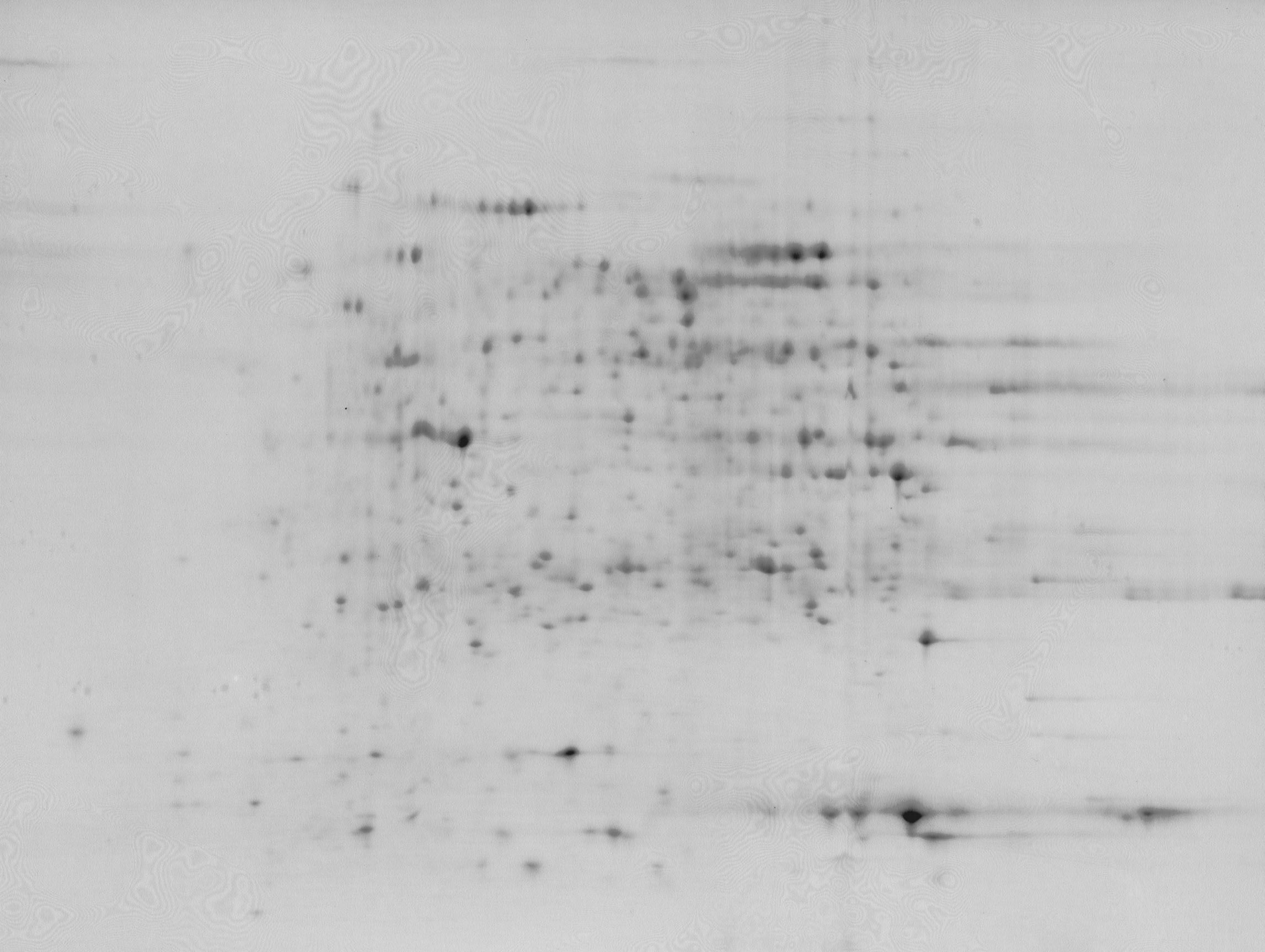

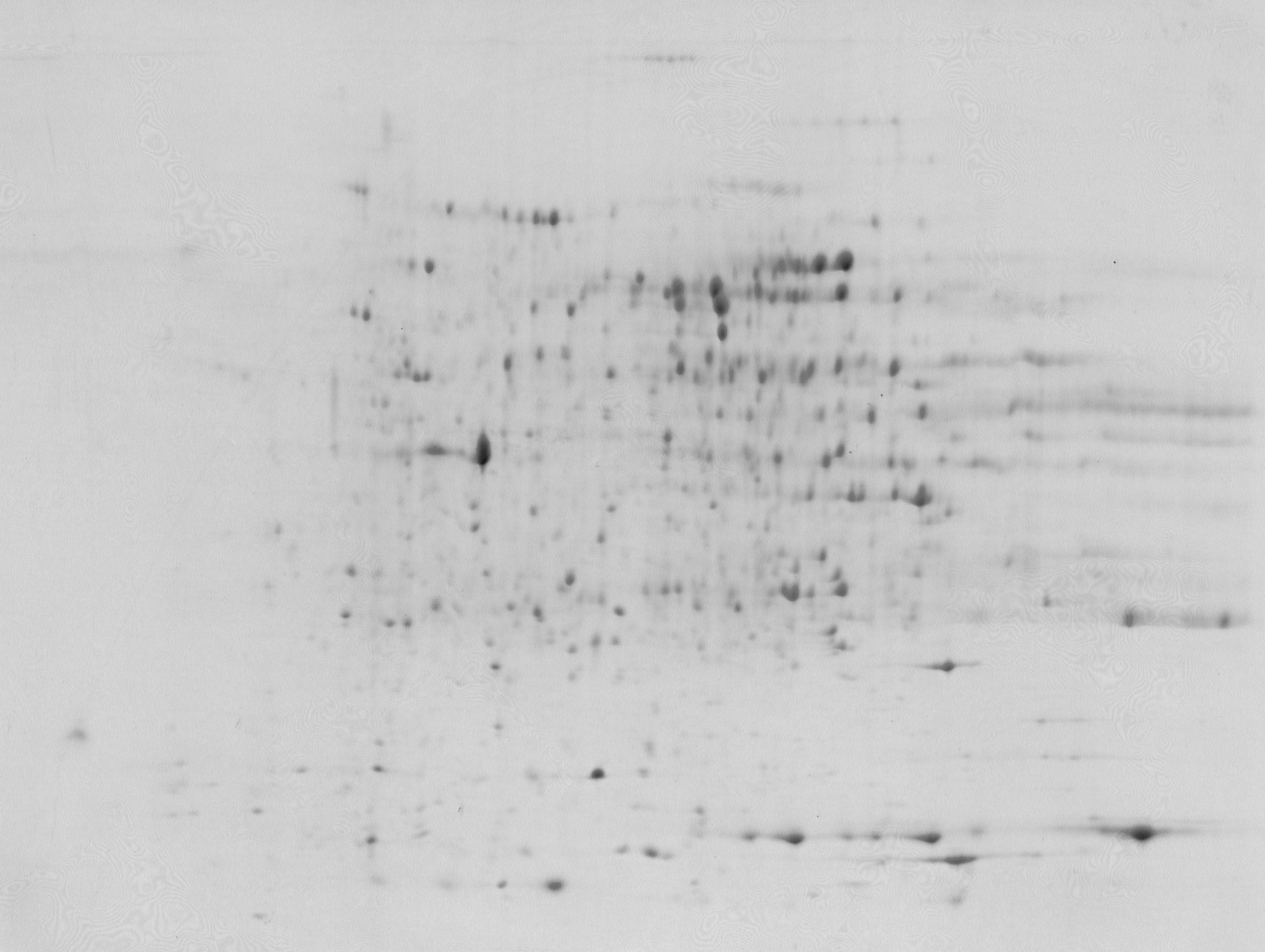

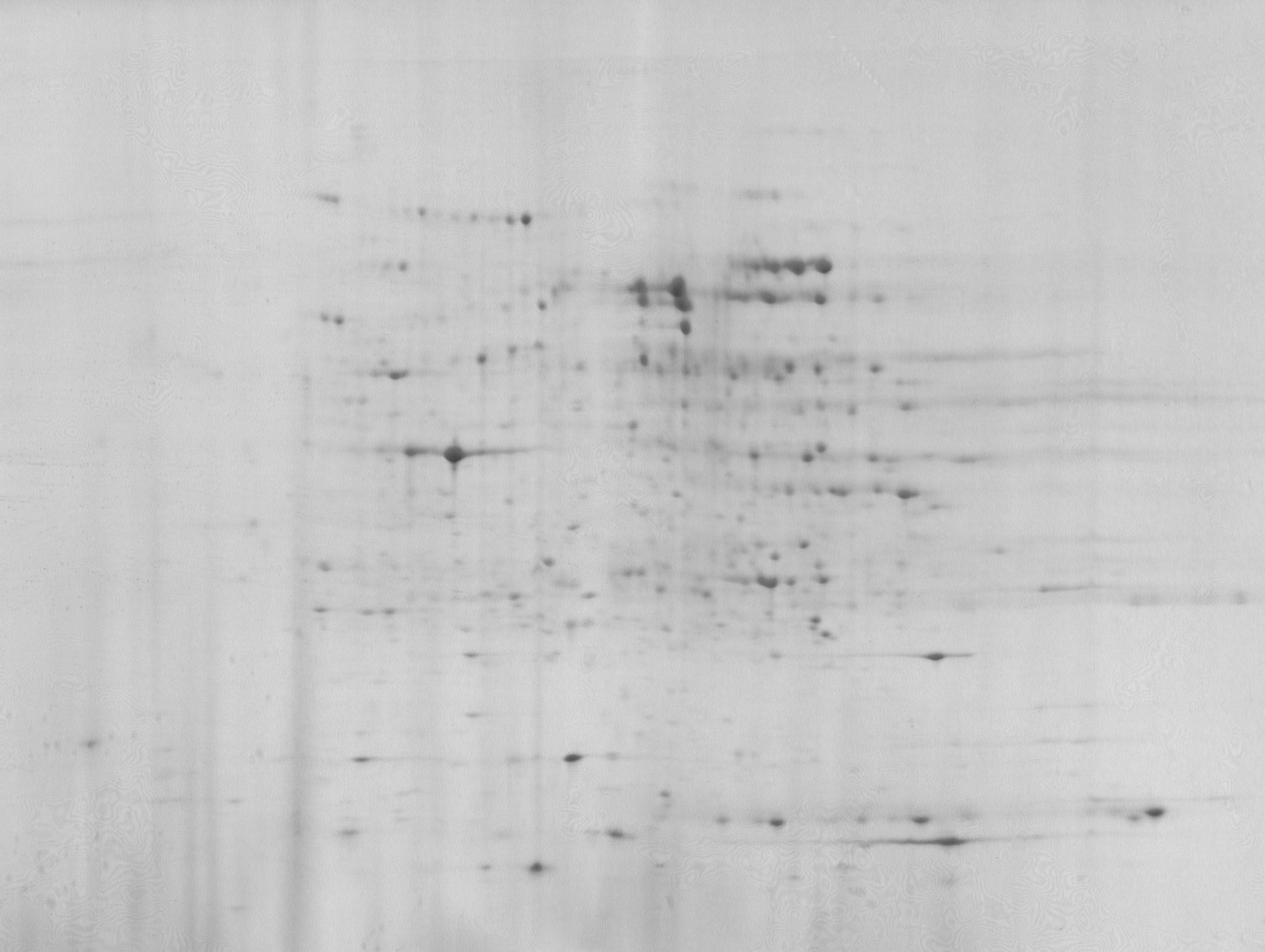

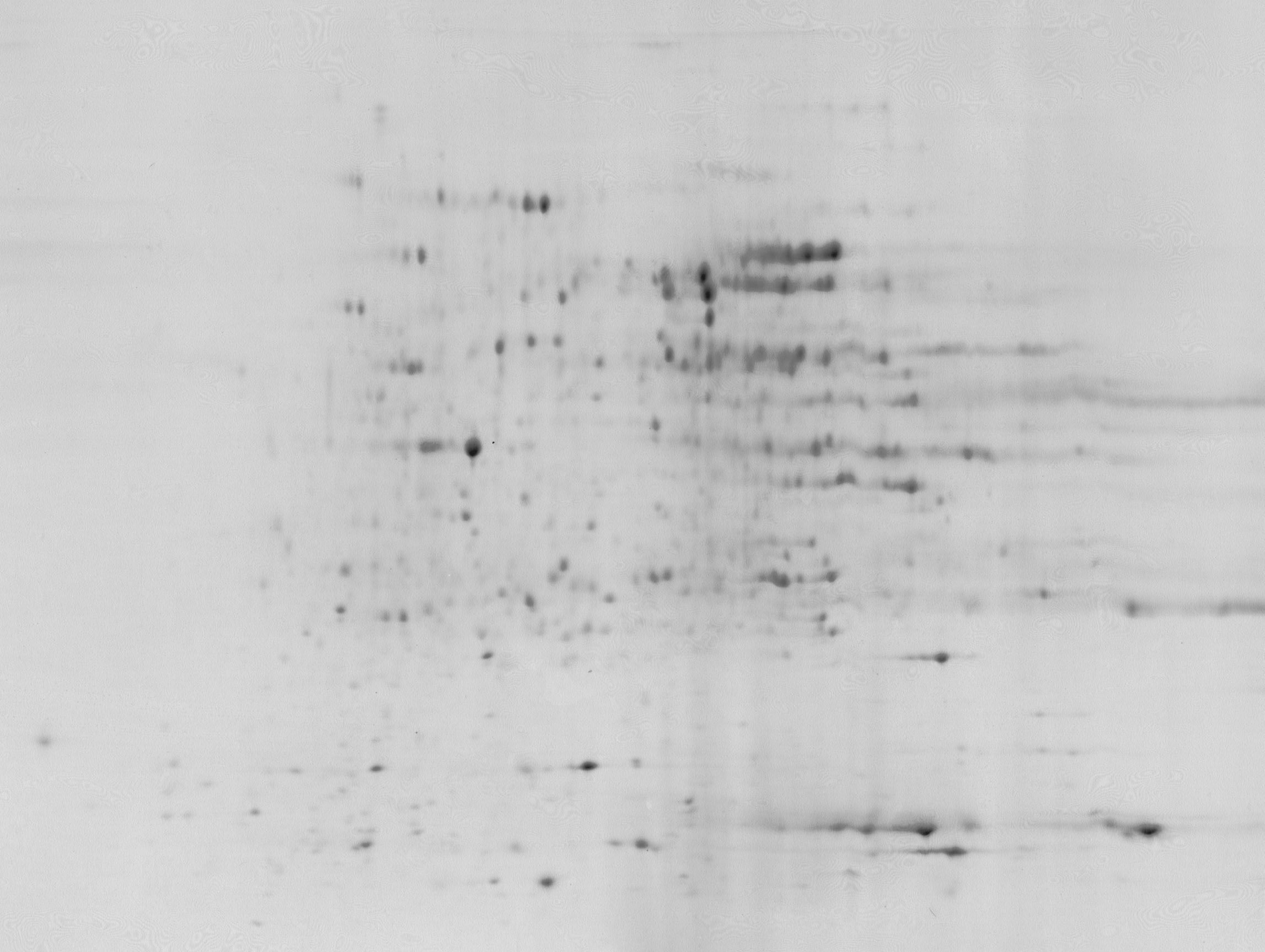

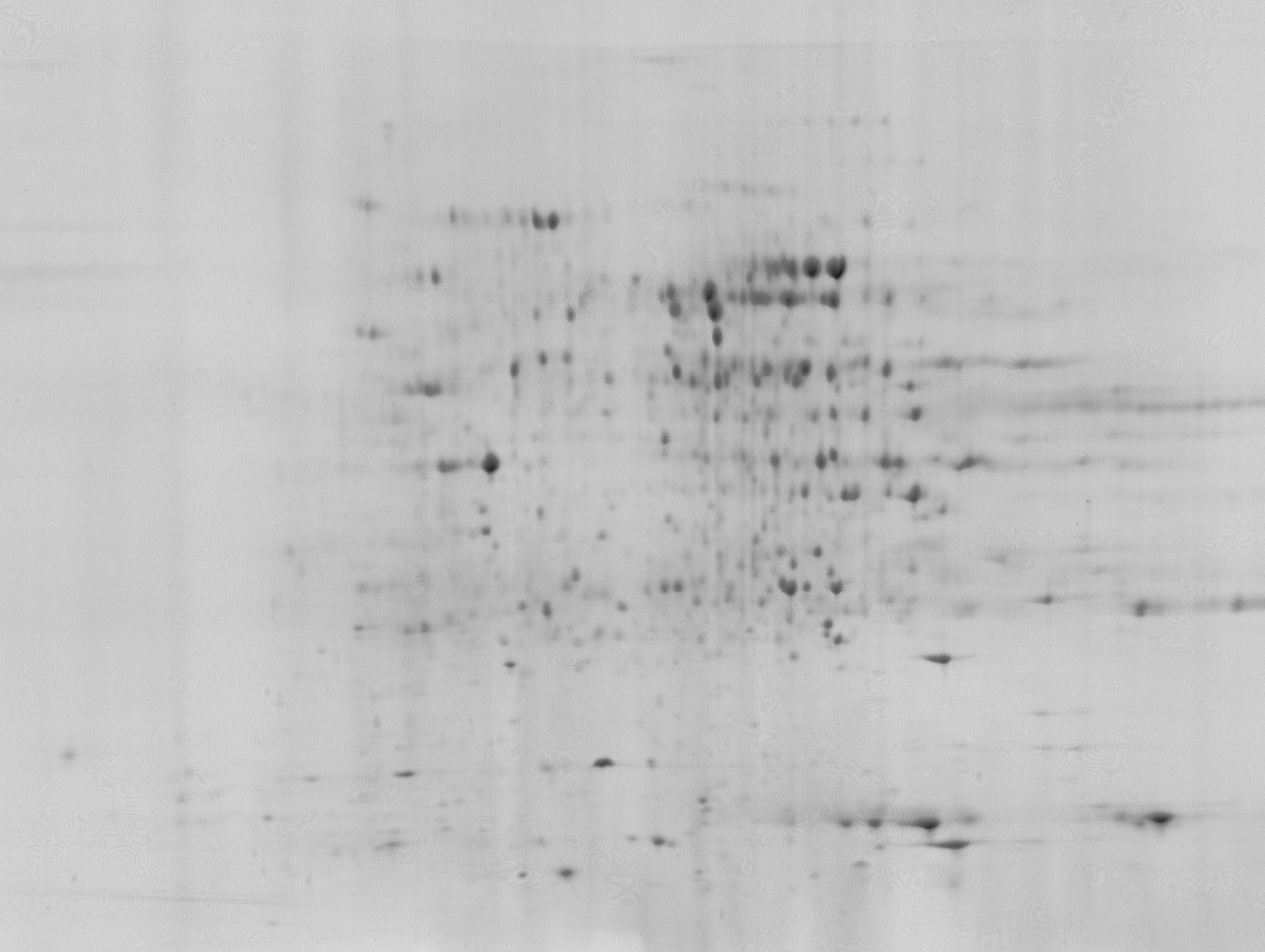

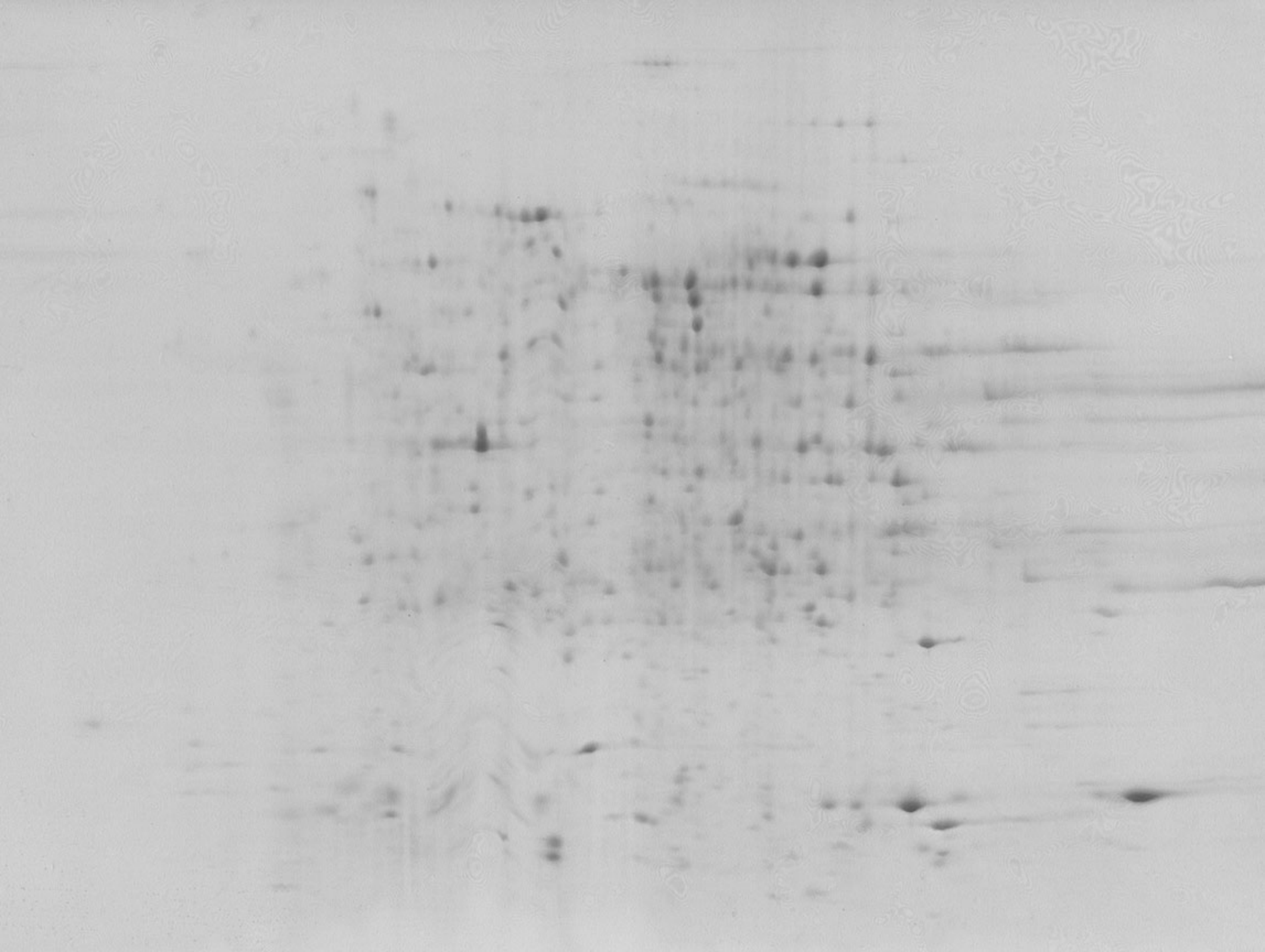

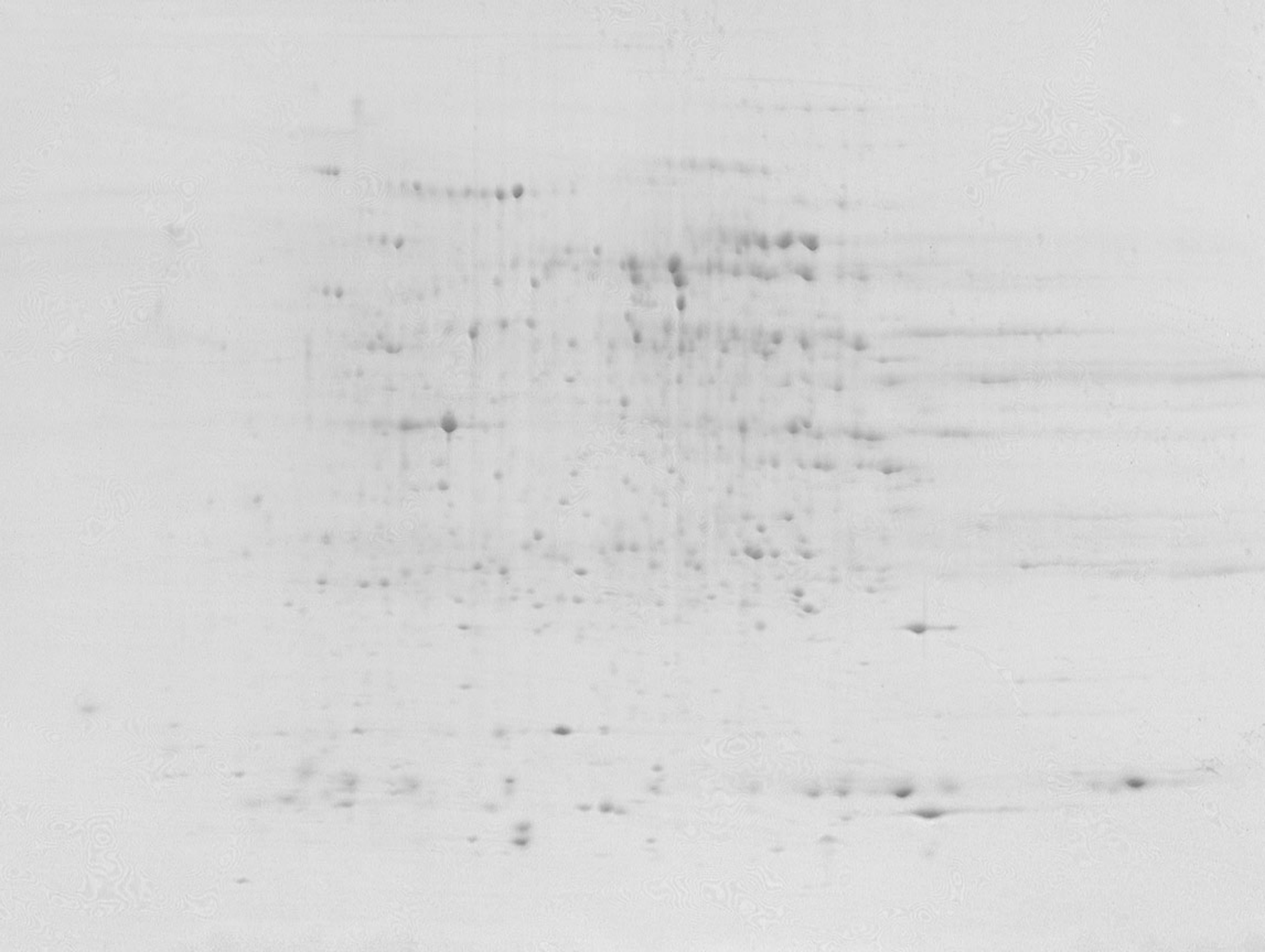

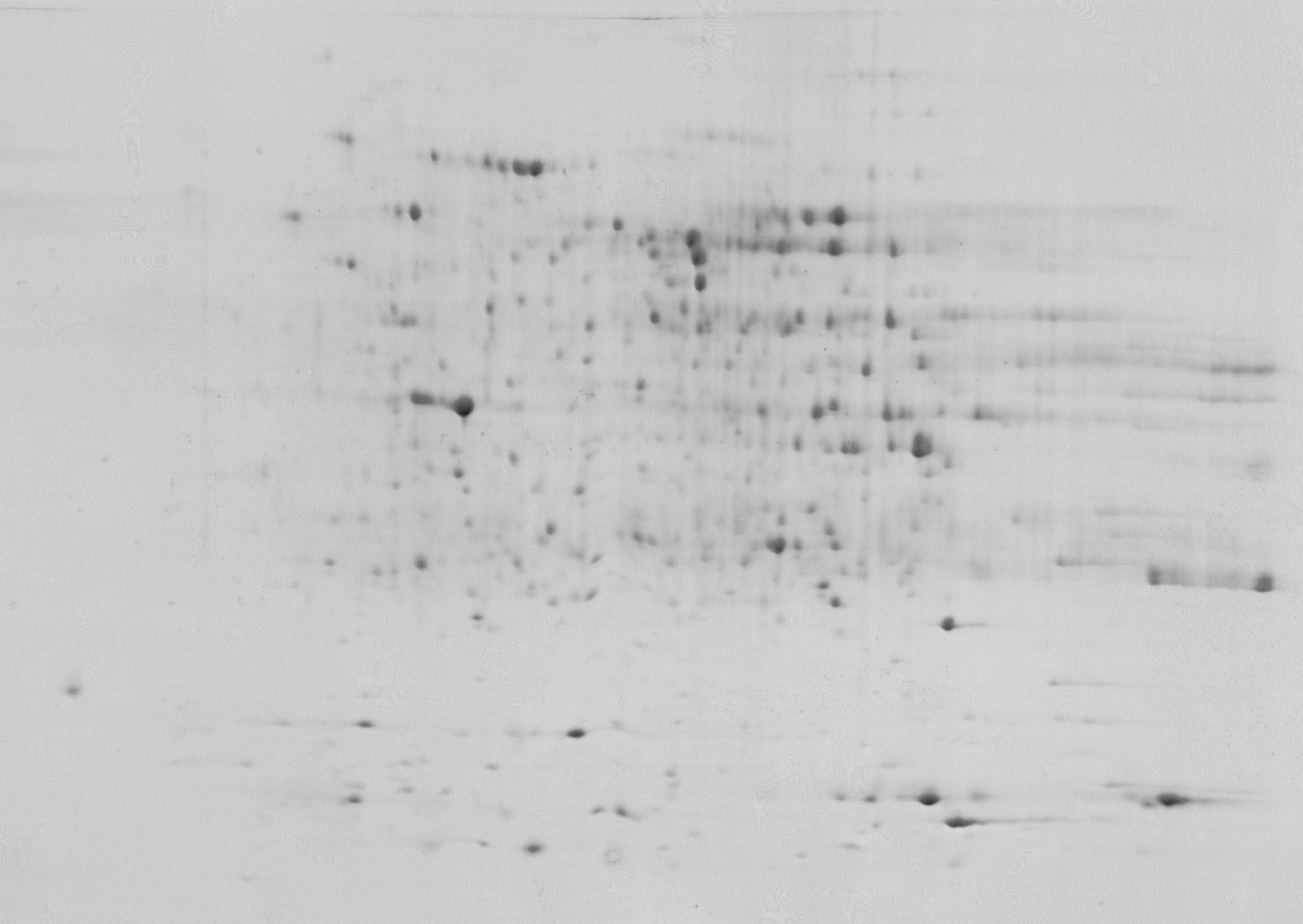

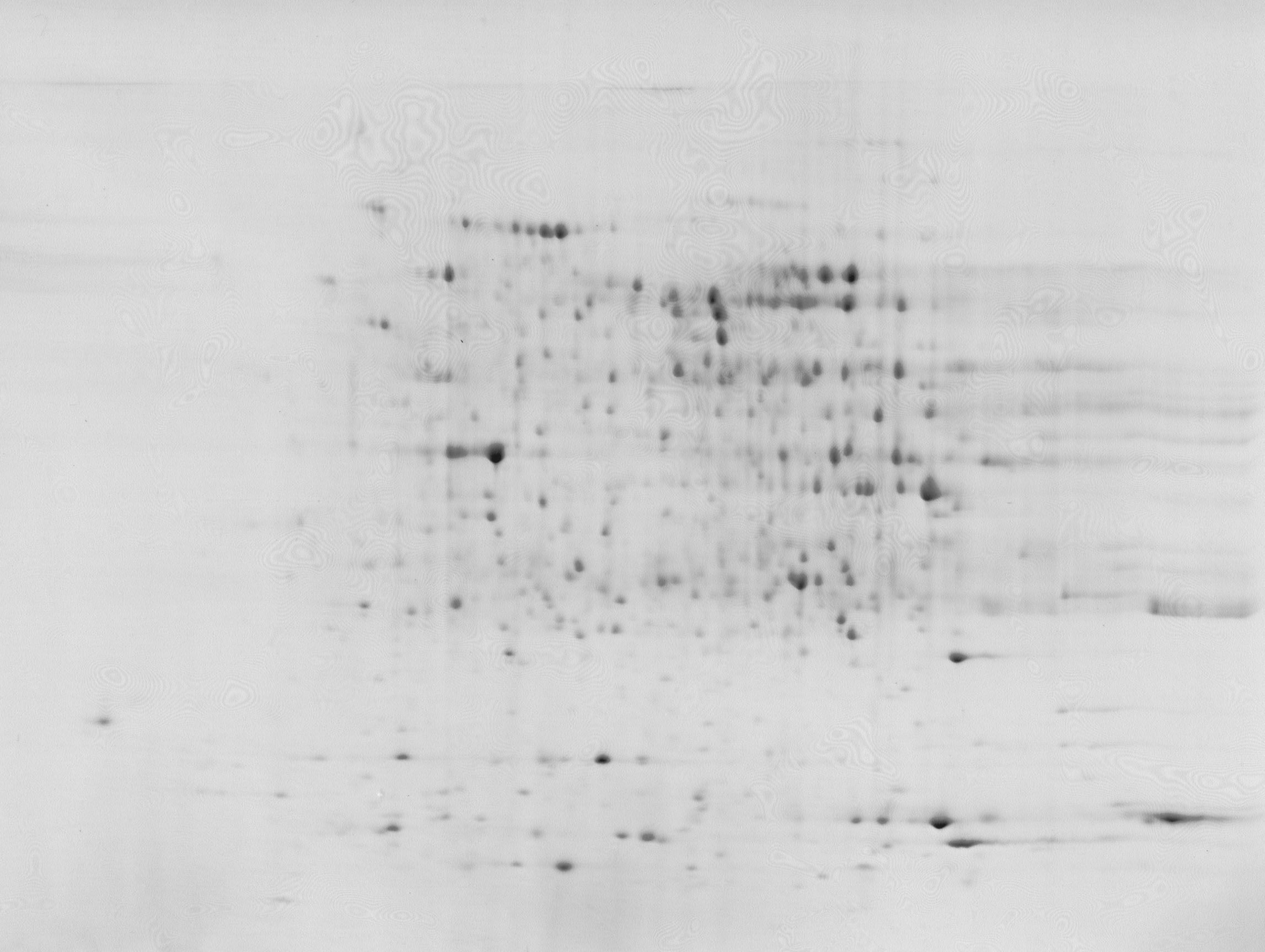

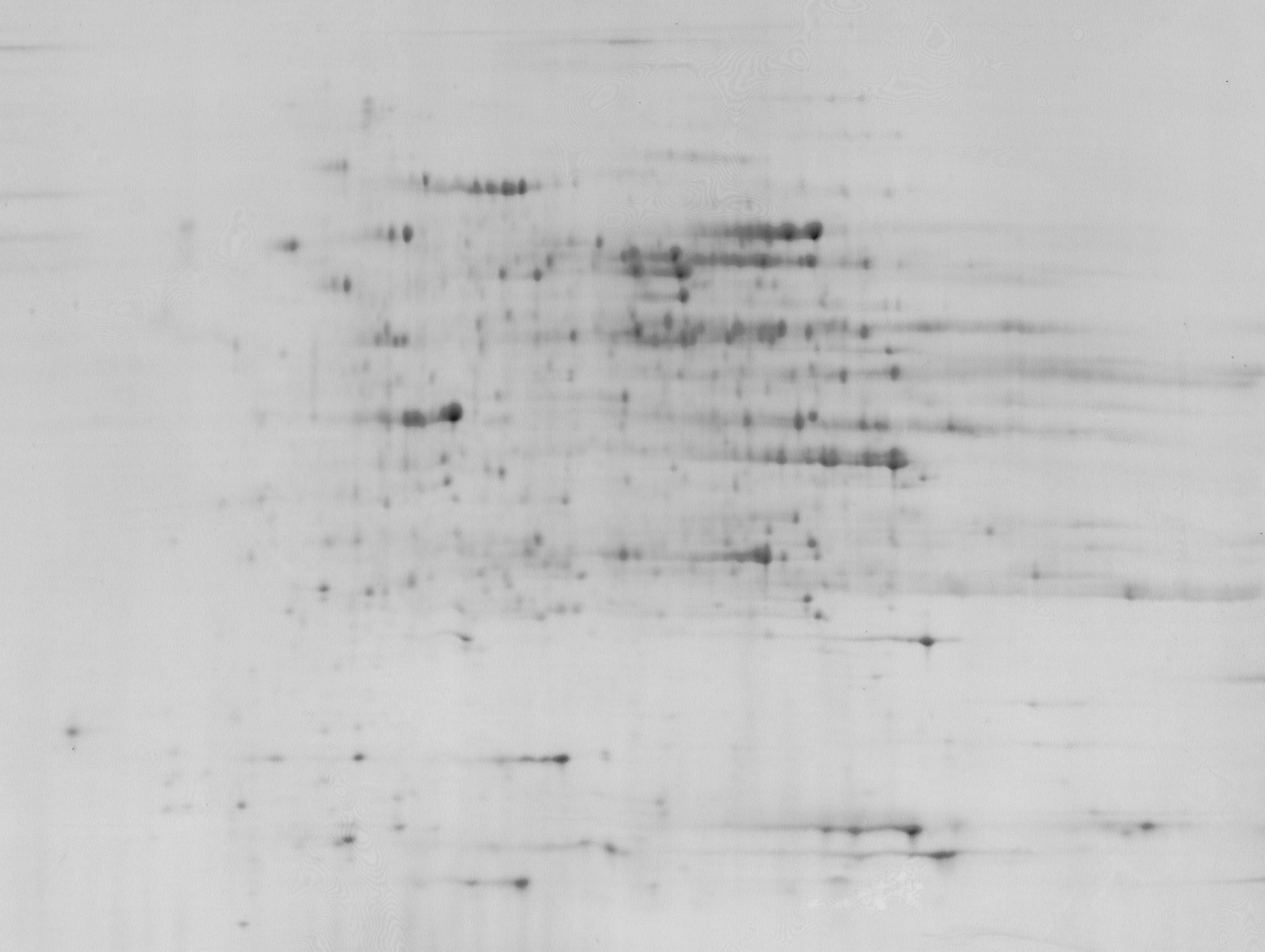

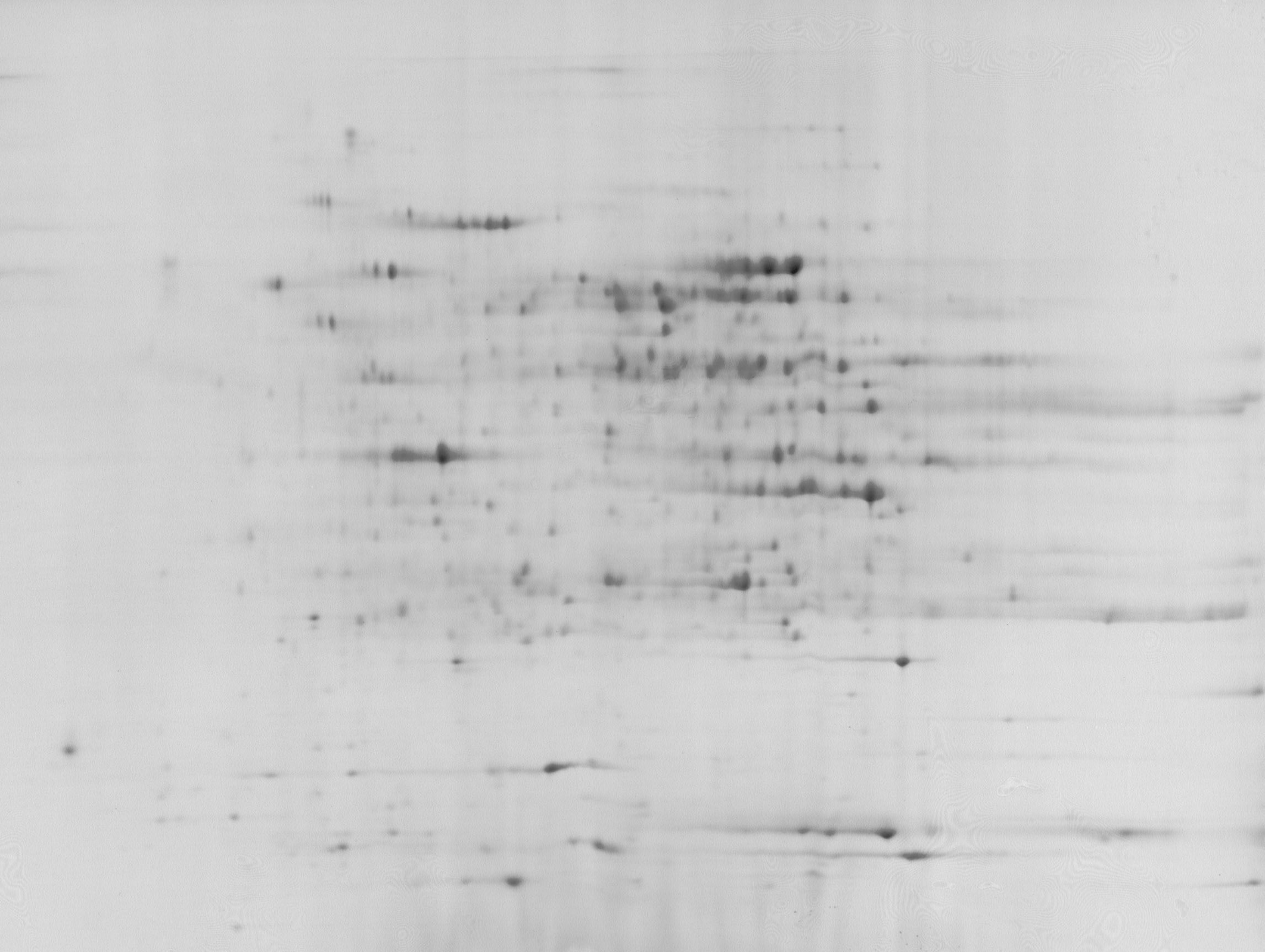

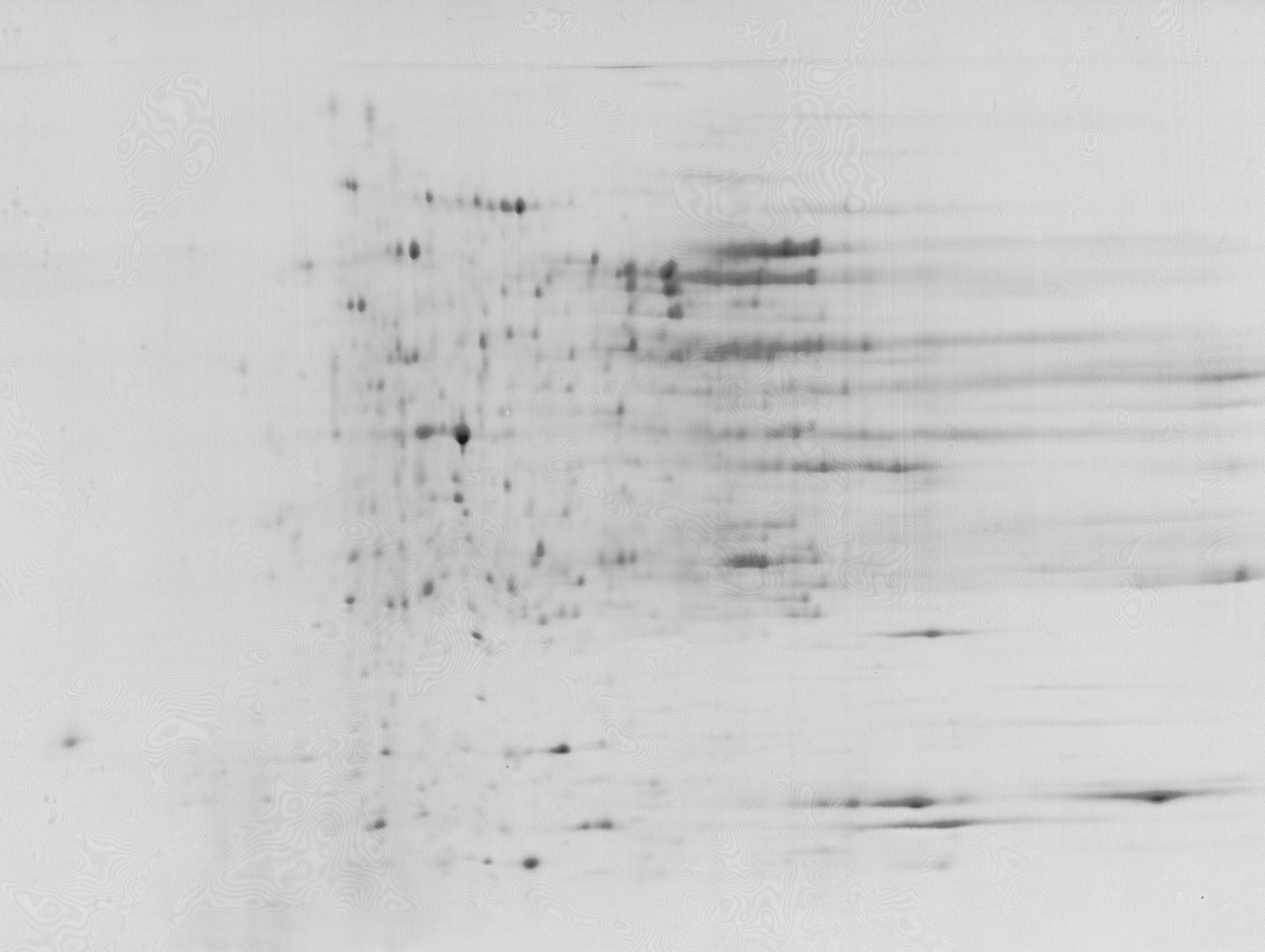

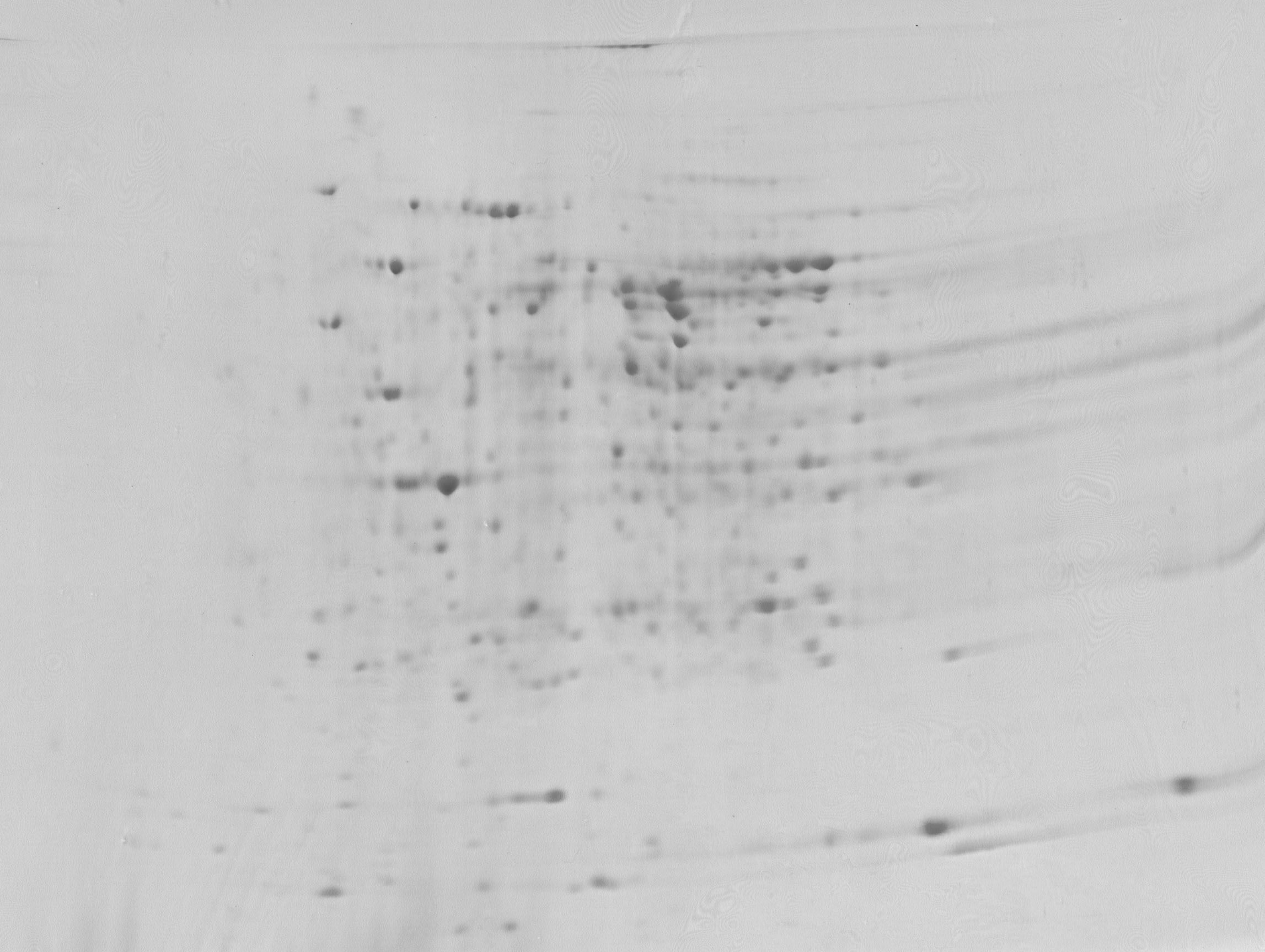

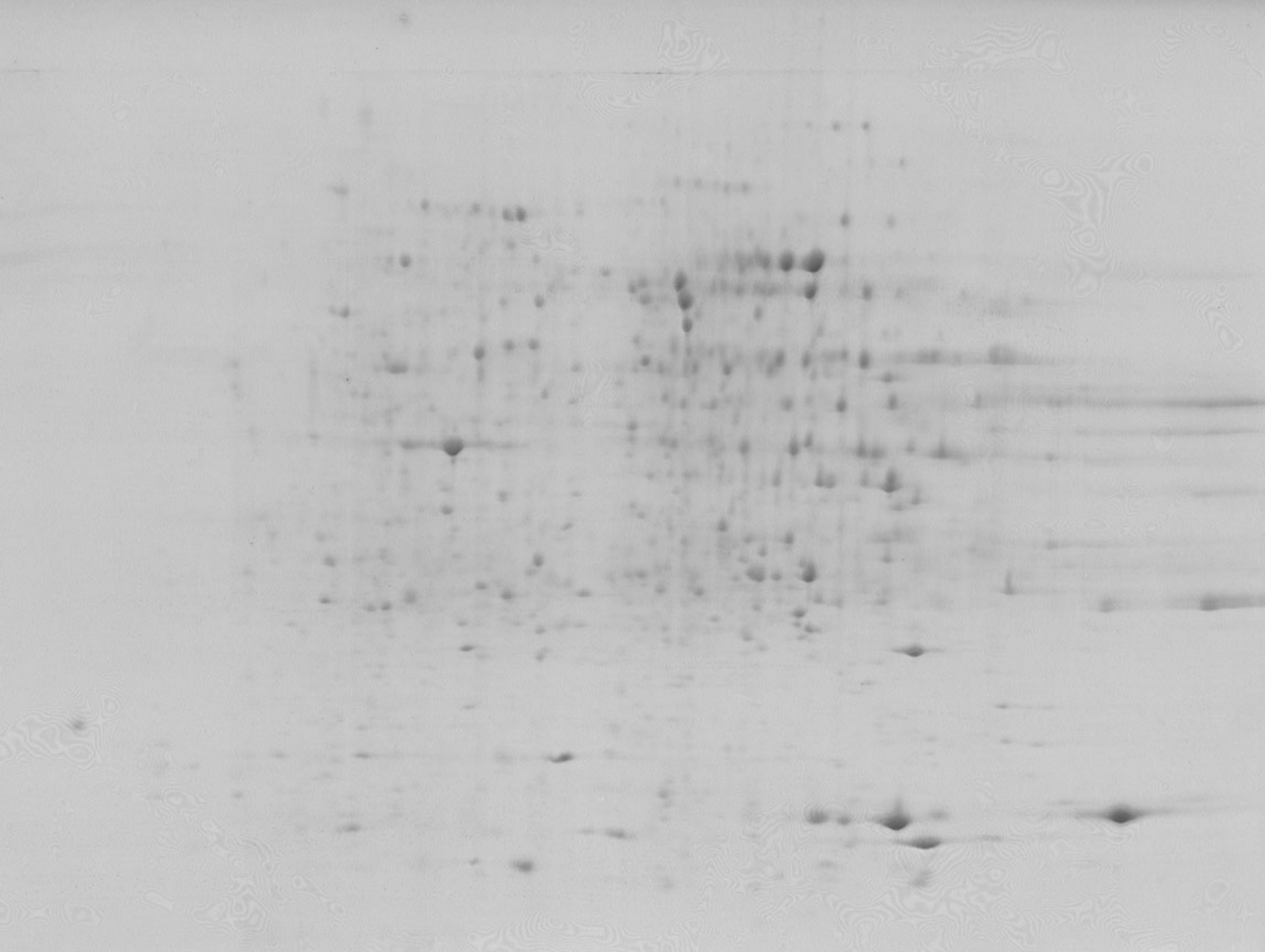

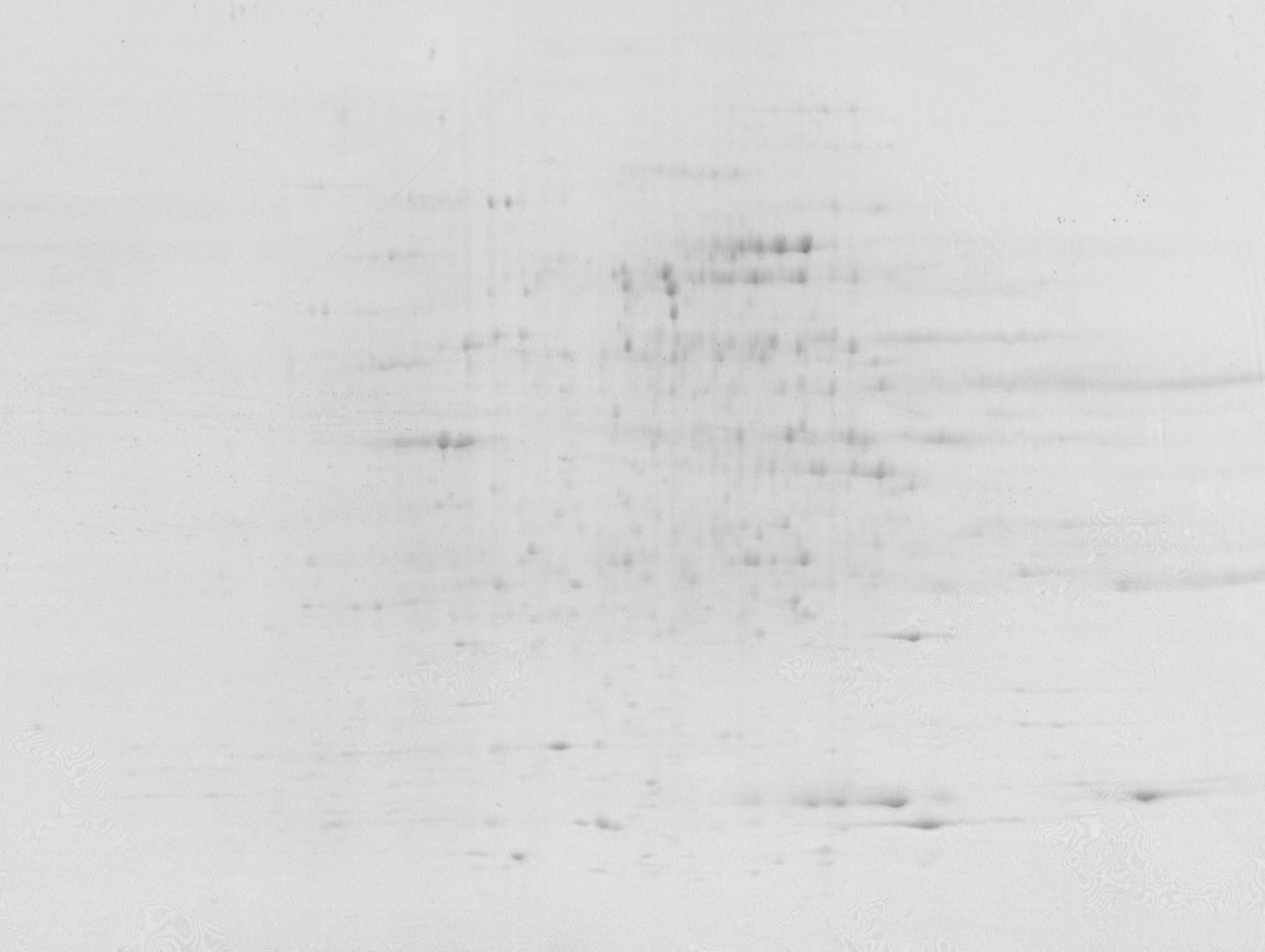

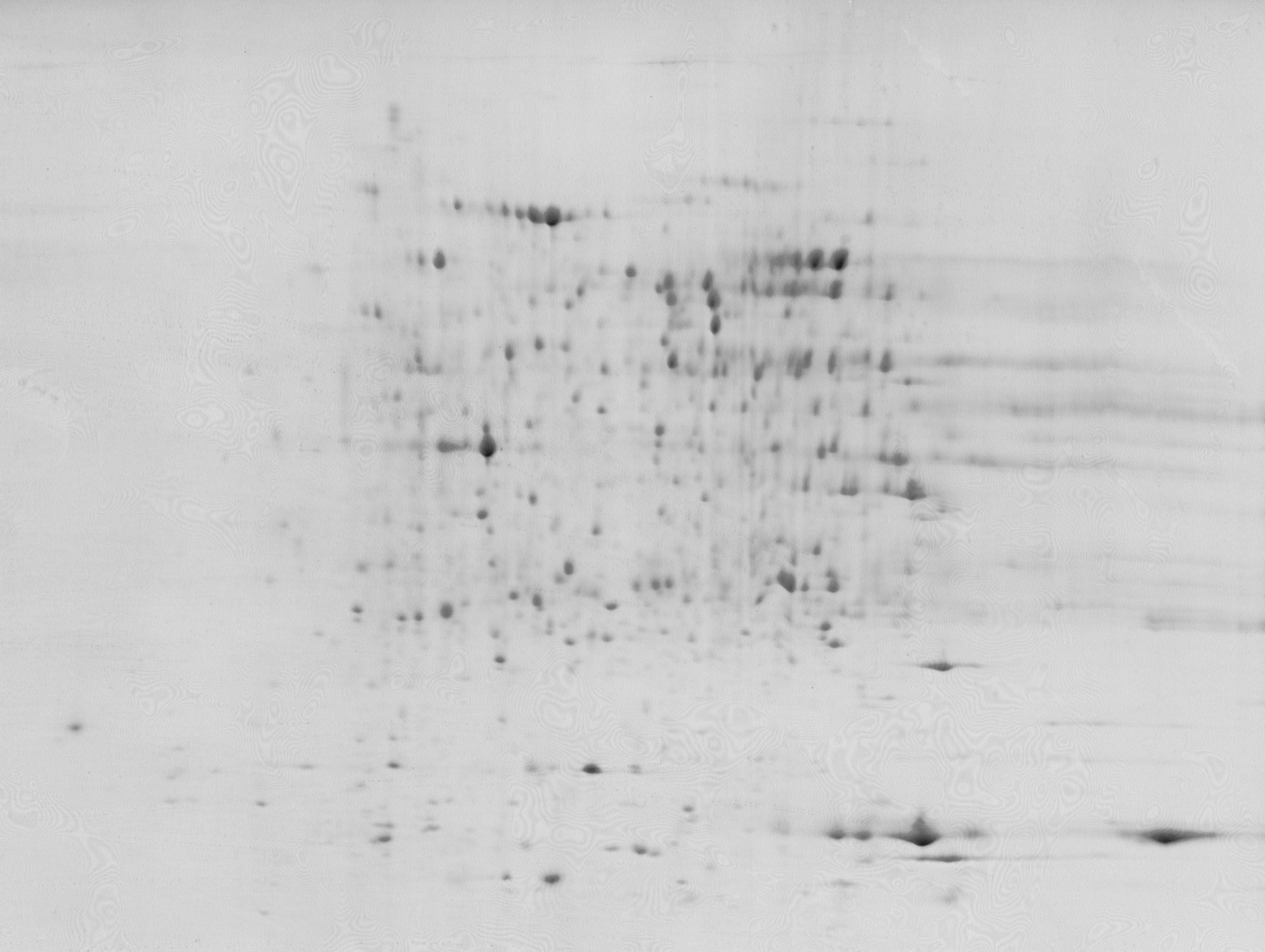

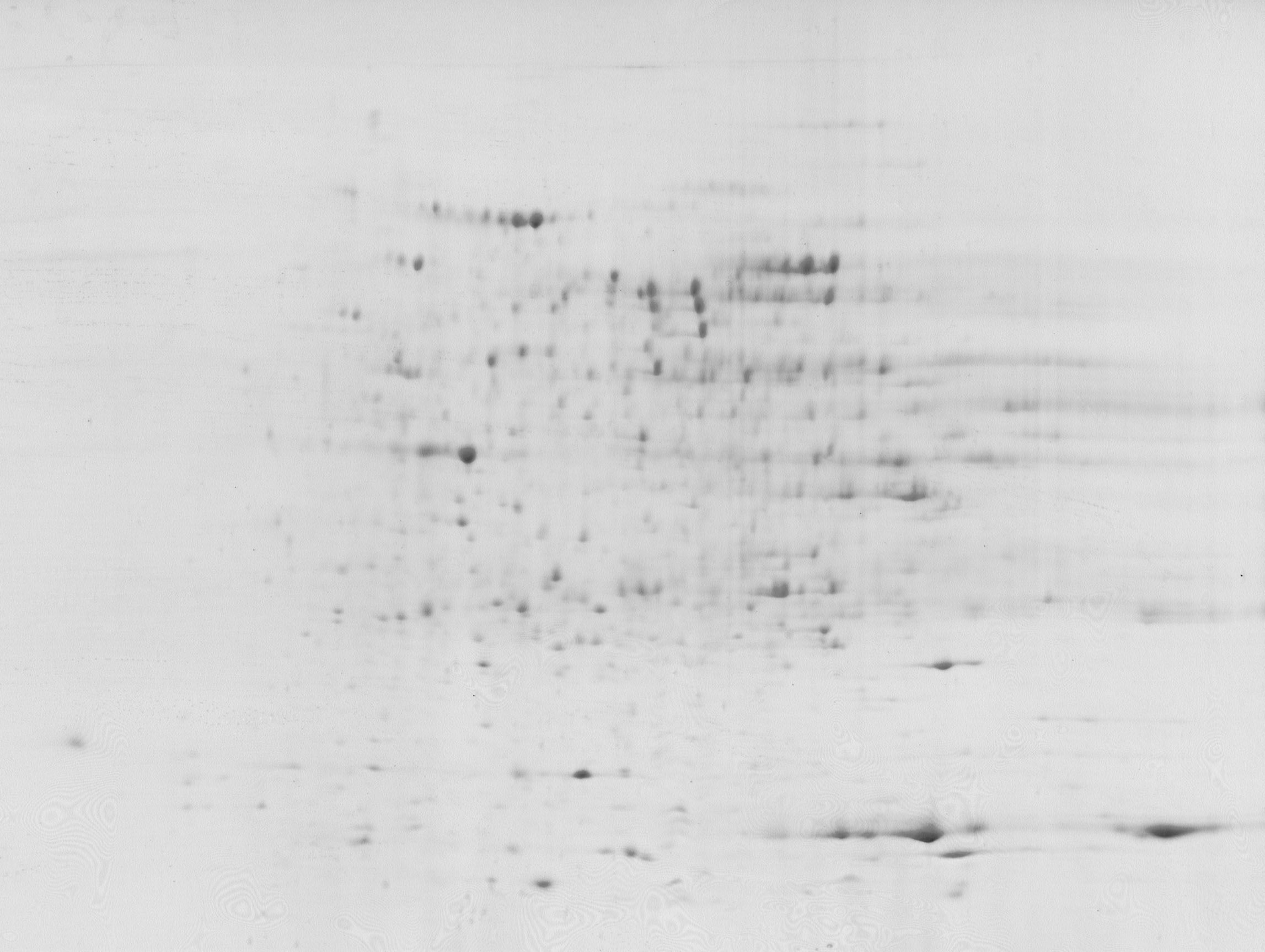

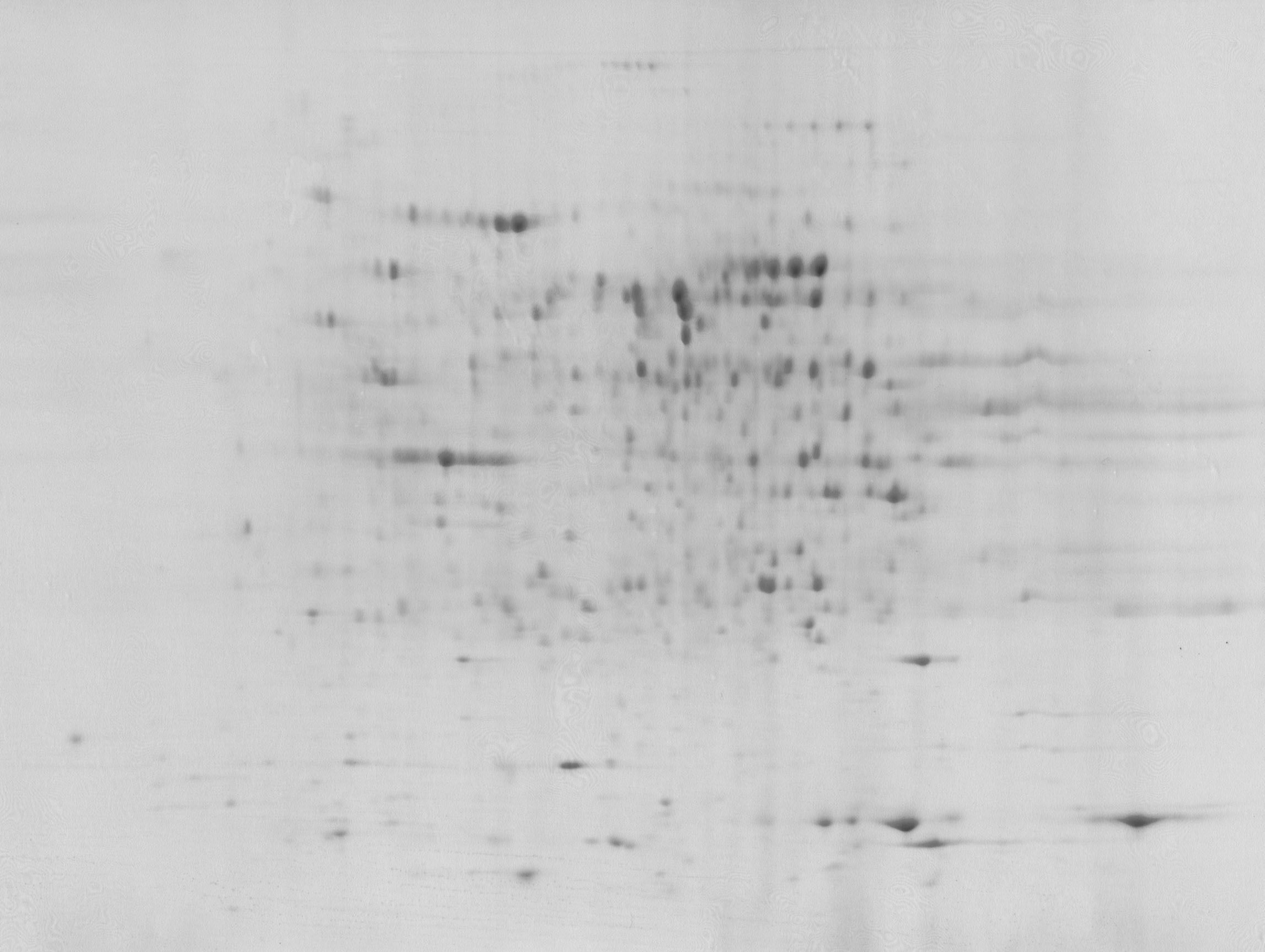

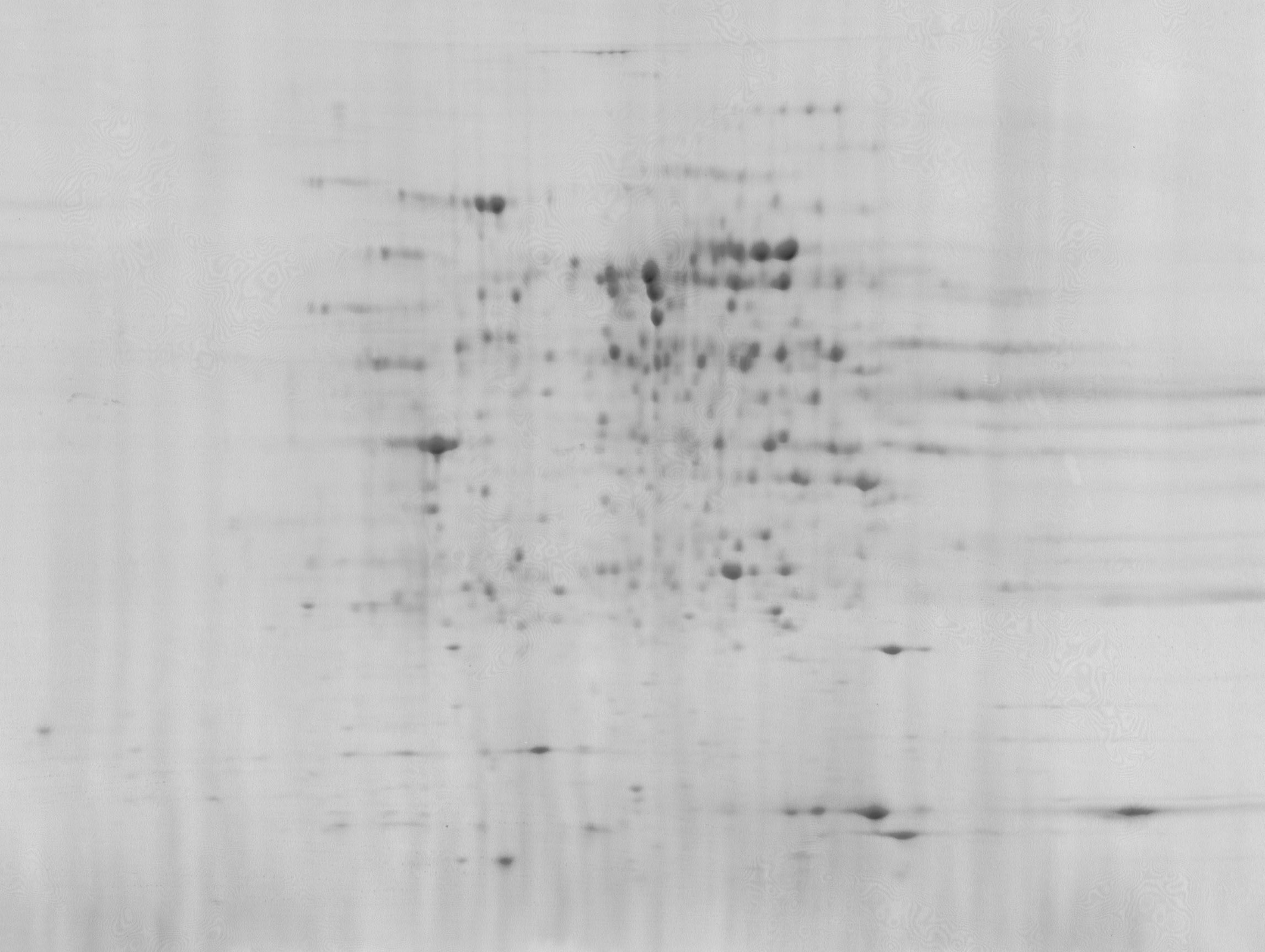

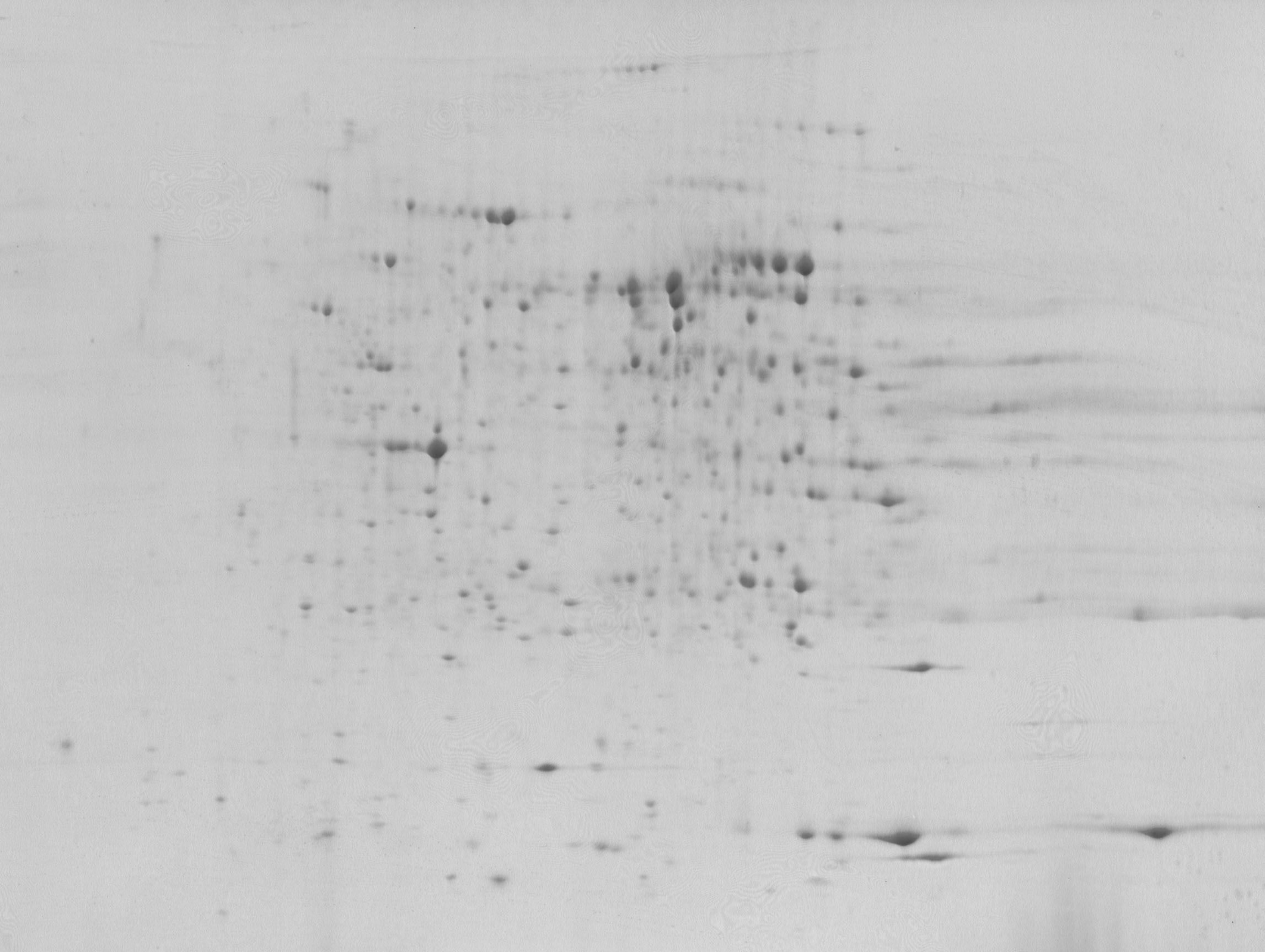

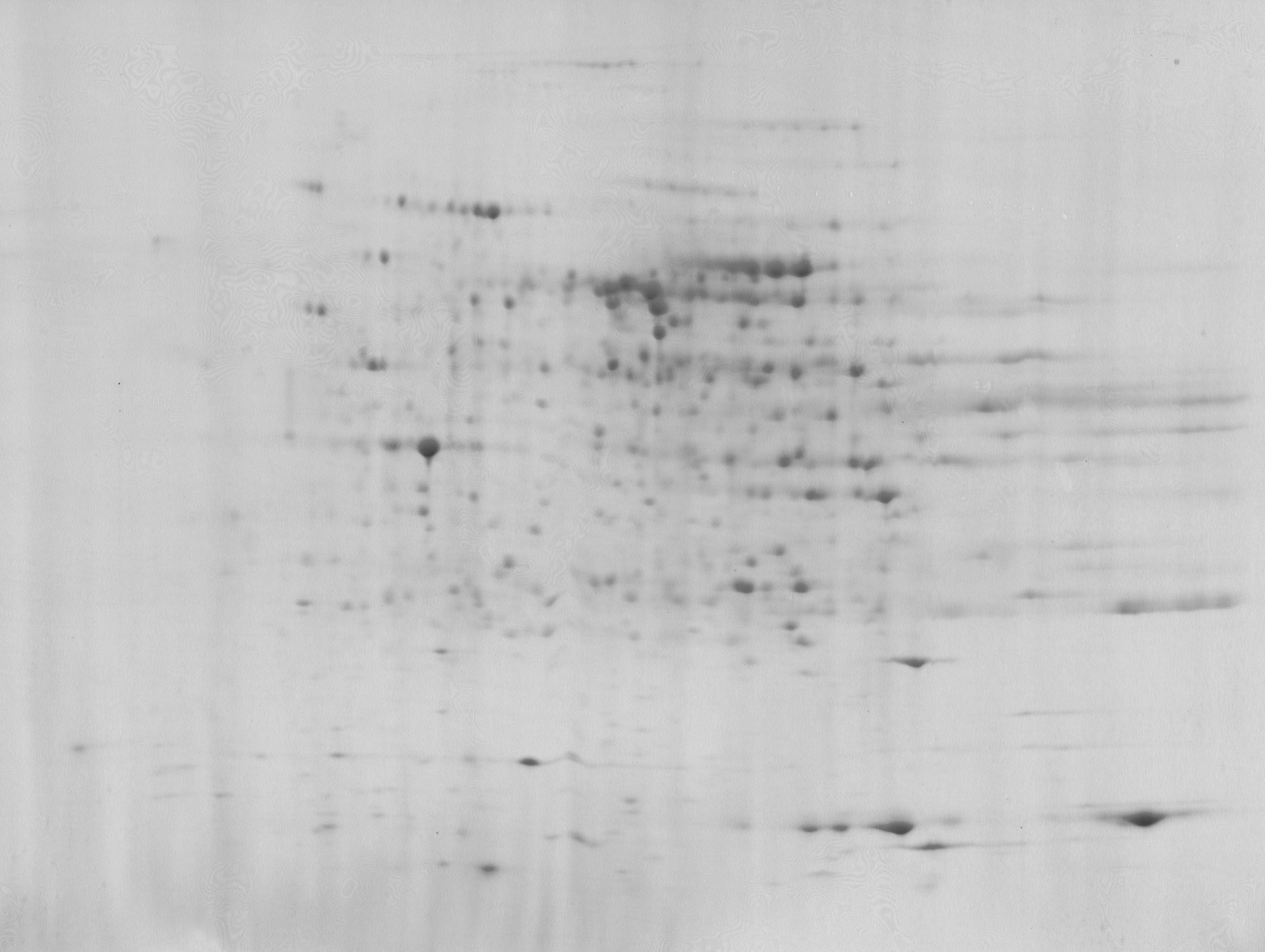

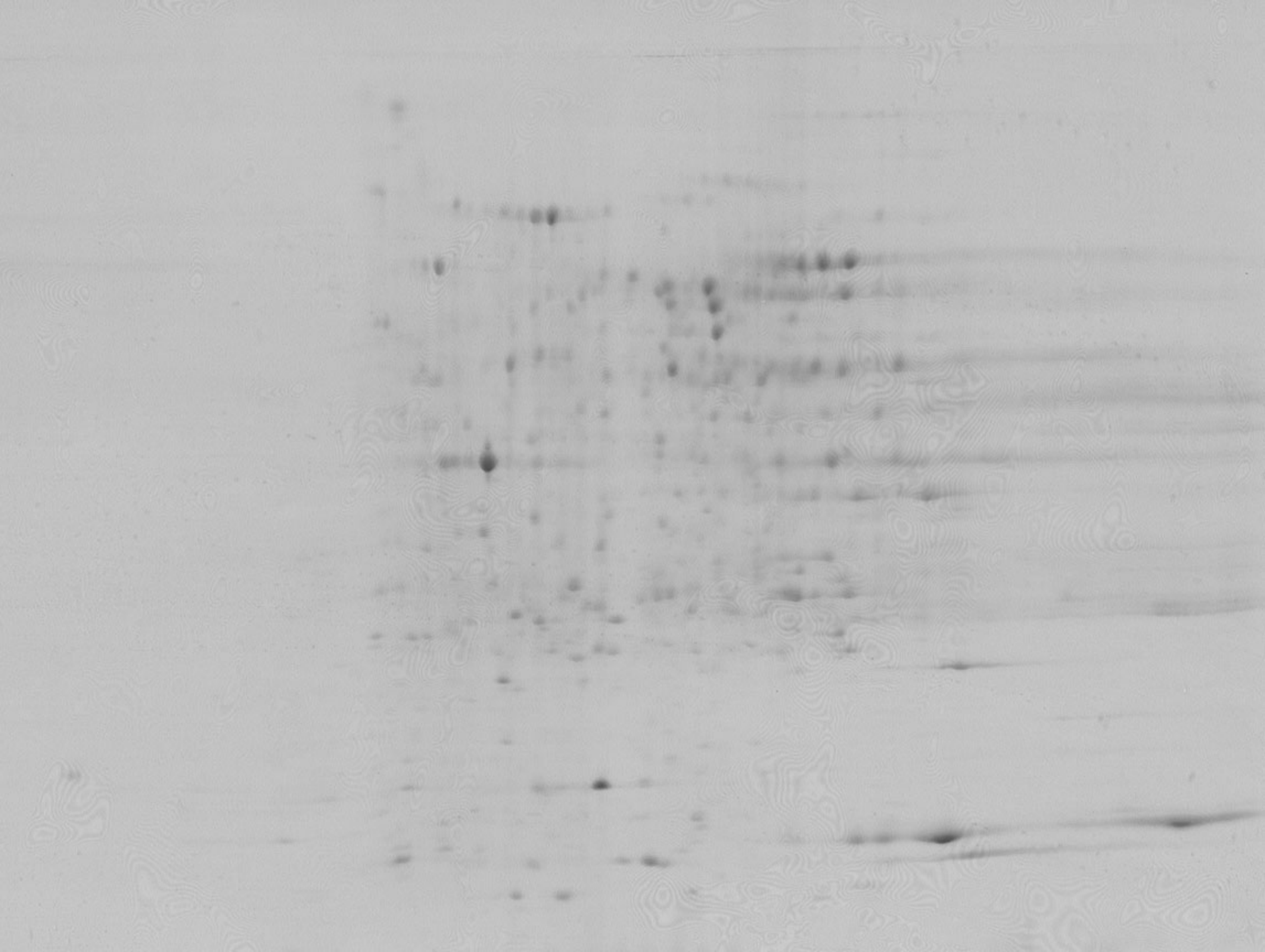

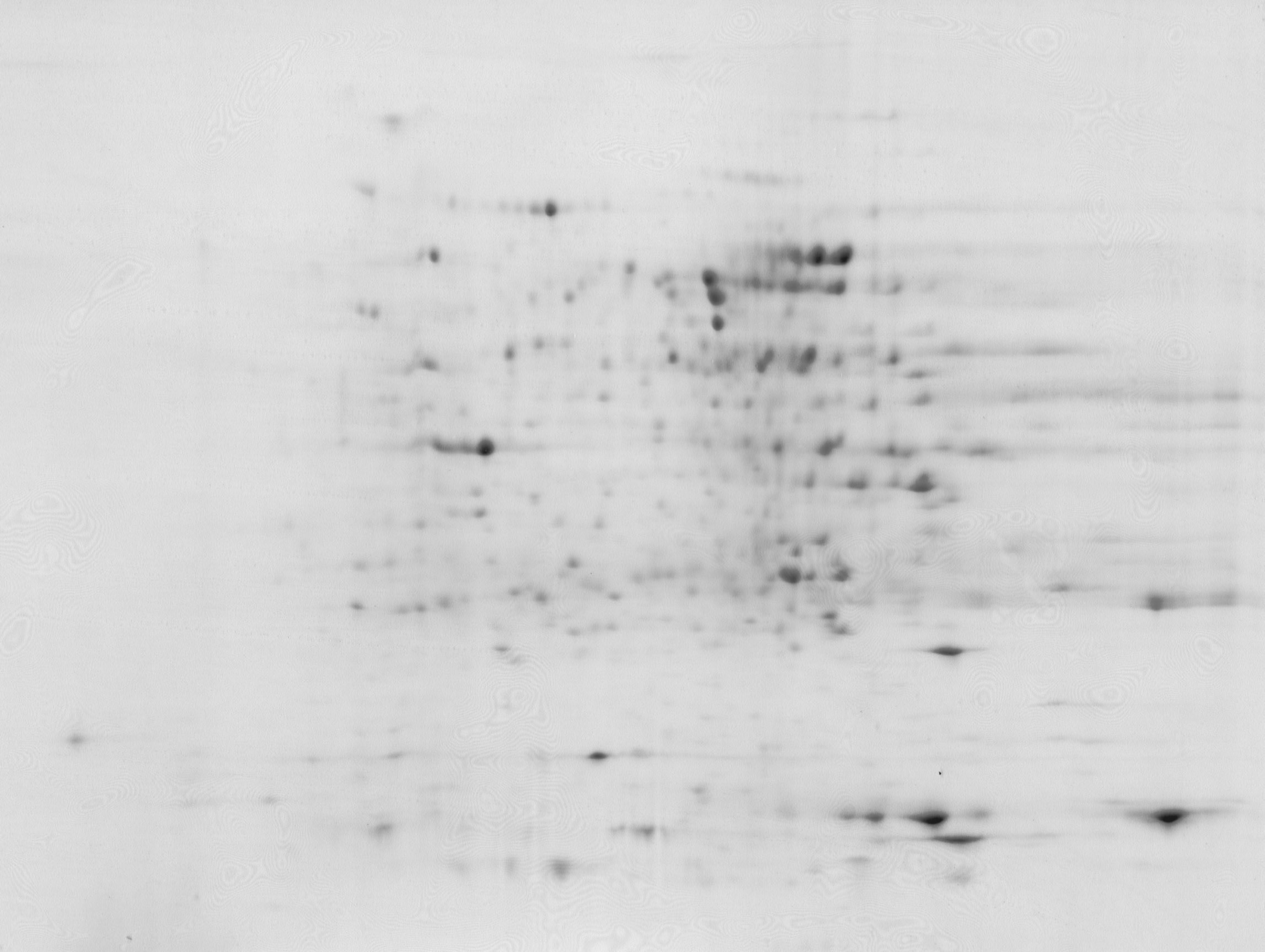

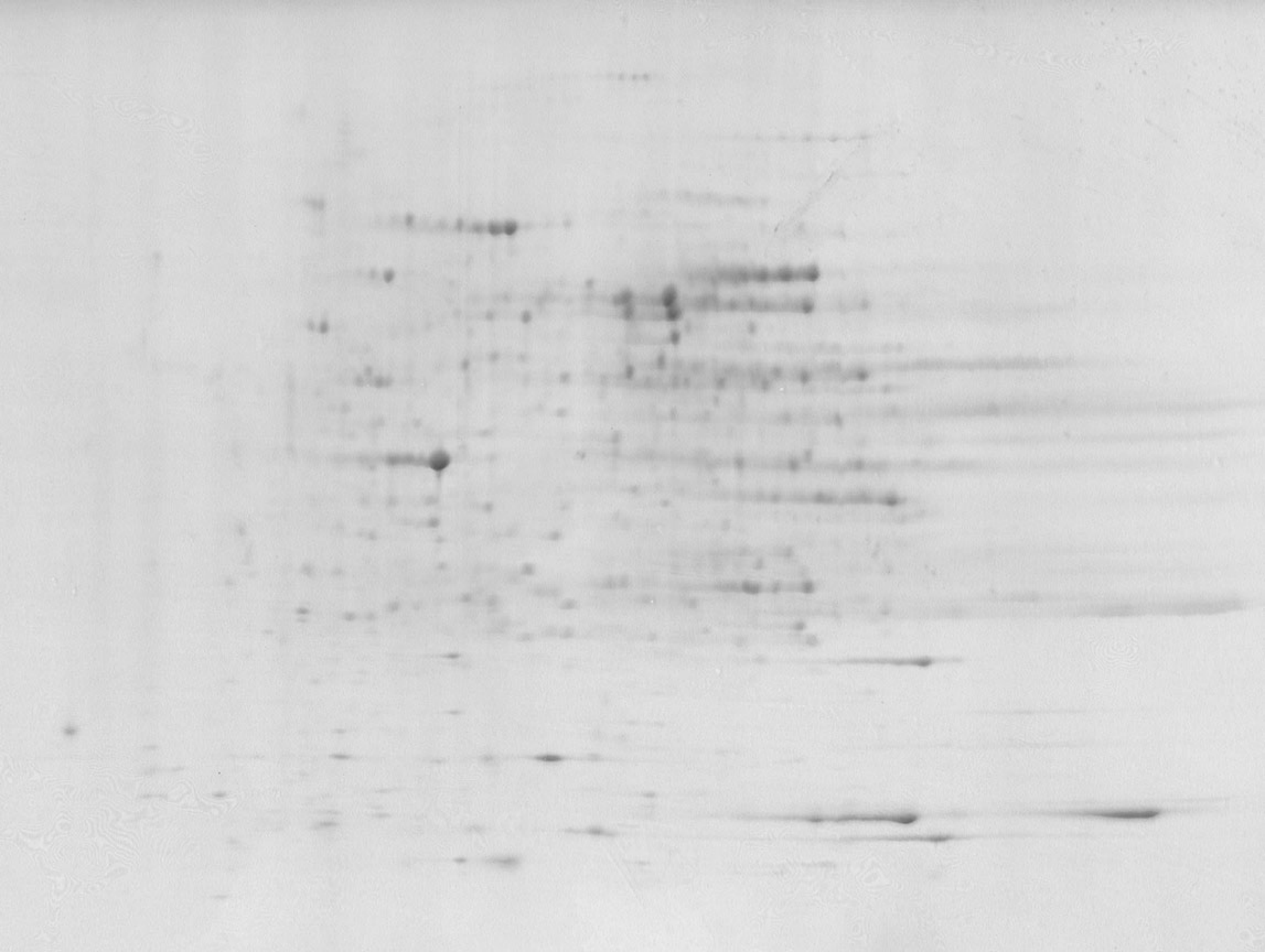

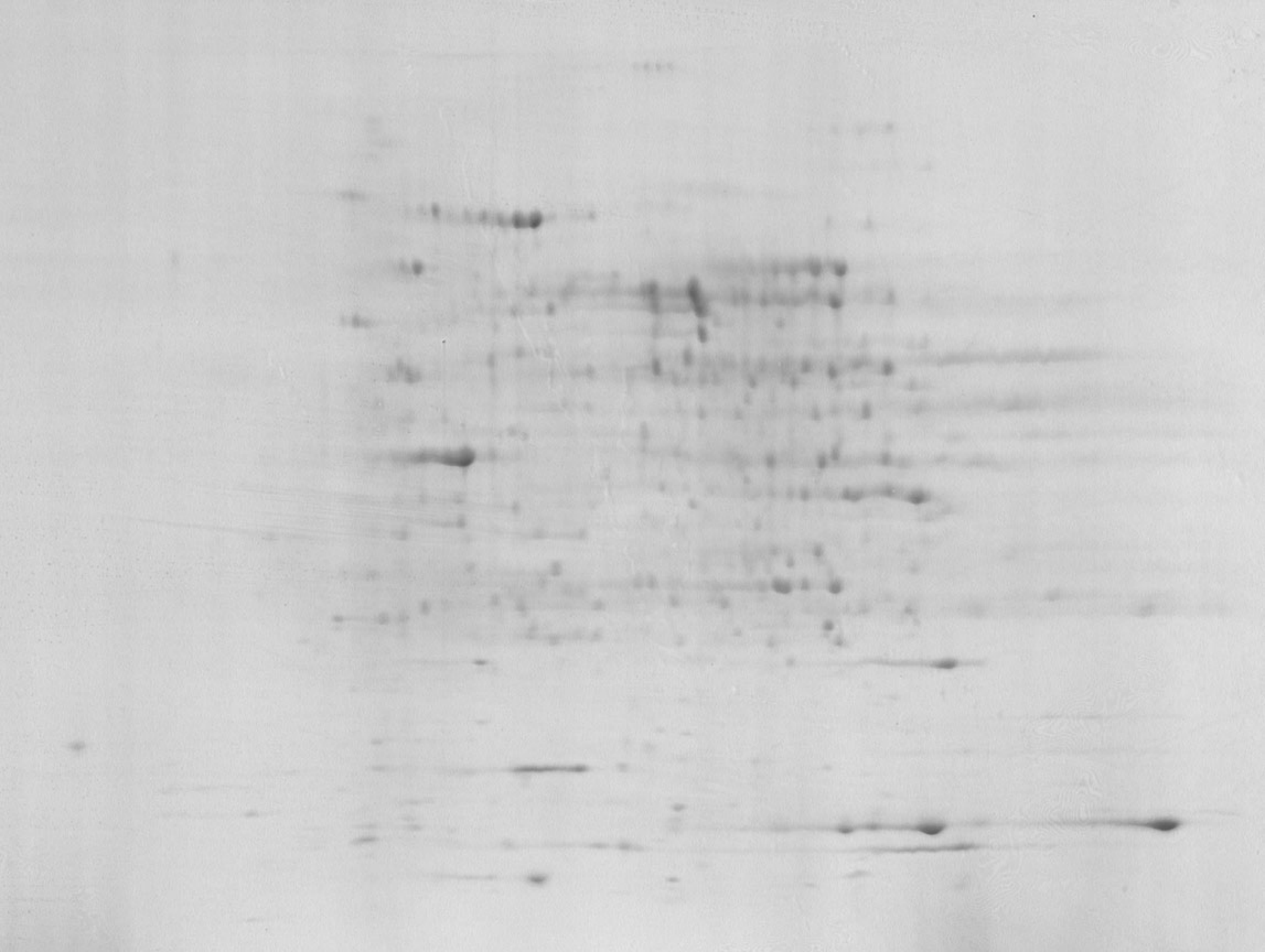

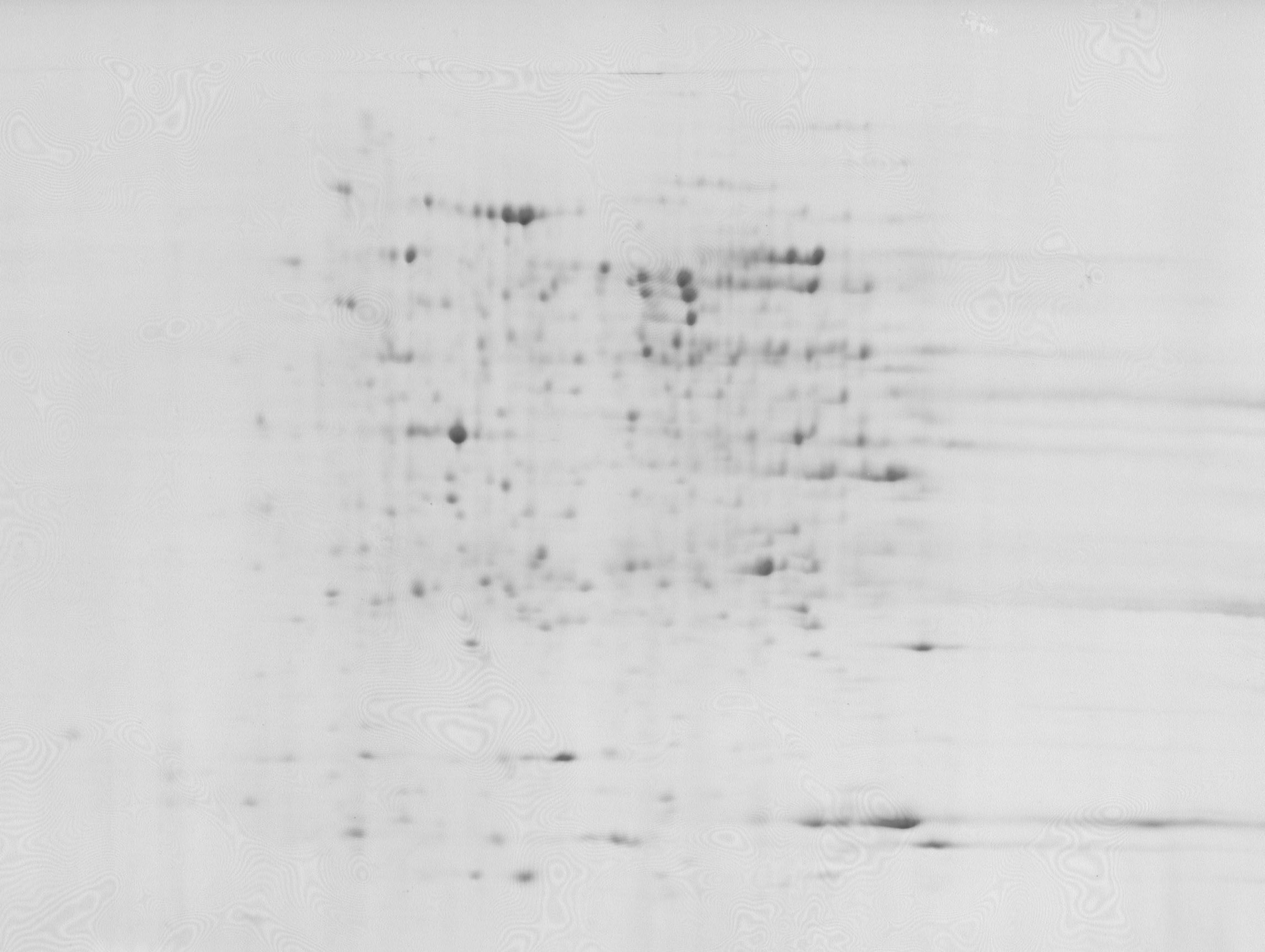

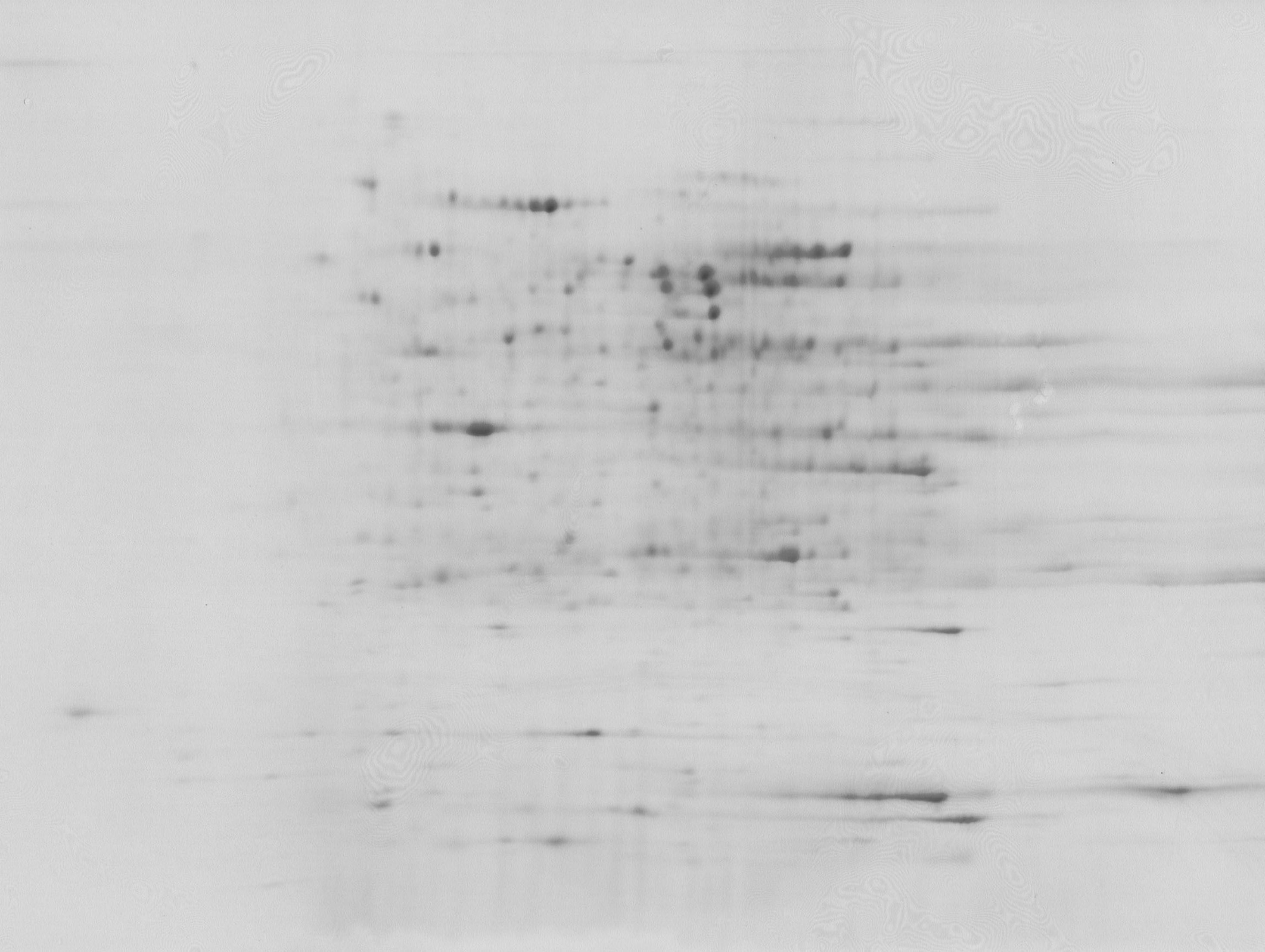

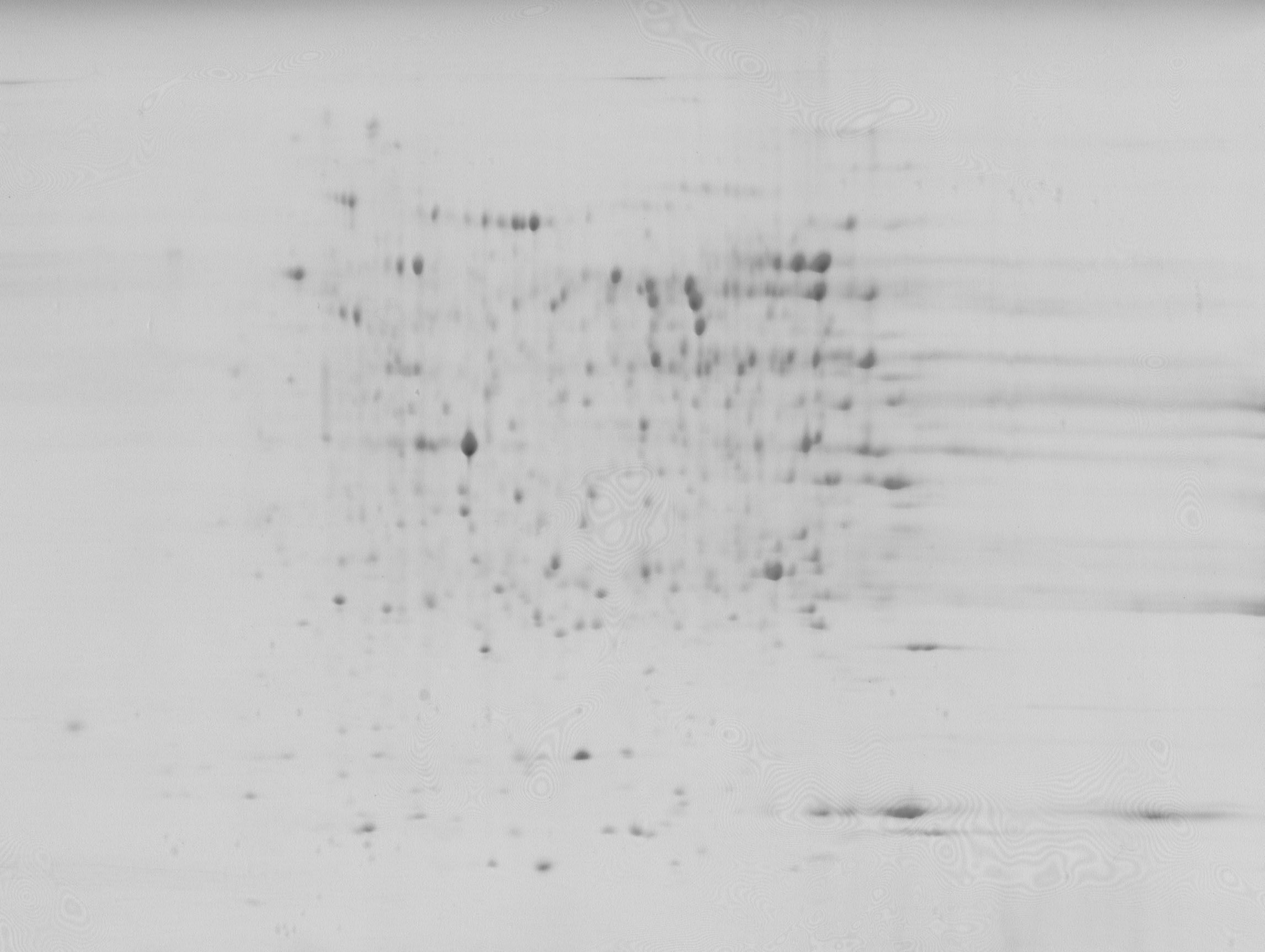

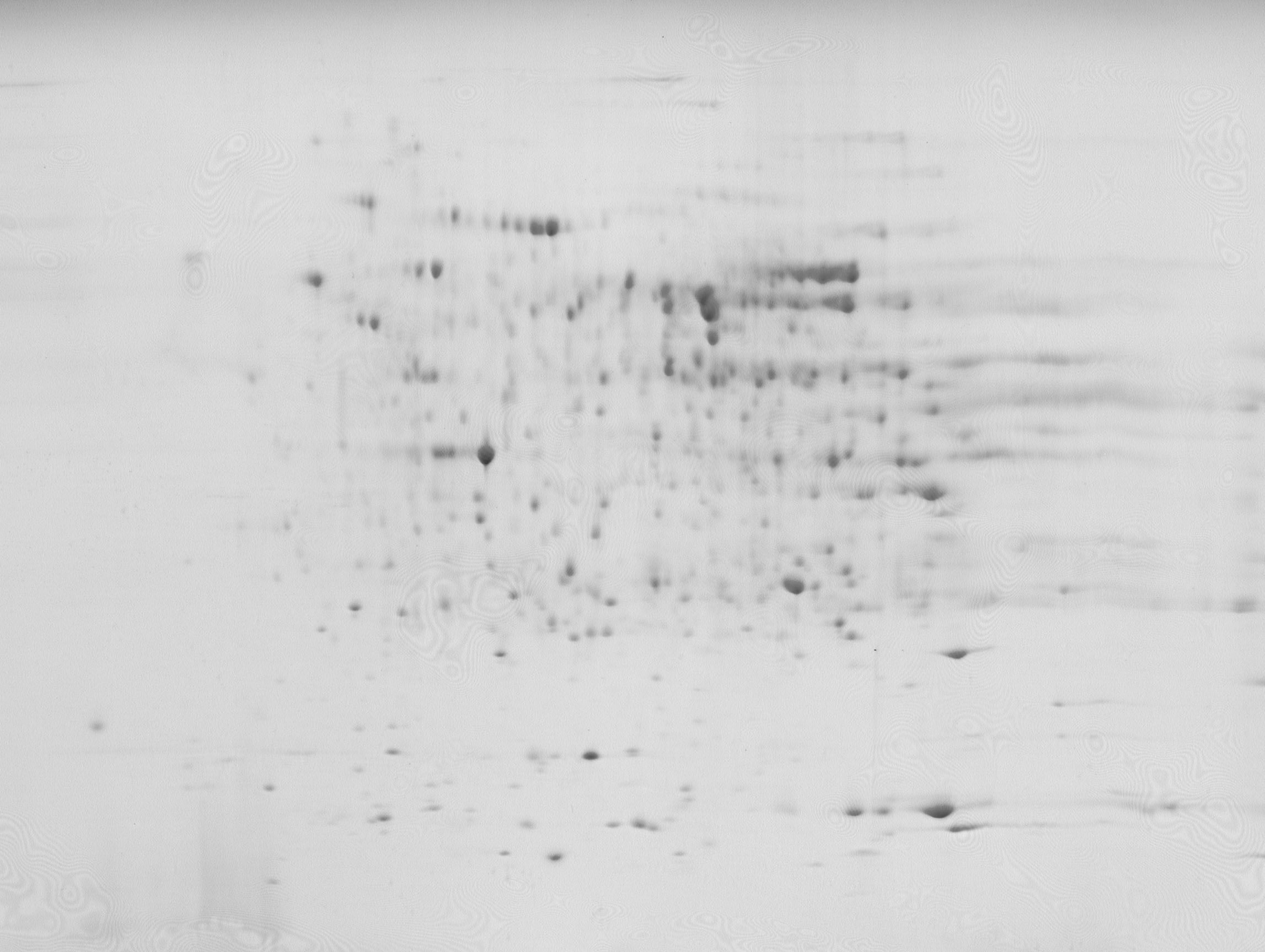

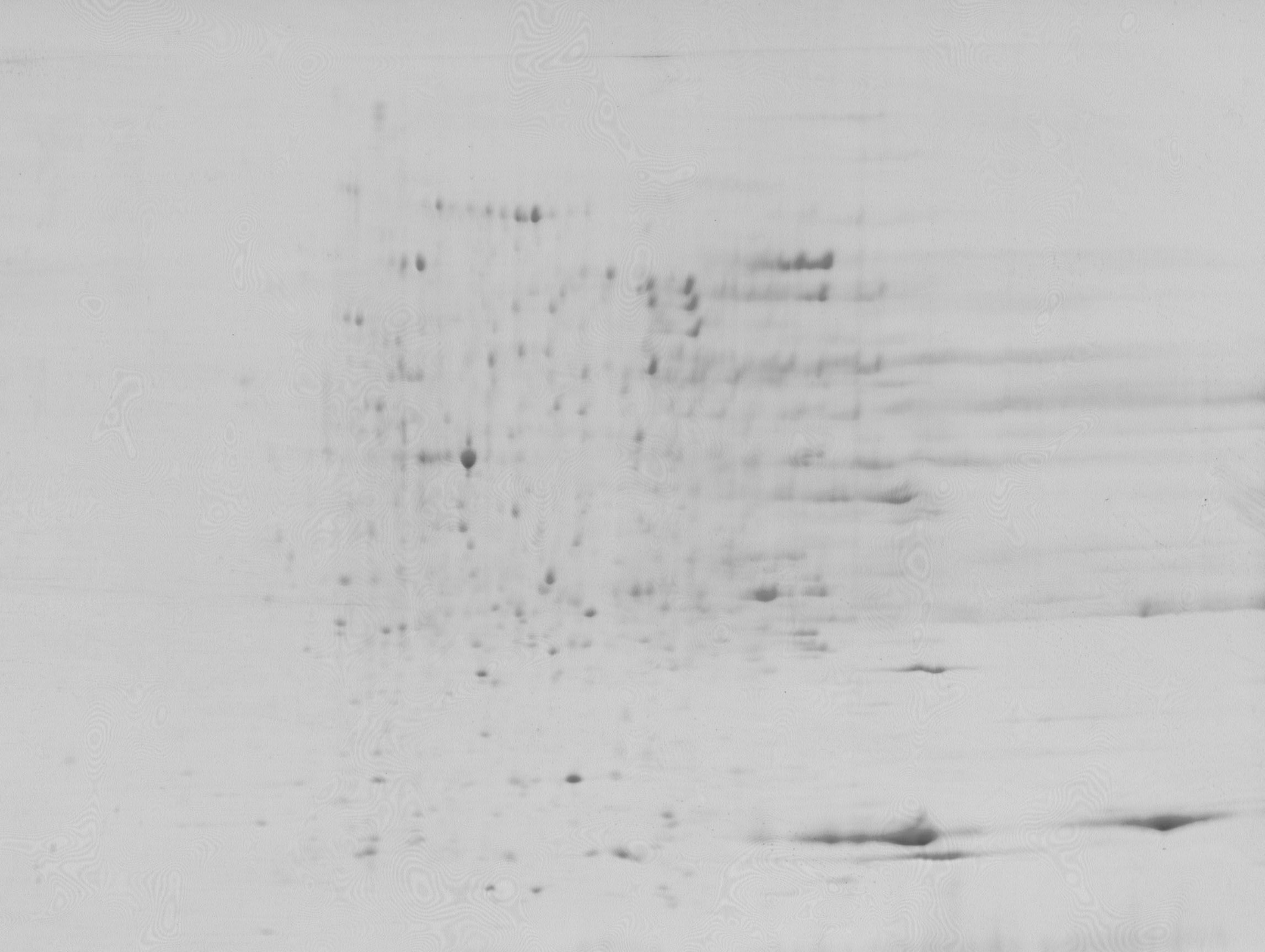

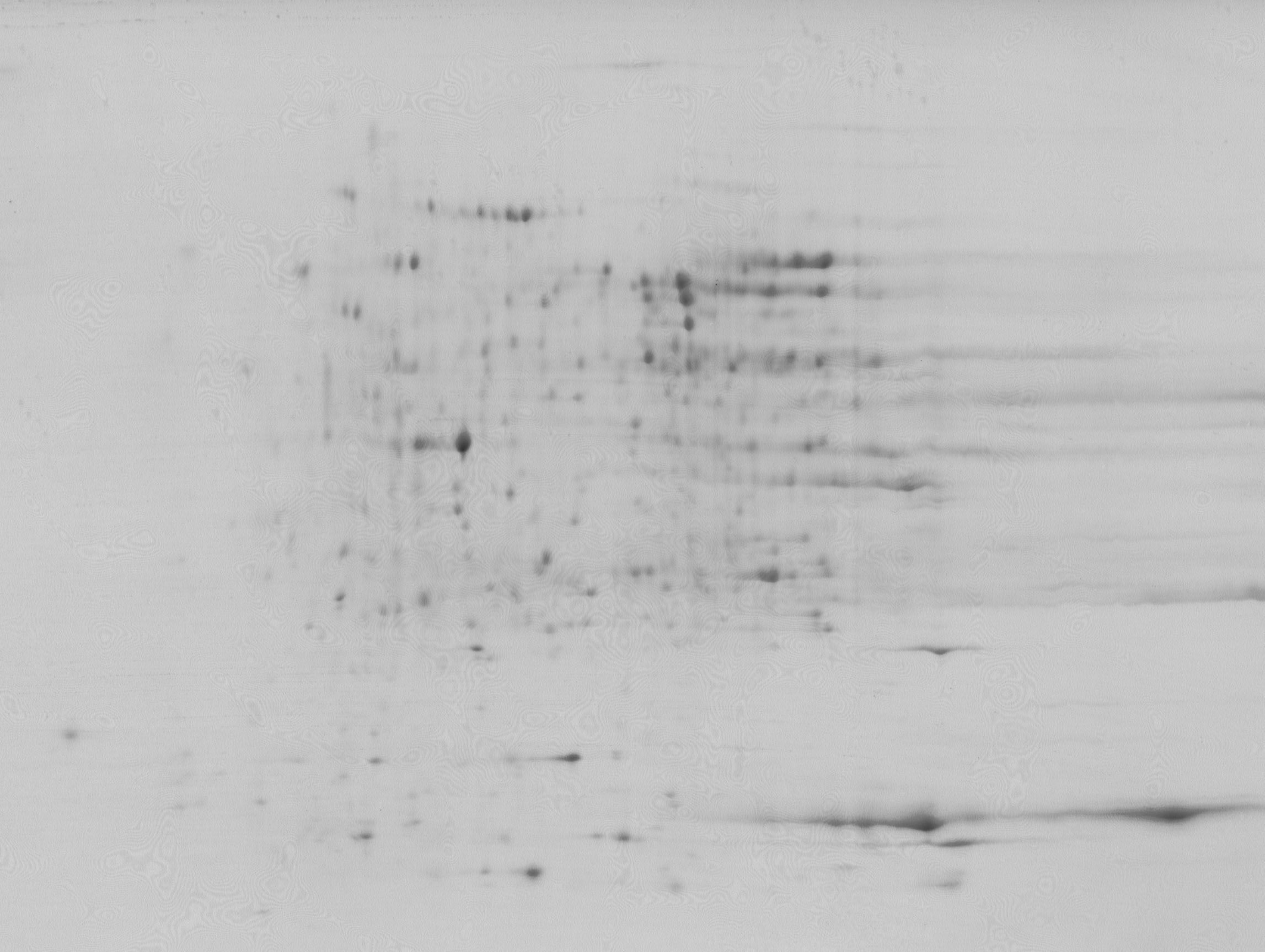

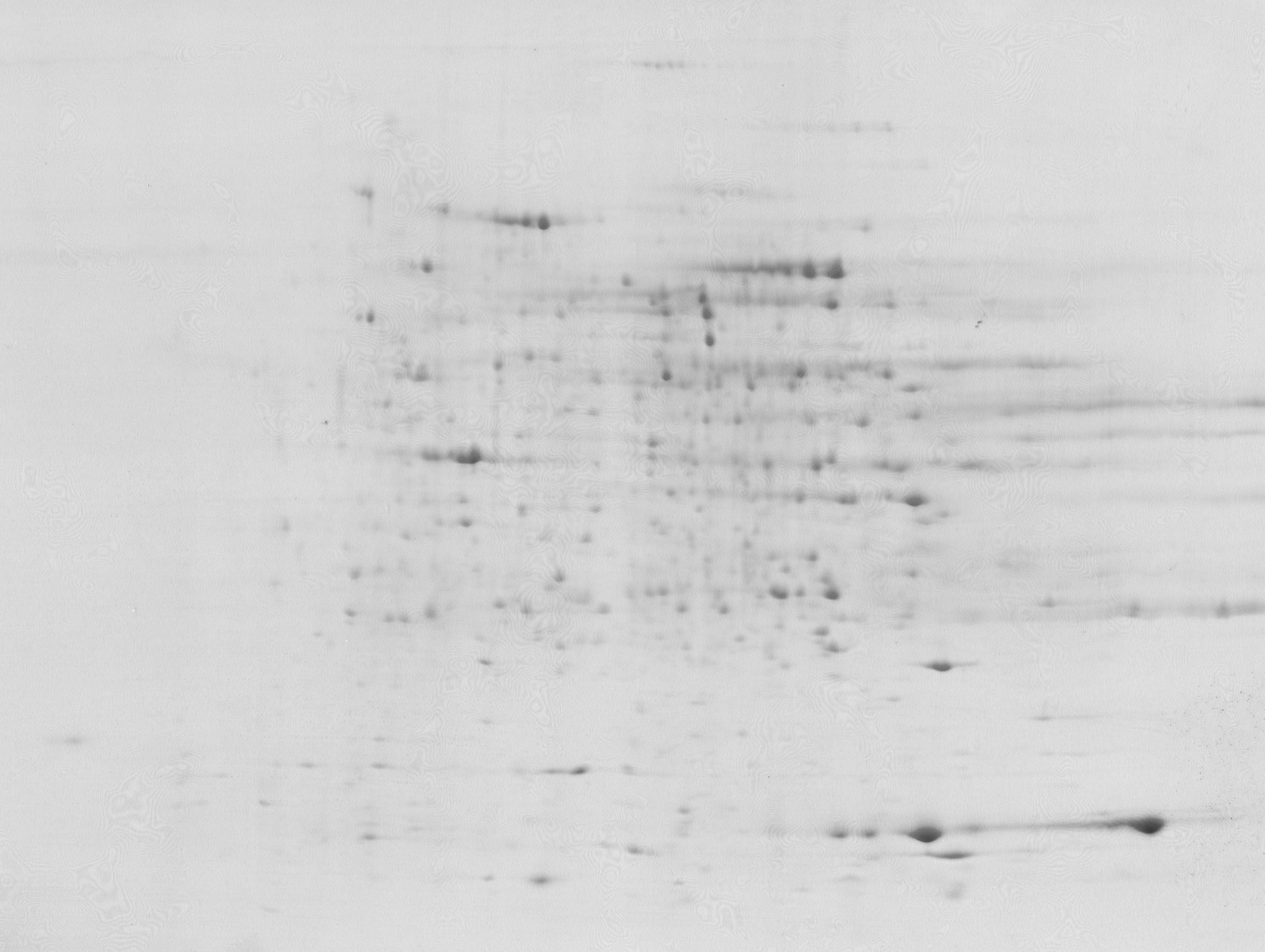

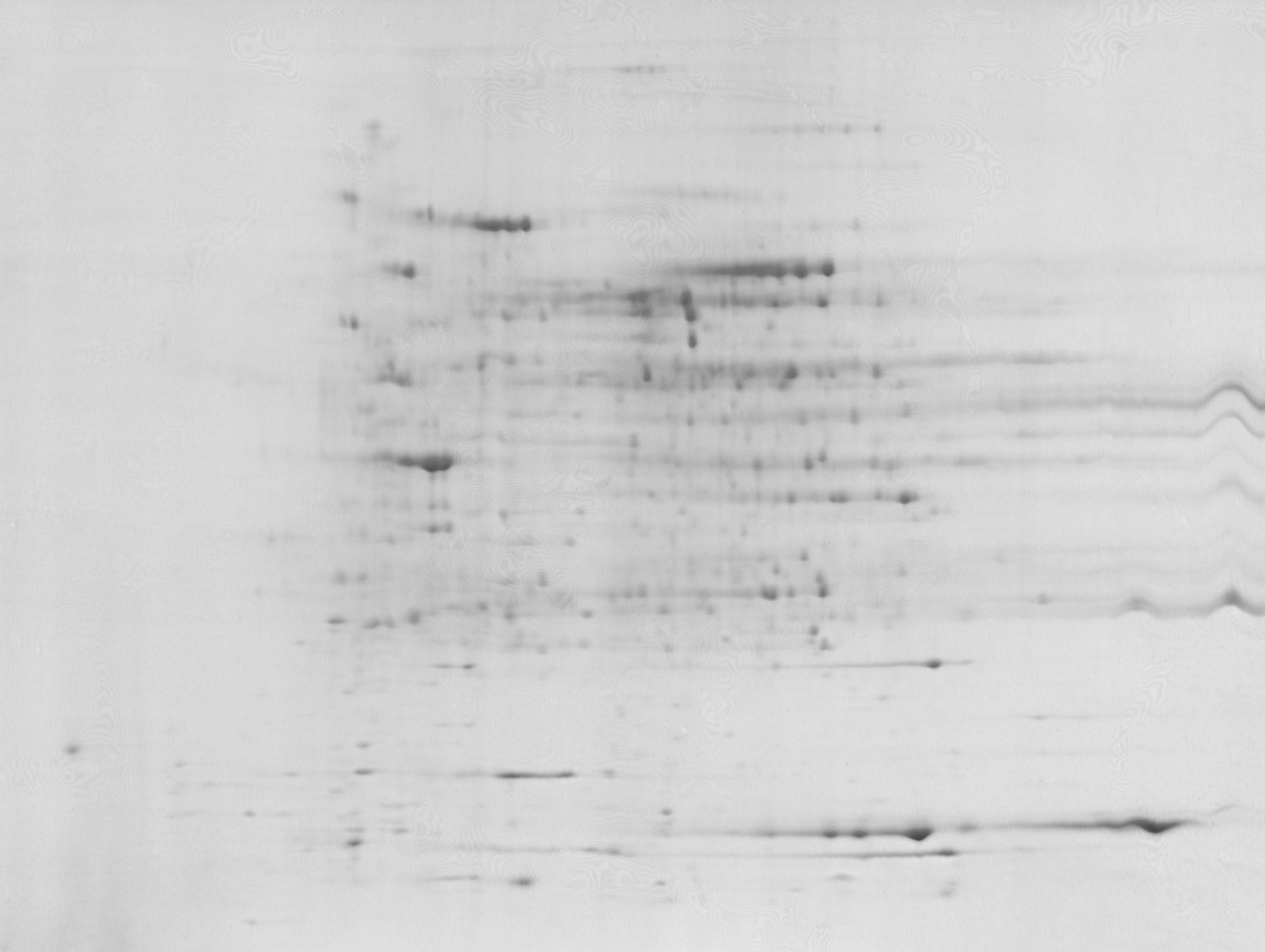

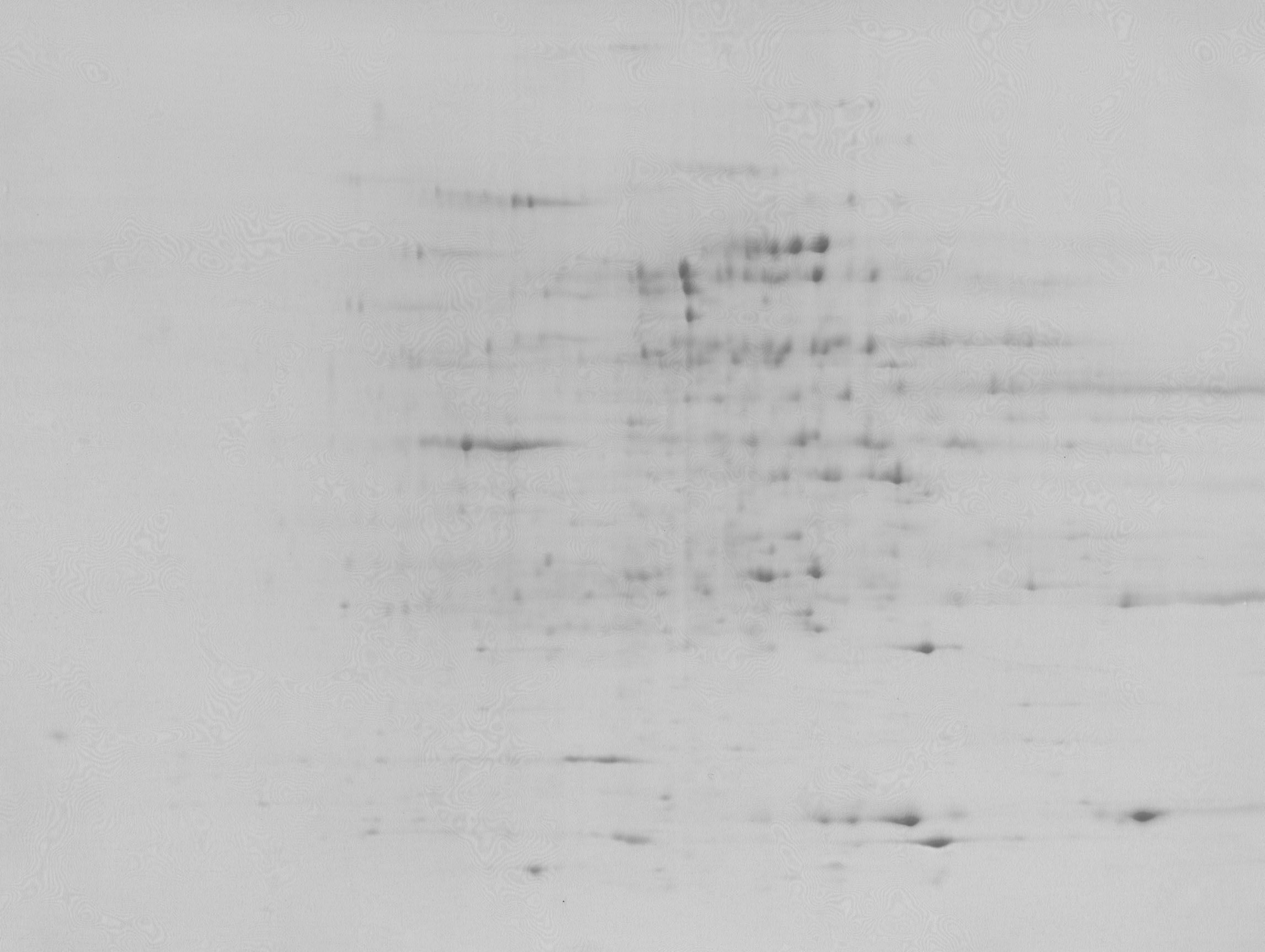

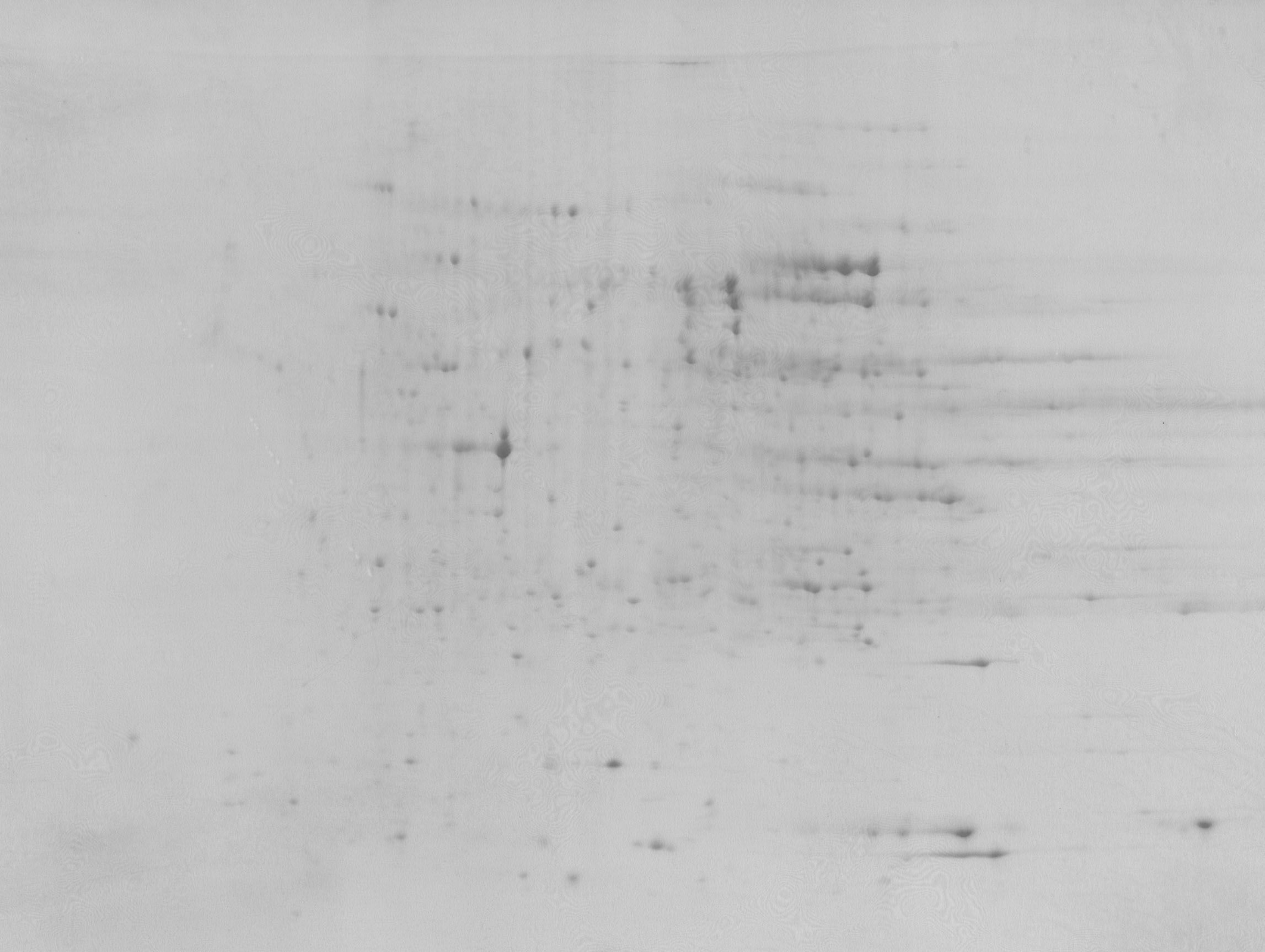

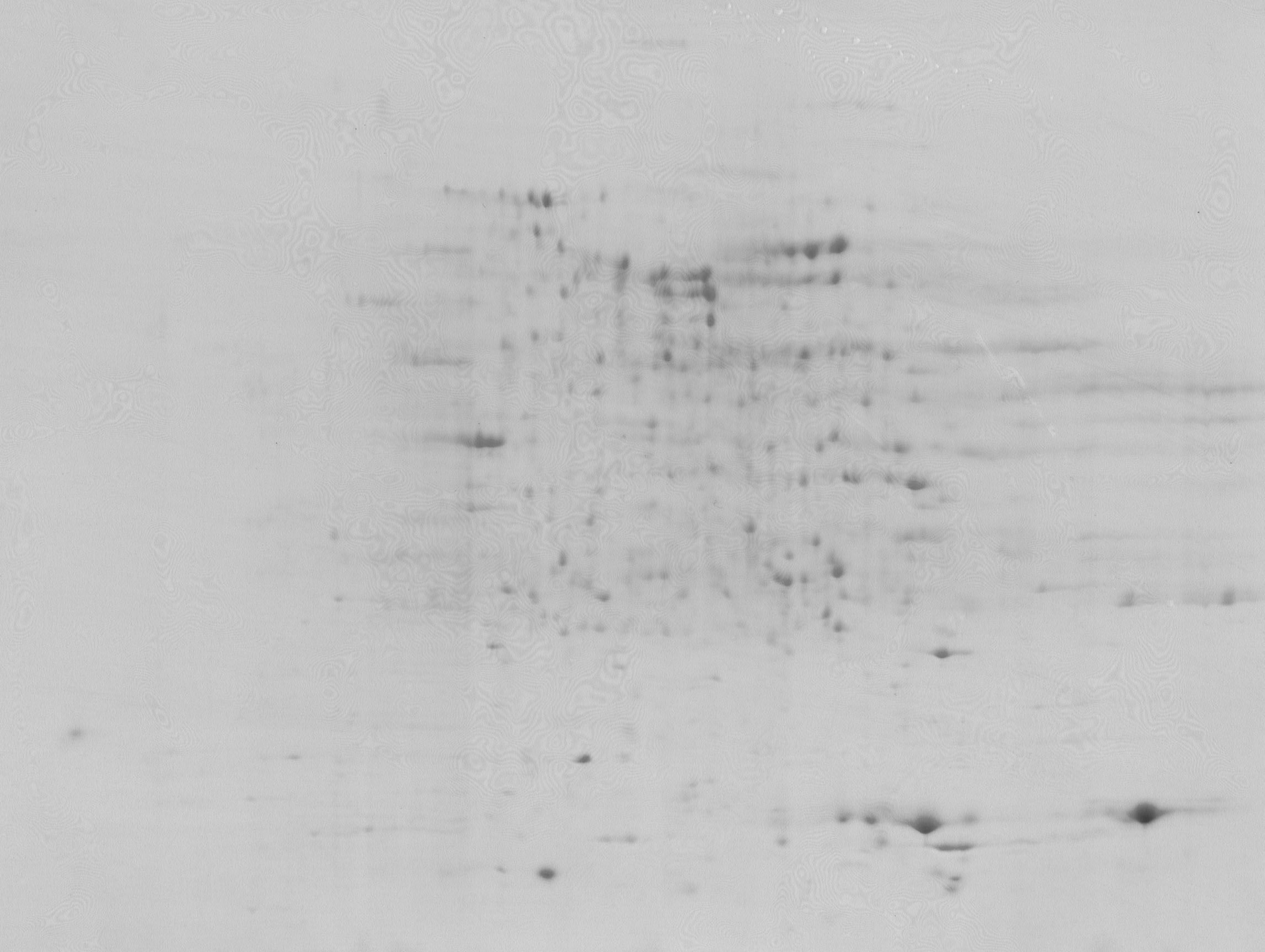

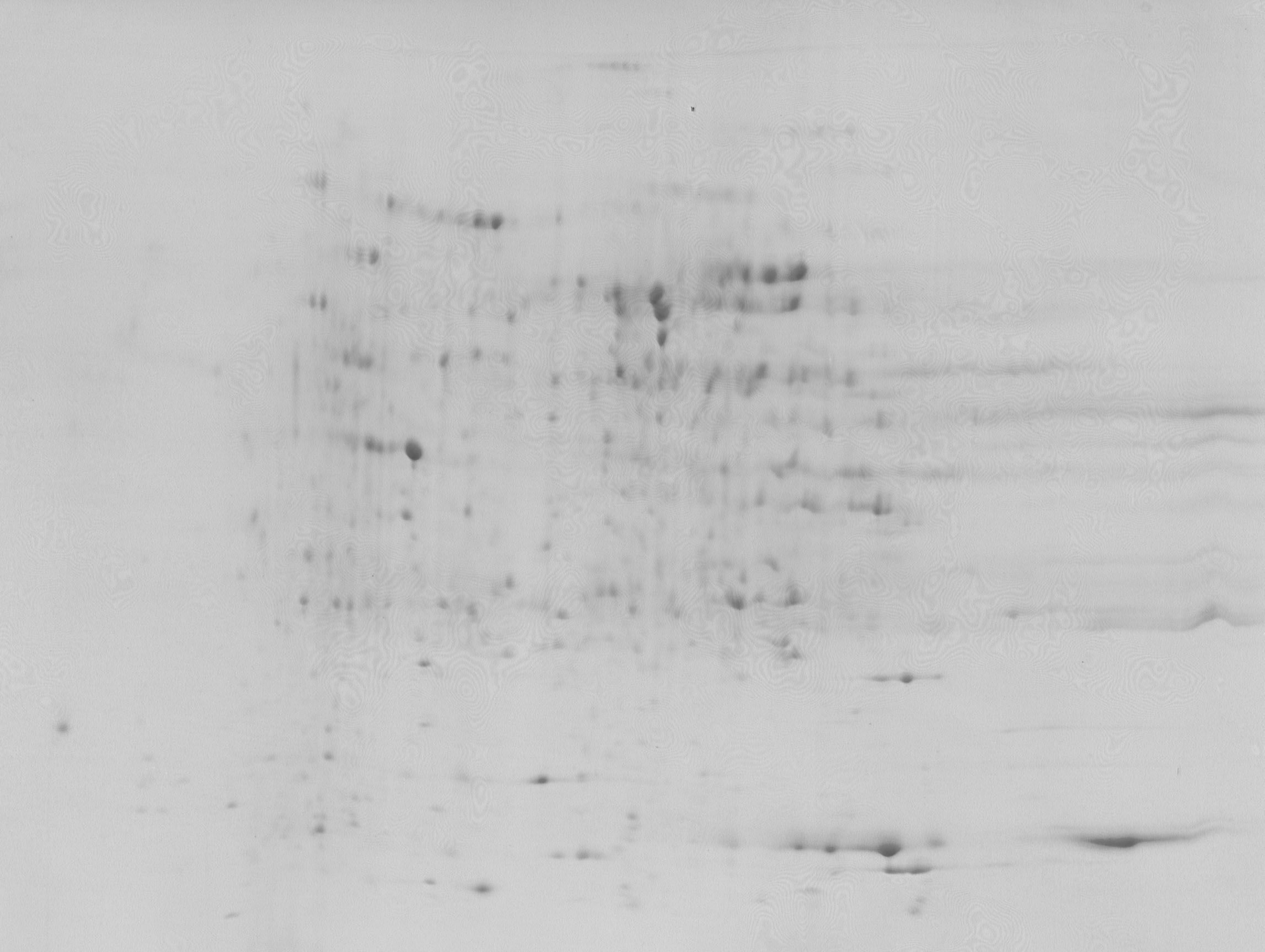

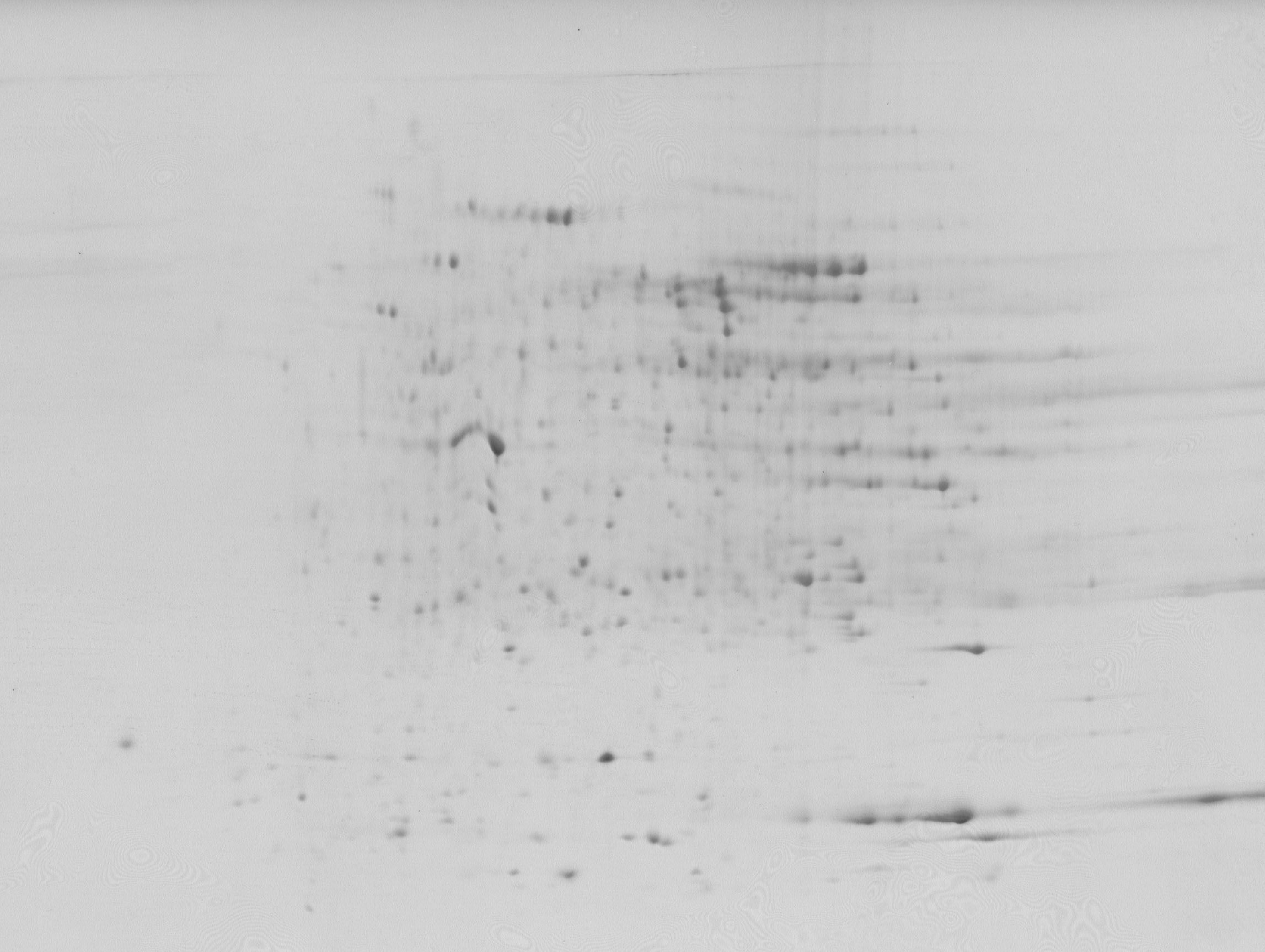

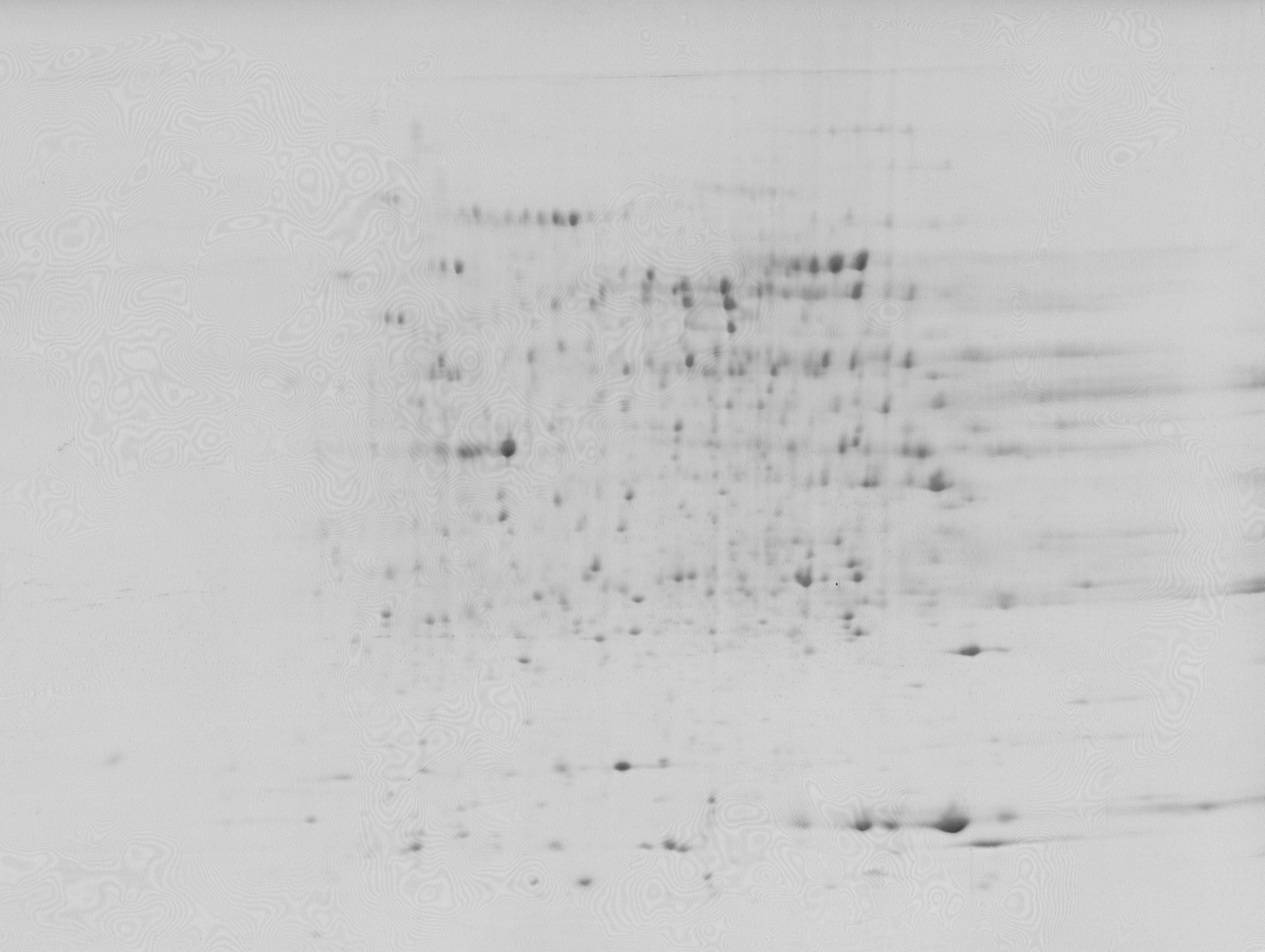

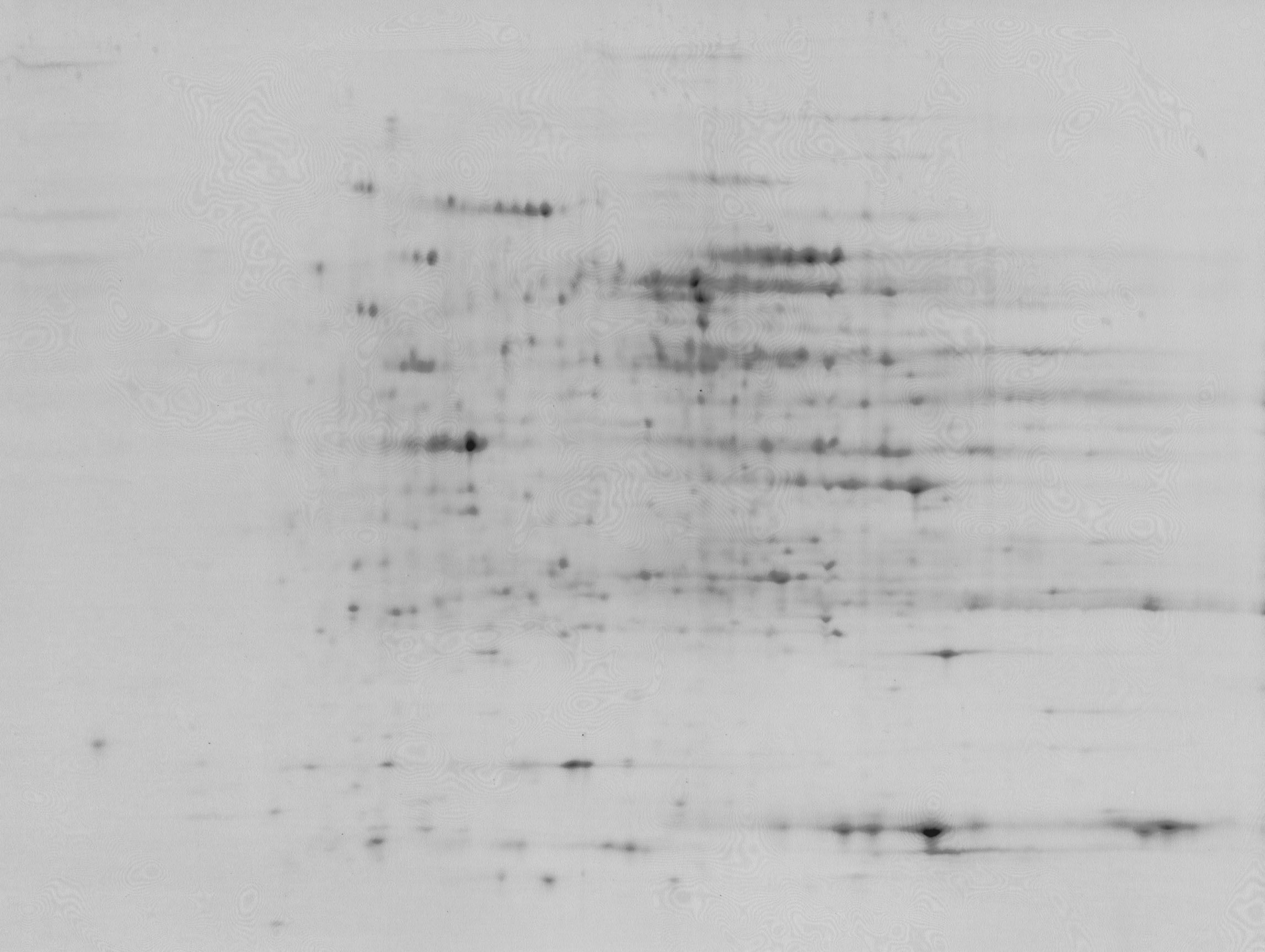

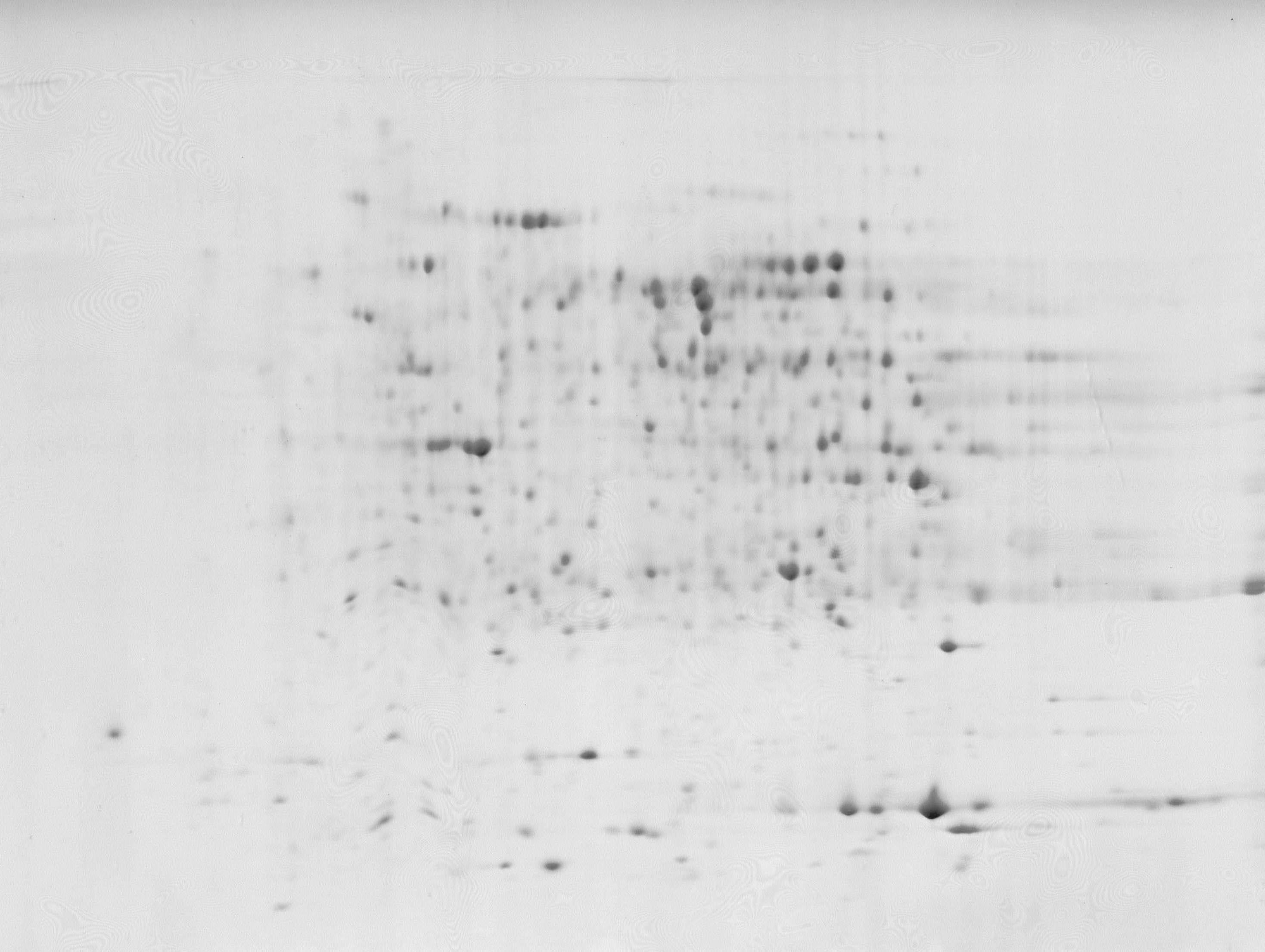

Supplement: Supplementary file 1 — Supplementary Information. [file 41598_2022_22536_MOESM1_ESM.pdf]
